# Comprehensive medicinal chemistry survey highlights a portfolio of lead molecules for Alzheimer’s disease therapy

**DOI:** 10.3389/fchem.2025.1642190

**Published:** 2025-10-01

**Authors:** Pravin R. Bhansali, Shashank M. Sonkusare, Shubhangi S. Savale, Yasanandana S. Wijayasinghe, Yini Liao, Douglas C. Sloan, Ganesh U. Chaturbhuj, Brian S. Muntean

**Affiliations:** 1 Department of Science, Faculty of Science and Technology, Alliance University, Bengaluru, Karnataka, India; 2 Department of Pharmaceutical Sciences and Technology, Institute of Chemical Technology, Mumbai, Maharashtra, India; 3 Organization, Dr. Reddy’s Laboratories, Hyderabad, Telangana, India; 4 Department of Biochemistry and Molecular Biology, Indiana University School of Medicine, Indianapolis, IN, United States; 5 Department of Pharmacology and Toxicology, Medical College of Georgia, Augusta University, Augusta, GA, United States

**Keywords:** Alzheimer’s disease, dementia treatment, drug design, medicinal chemistry, neurobiology of disease

## Abstract

The World Health Organization reports 10 million new patients with dementia each year. The most common form of dementia is Alzheimer’s disease (AD), which constitutes up to 70% of cases. AD is mainly characterized by loss of memory, which, in addition to its debilitating individual effect, represents a burden of 1.3 trillion US dollars globally. The staggering scale of hardship has spurred intense investigations from the scientific community in search of therapeutic solutions. Recent advances to combat AD involve the identification of numerous neural targets and concomitant chemical interventions as nodes of therapy. Due to disparate biological and chemical facets of AD therapy, a comprehensive perspective covering both arenas is currently missing from the literature. This perspective aims to provide an extensive understanding of anti-AD mechanics alongside small-molecule drug design efforts from a medicinal chemist viewpoint. We are confident that this survey of the literature will provide a resourceful motivation to propel future research efforts towards successful Alzheimer’s disease therapy.

## 1 Introduction

Dementia is characterized by a progressive decline in memory and cognitive function, which impacts over 55 million individuals worldwide (Alzheimer’s and Dementia, 2024). Alzheimer’s disease (AD) stands as the foremost cause of dementia, and therefore, the quest for effective treatments has never been more urgent. Key risk factors include advanced age and genetic predisposition, thus placing a substantial burden on healthcare systems and families alike. Although preventive strategies such as maintaining social and physical engagement offer some hope, a few symptomatic treatments are available. Thus, the search for curative interventions remains an unmet challenge. The complexity of AD’s neuropathology, which obscures the underlying etiology, has prompted researchers to explore novel avenues for therapeutic interventions. The primary pathological hallmarks include the accumulation of misfolded proteins, namely, amyloid-β (Aβ) protein aggregates and neurofibrillary tangles (NFTs) in the brain. These protein aggregates not only affect neurons but also other critical cell types, such as astrocytes and microglia, which facilitates the relentless progression of AD sequelae. Here, we have catalogued a portfolio of synthetic chemical molecules that have been leveraged for potential interaction with biological targets in AD in the central nervous system.

## 2 Alzheimer’s disease

**FIGURE 1:**
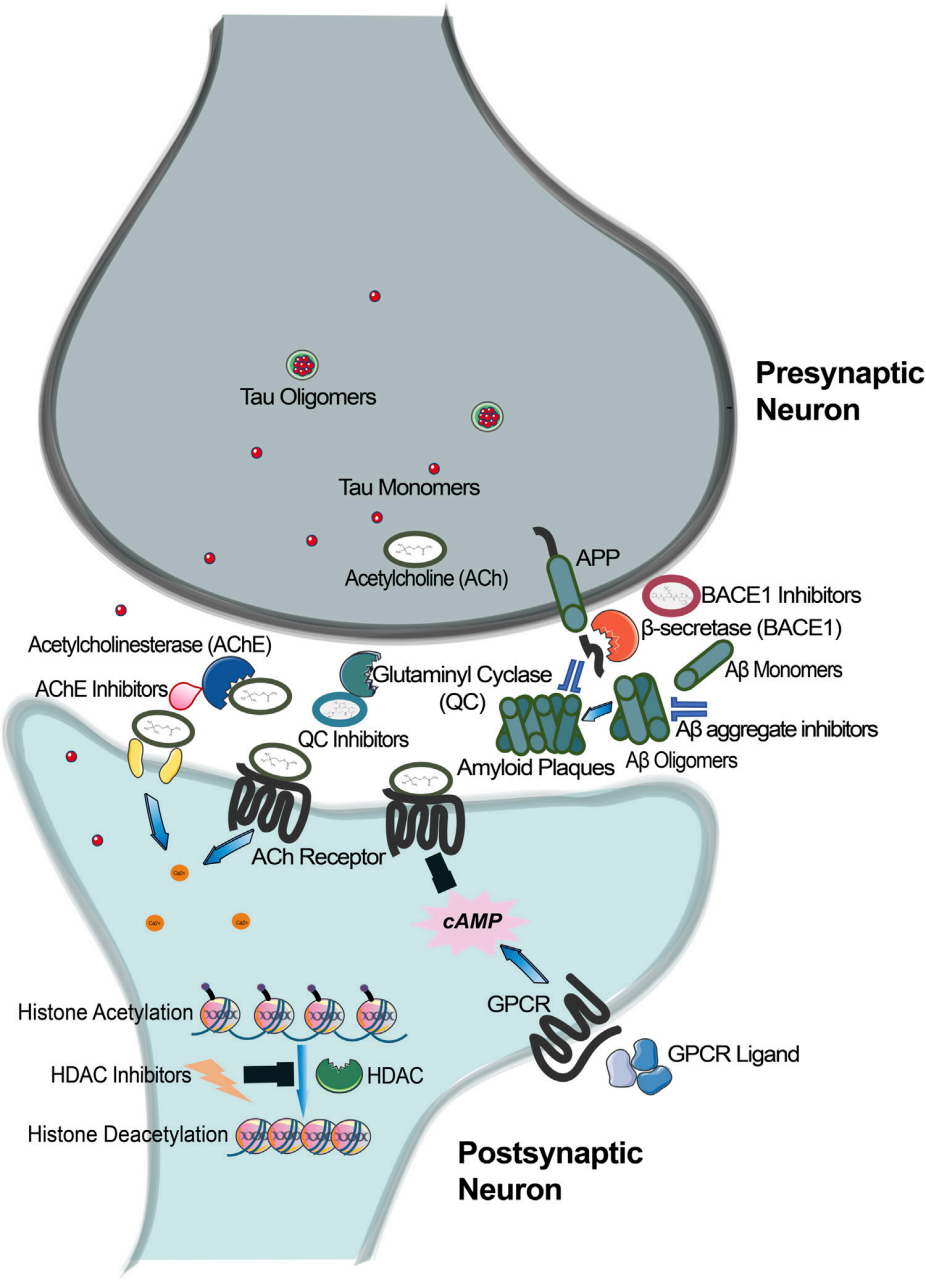
An Array of Biological Targets for AD. Progressive loss of cholinergic neurons in AD leads to reduced levels of acetylcholine (ACh). Because Acetylcholinesterase (AChE) degrades and further lowers ACh levels, AChE inhibition may have therapeutic benefit in AD. Cleavage of the amyloid precursor protein by BACE-1 produces Aβ peptides, which may accumulate and lead to neurotoxicity. Prevention of Aβ buildup would mitigate AD pathology. Direct BACE-1 inhibition or molecules that segregate Aβ plaques are nodes of therapeutic potential. Glutamine Cyclase catalyzes the pyroglutamate posttranslational modification on Aβ peptides, which renders Aβ insensitive to degradation. Inhibition of the processes represents another therapeutic opportunity. Regulation of gene expression via HDAC modulators has potential to impact synaptic plasticity important for memory. GPCRs drive the cAMP second messenger cascade, which prominently impacts downstream enzymes and transcriptional machinery important for brain function.

AD is characterized by age-associated gradual loss of memory and cognition (Rosini et al., 2005) and is the leading cause of dementia, which affects more than 55 million people globally. Although preventative factors may include a handful of symptomatic treatment options and frequent social or physical activity, curative therapies are not presently available (Qiu et al., 2009). The neuropathology of AD is multi-faceted, which shrouds the primary causes of the disease; however, contributing genetic conditions have been discovered that are thought to have strong neuropathological determinants (Figure 1). The most apparent condition is the accumulation of Aβ plaques and neurofibrillary tangles, which have both been observed in postmortem studies (Rosini et al., 2005). Aβ plaques are insoluble protein deposits that are formed when amyloid precursor protein (APP) is cut in succession via the action of two enzymes, namely, γ-secretase and β-secretase. The cleaved less-soluble Aβ peptides then aggregate extracellularly, creating Aβ oligomers and plaques in the brain, primarily in the cortex, which interrupt and dampen synapse signalling, often manifesting in the symptom of poor memory. However, neurons are not the only affected cell type as AD can also affect astrocytes and microglia. The interaction of microglia with Aβ can release cytokines that are toxic to neurons and can also initiate phagocytosis (Breijyeh and Karaman, 2020). The other major pathology of AD is the presence of NFTs in neurons. These structures begin to form with an abnormal modification (i.e., hyperphosphorylation) of tau protein. Tau is known to stabilize microtubules in the neurons, but after its hyperphosphorylation, it dissociates, misfolds and relocates to the soma. Misfolded tau proteins have the potential to travel via synapse to neighbouring neurons, spreading further cell damage (Wu et al., 2021). Although curative options for AD are farther from the reach of medicinal chemists, the following few targets provide some hope to treat symptoms of AD and thus prevent further deterioration.

## 3 Biology

### 3.1 Cholinesterase inhibitors

**FIGURE 2:**
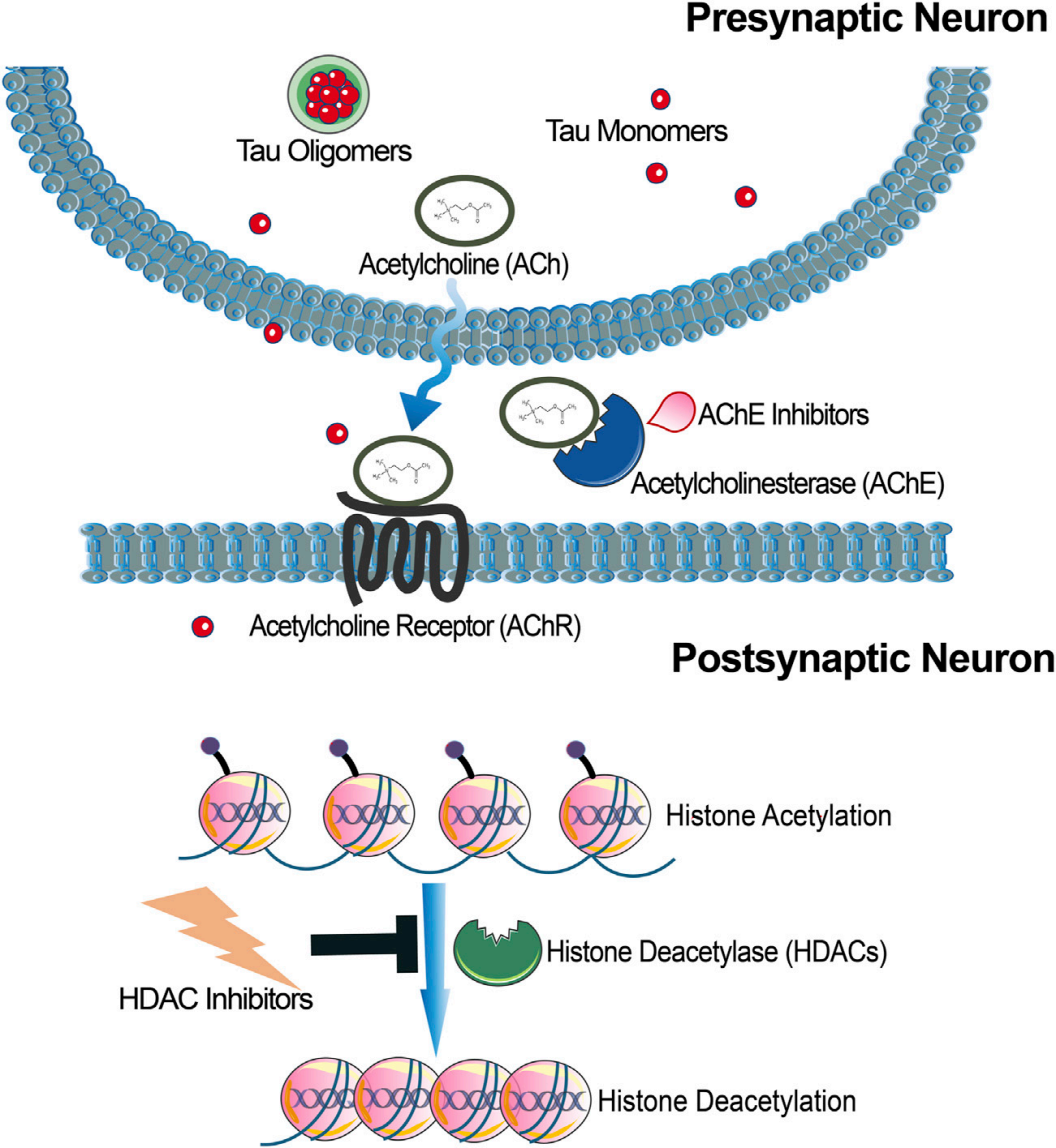
Schematic illustration of targeting either Acetylcholinesterases (AChE) or histone deacetylases (HDAC) as AD therapeutics. Inhibition of AChE would increase synaptic acetylcholine levels, which have been found to be reduced in some cases of AD. HDACs regulate gene transcription and therefore HDAC interference could promote expression of proteins important for learning and memory.

Acetylcholine (ACh) is the key neurotransmitter involved in cholinergic neurotransmission, which is vital for various cognitive functions, including memory formation and consolidation (Hasselmo, 2006). During learning, cholinergic neurons release ACh to promote the encoding of new memories (Palacios-Filardo and Mellor, 2019). Increased ACh levels in the hippocampus facilitate the consolidation of information into long-term memory (Haam and Yakel, 2017). Optimal levels of ACh in the prefrontal cortex help modulate attentional processes, allowing individuals to concentrate on relevant stimuli and filter out distractions. ACh modulates synaptic plasticity, the ability of synapses to undergo long-lasting changes in strength, through various mechanisms (Obermayer et al., 2017; Palacios-Filardo and Mellor, 2019; Picciotto et al., 2012). One way ACh influences synaptic plasticity is through modulation of Long-Term Potentiation (LTP) (Fernández De Sevilla et al., 2008; Kassab, 2023). LTP, a strengthening of synaptic connections, is a cellular process associated with learning and memory formation. ACh can enhance the induction and maintenance of LTP in certain brain regions. Activation of muscarinic receptors, specifically the M1 subtype, by ACh facilitates the generation of LTP (Dennis et al., 2016). ACh release during learning promotes synaptic strengthening and consolidation of memories. Another form of plasticity, Long-Term Depression (LTD), is also modulated by Ach (Sumi and Harada, 2023). LTD involves the weakening of synaptic connections. ACh can influence LTD in diverse ways depending on the brain region and receptor subtypes involved (Dickinson et al., 2009; Jo et al., 2010; Scheiderer et al., 2006; Volk et al., 2007). In addition, ACh can directly affect the strength of synaptic transmission through its action on presynaptic and postsynaptic receptors (Exley and Cragg, 2008). Activation of presynaptic nicotinic receptors by ACh can enhance neurotransmitter release (Exley and Cragg, 2008; Zhong et al., 2014), leading to an increase in synaptic strength. Postsynaptic nicotinic and muscarinic receptor activation can modulate the excitability of postsynaptic neurons (Chung et al., 2016) influencing the integration of synaptic inputs and the generation of action potentials (Figure 2; Hedrick and Waters, 2015; Ge and Dani, 2005). ACh also modulates the expression of molecules involved in signal transduction pathways associated with synaptic plasticity (Świt et al., 2023) such as protein kinases and phosphatases (Halder and Lal, 2021; Jouvenceau et al., 2006; Peters et al., 2003). Finally, interactions of ACh with other neurotransmitter systems, such as glutamate and dopamine, also modulate synaptic plasticity (Del Arco and Mora, 2005; Suzuki et al., 2001; Xiao et al., 2020). It can influence the release and effects of these neurotransmitters, further shaping synaptic plasticity processes and behaviors (Donovan et al., 2022; Lester et al., 2010). These interactions contribute to the complex regulation of synaptic strength and plasticity in multiple brain regions. Collectively, the duration and magnitude of ACh transients greatly shape the activity of neural networks. Therefore, cholinergic neurons that are abundant in the basal forebrain and hippocampus are involved in learning, memory, attention, and other cognitive processes. This enables cholinesterases (ChE) to modulate in cognition and memory processes in the brain (Lane et al., 2006). These enzymes, which include acetylcholinesterase (AChE) and butyrylcholinesterase (BuChE), are responsible for breaking down ACh in the synaptic cleft (Kandiah et al., 2017). In AD, there is a progressive loss of cholinergic neurons, leading to a reduction in acetylcholine levels in critical brain regions such as the basal forebrain and nucleus basalis of Meynert (Hampel et al., 2018). The degeneration and loss of cholinergic neurons in the AD brain lead to a substantial reduction in the production and release of ACh. Hence, therapeutic approaches targeting the cholinergic system, such as cholinesterase inhibitors (ChEI) (Rozzini et al., 2007) aim to alleviate the cognitive symptoms in AD by increasing acetylcholine levels and enhancing cholinergic neurotransmission (Anand and Singh, 2013; Hampel et al., 2018). Although these treatments do not halt or reverse the underlying cholinergic neuron degeneration, the augmentation of acetylcholine levels improves cognitive processes such as memory, attention, and learning in individuals with AD (Li et al., 2015).

### 3.2 Aβ aggregate inhibitors

**FIGURE 3:**
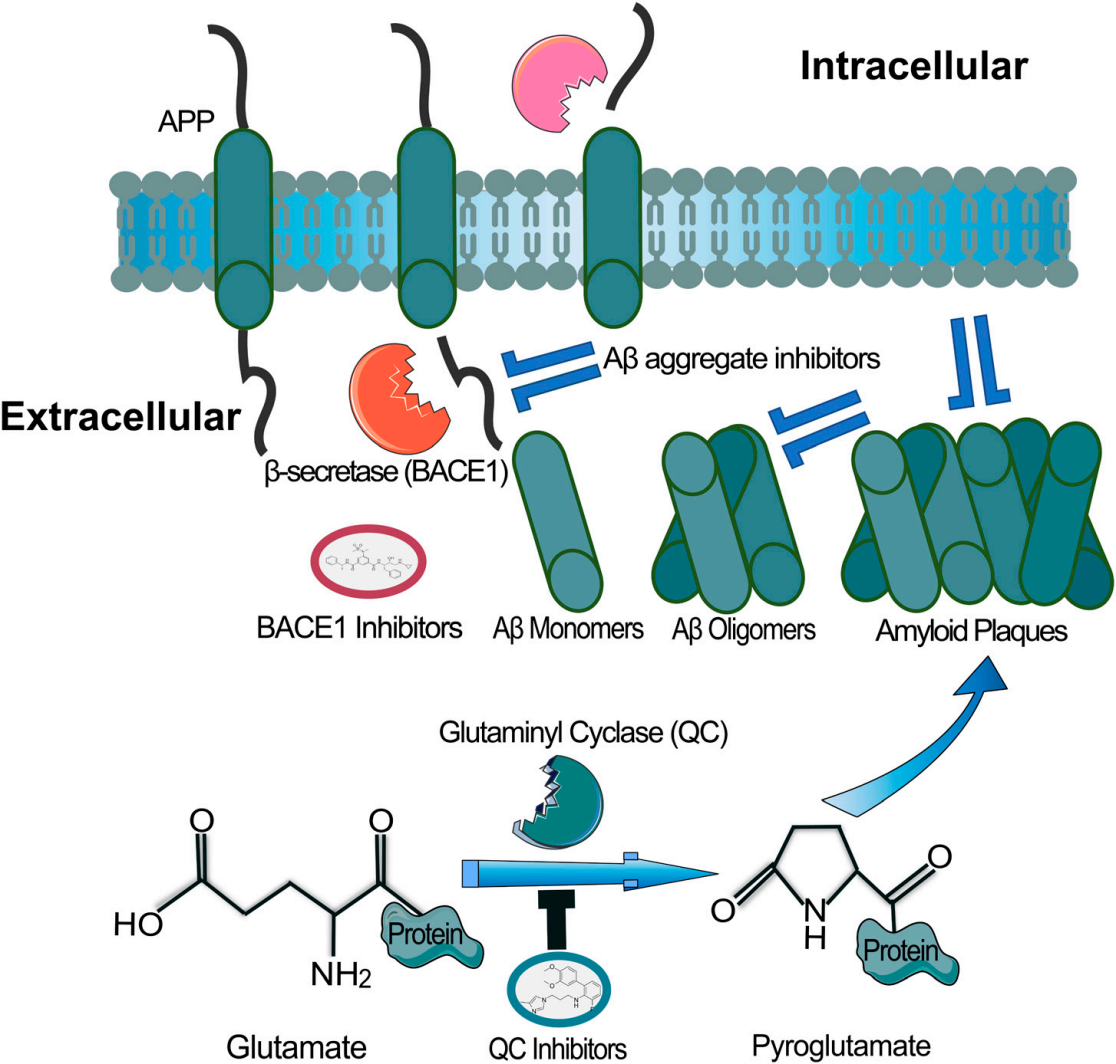
Accumulation of Aβ peptides leads to insoluble plaques that are a hallmark of AD. Aβ oligomers may be formed from BACE-1 mediated cleavage of amyloid precursor protein. Inhibition of BACE-1 would therefore prevent initial Aβ peptides. Existing Aβ plaques may be disrupted by molecules or antibodies that promote dissociation. The pyroglutamate protein modification, rendered by Glutaminyl Cyclase (GC), promotes stability of Aβ plaques. Inhibition of GC is thought to provide a mechanism to help clear pathological Aβ.

β-secretase, in particular the β-site amyloid precursor protein cleaving enzyme 1 (BACE-1), contributes to the development of AD (Figure 3; Kandalepas and Vassar, 2012). It is primarily involved in the production of Aβ peptides, which are key components of the amyloid plaques developed in the brains of individuals with AD (Masters et al., 1985; Neumann et al., 2018). BACE-1 is responsible for cleaving the amyloid precursor protein (APP) at the β-site, leading to the generation of soluble fragments called β-CTF (C-terminal fragment) (Hunt and Turner, 2009). Subsequently, γ-secretase cleaves β-CTF to produce Aβ peptides of varying lengths, including the toxic Aβ42 form (Hur, 2022). The accumulation of Aβ42 peptides, also known as Aβ aggregates or amyloid aggregates, is believed to be a critical step in the development of amyloid plaques (clumps or deposits of Aβ peptides), a hallmark pathological feature of AD. Aβ peptides tend to misfold and aggregate, leading to the formation of insoluble protein deposits (Frisoni et al., 2022). The specific Aβ peptide involved in aggregation is Aβ42, which has a greater propensity to form aggregates compared to Aβ40. These aggregates take on different forms, including soluble oligomers, protofibrils, and fibrils (Zhang et al., 2011). The most well-known and visible form of Aβ aggregates in AD is the formation of amyloid plaques (Holsinger et al., 2013). These plaques consist of dense accumulations of Aβ fibrils that are insoluble and resistant to degradation. They are typically found in the spaces between neurons in the brain, disrupting normal neuronal functions (Siwecka et al., 2023). Aβ aggregates can also exist in smaller, soluble oligomeric forms. These oligomers are considered to be highly toxic to neurons and are thought to contribute to synaptic dysfunction and neuronal damage. Hence, Aβ oligomers are believed to have a greater impact on cognitive impairment than the fibrillar plaques themselves. Aβ aggregates can interact with and influence the aggregation of tau protein, another key pathological feature of AD. The tau protein stabilizes microtubules in neurons, and its abnormal aggregation leads to the formation of neurofibrillary tangles (Naseri et al., 2019). Aβ aggregates have been found to promote tau aggregation and contribute to neurodegeneration. Aβ aggregates, including both plaques and oligomers, are therefore associated with neurotoxic effects in AD (Lane et al., 2018). The presence of Aβ aggregates, particularly amyloid plaques, is often used as a biomarker for AD diagnosis. Imaging techniques such as positron emission tomography (PET) can detect the accumulation of Aβ plaques in the brain, helping to differentiate AD from other forms of dementia (Thijssen et al., 2021). Therefore, Aβ aggregate inhibitors aim to prevent the formation, promote the disaggregation, or enhance the clearance of such Aβ aggregates in AD (Weller and Budson, 2018). Monoclonal antibodies that specifically bind to Aβ aggregates have been developed to target and clear Aβ aggregates from the brain (Avgerinos et al., 2021). Examples include aducanumab, which has recently been approved by the U.S. Food and Drug Administration (FDA), and solanezumab and gantenerumab, which are currently being evaluated in clinical trials. These antibodies can potentially facilitate the removal of Aβ aggregates through immune-mediated mechanisms or by enhancing their clearance by microglial cells (Shi et al., 2022). There has also been the implication of metals (copper, zinc, and iron) in promoting Aβ aggregation and neurotoxicity (Das et al., 2021). Metal chelators can bind to these metals and prevent their interaction with Aβ, thereby inhibiting or disrupting the aggregation process (Liu et al., 2019). Some examples of metal chelators include clioquinol and PBT2, which have been investigated in preclinical and clinical studi (Lei et al., 2021) Here in this article, we will focus on small molecule anti-aggregation compounds.

### 3.3 BACE-1 inhibitors

Studies have shown that BACE-1 is elevated in the AD brains (Singh et al., 2022; Zhao et al., 2007) This increased activity leads to higher production of Aβ peptides, particularly Aβ42, which have a greater propensity to aggregate and form plaques (Sadleir et al., 2016) The overproduction and deposition of Aβ peptides contribute to neurotoxicity and the progression of AD pathology. Mutations in the genes involved in APP processing represent a risk factor for early-onset AD (Armstrong, 2019). One important causative factor in this regard is Presenilin-1 (*PSEN1*), which is part of the γ-secretase pathway (Bagaria et al., 2022). Over 300 *PSEN1* mutations have been identified (Yang et al., 2023) and thus their contribution toward pathogenesis in rare cases of familial AD (FAD) and early-onset AD are areas of active investigation (Wijeratne et al., 2023). One prominent mutation associated with FAD is the Swedish mutation (also known as the APP670/671 mutation) (Hellström-Lindahl et al., 2009). This mutation alters the BACE-1 cleavage site in the APP gene, resulting in increased production of Aβ peptides. The Swedish mutation has been extensively studied and has provided valuable insights into the role of BACE-1 in AD pathogenesis. Several other BACE-1 mutations have been identified in FAD cases. These mutations lead to increased BACE-1 activity or alter the enzyme’s processing and trafficking. Each mutation may have a specific effect on BACE-1 function, resulting in varied consequences for Aβ production and AD pathology (Bagaria et al., 2022). Collectively, BACE-1 has emerged as a promising therapeutic target for AD (Figure 3). Inhibiting BACE-1 activity could potentially reduce the production of Aβ peptides and slow down the progression of the disease. Researchers have been actively working on developing BACE-1 inhibitors as a potential treatment strategy. However, clinical trials investigating BACE-1 inhibitors have faced challenges, including safety concerns and limited efficacy, underscoring the complexity of targeting this enzyme in AD (Bazzari and Bazzari, 2022; Munj and Patil, 2022; Vassar et al., 1999). For instance, an aspect of BACE-1 inhibitors is selectivity for BACE-1 over other related enzymes. While BACE-1 is the primary enzyme responsible for β-secretase activity, other enzymes, such as BACE-2, share some similarities in structure and function. The selectivity of BACE-1 inhibitors helps minimize potential off-target effects by specifically targeting BACE-1 without interfering with the physiological functions of other related enzymes. While reducing BACE-1 activity may reduce Aβ production and hence AD pathology, it is important to consider the physiological functions of BACE-1. BACE-1 is involved in the processing of other substrates besides APP, and complete inhibition may have unintended consequences. Balancing the reduction of Aβ production with the preservation of normal BACE-1 functions remains a focus of ongoing research.

Other biochemical assays are employed for more benchmark determining factors related to AD, foremost: BACE-1 activity, the protease which cleaves APP that can form insoluble protein deposits. To detect this activity, an assay was created featuring a genetically modified proenzyme with activity dependence on BACE-1 (Verheijen et al., 2006). Once active, the enzyme can be measured at a sensitive threshold. This assay not only displays utility in detecting elevated BACE-1 activity in brain tissue but may also be useful in screening for potential drug therapies (Verheijen et al., 2006).

### 3.4 Glutamine cyclase inhibitors

Glutamine cyclase, also known as glutaminyl cyclase (QC), is an enzyme involved in the post-translational modification of certain peptides and proteins (Schilling et al., 2007). QC’s primary function is to catalyze the cyclization of *N*-terminal glutamine residues to form pyroglutamate (pGlu) (Fischer and Spiess, 1987). This enzymatic reaction is known as pyroglutamate formation or *N*-terminal cyclization. The process of pyroglutamate formation involves the removal of the free *N*-terminal glutamine or glutamate residue and subsequent cyclization of the resulting *N*-terminal glutaminyl or glutamyl residue. QC catalyzes the cyclization reaction by breaking the peptide bond between the *N*-terminal glutamine and the adjacent amino acid and then forming a cyclic amide bond, resulting in the formation of pyroglutamate (Coimbra et al., 2023). Pyroglutamate formation plays a role in protein stability and activity, which affects downstream functions and signalling of these molecules. Pyroglutamate formation is known to occur in a variety of peptide hormones, neuropeptides, and other bioactive peptides. Notably, the formation of pyroglutamate-modified Aβ peptides (Bsamen et al., 2018; Cynis et al., 2008) has been observed in the brains of individuals with AD (Figure 3). Although such pyroglutamate-modified Aβ peptides represent a small fraction of total Aβ, they are nonetheless thought to be highly toxic due to resistance to aminopeptidase degradation and ability to provide a seed for amyloid fibril formation (Hook et al., 2014; Nussbaum et al., 2012). Moreover, there are also reports of enhanced QC activity in the brains of people with AD (Gunn et al., 2021). Thus, these modified peptides have been suggested to contribute to the neurodegenerative processes and cognitive decline observed in the disease (Camargo et al., 2021; Jawhar et al., 2011; Wittnam et al., 2012). Inhibiting glutamine cyclase activity has been explored as a potential therapeutic strategy for certain diseases (Huang et al., 2011). For instance, in the context of Alzheimer’s disease, inhibiting glutamine cyclase could prevent the formation of pyroglutamate-modified Aβ peptides (Bayer, 2022), which are known to be more aggregation-prone and toxic than their unmodified counterparts. Several small molecules have been developed as glutamine cyclase inhibitors, and research in this area is ongoing (Hoang et al., 2017). Overall, glutamine cyclase is an important enzyme involved in the post-translational modification of peptides and proteins through the formation of pyroglutamate. Understanding its role and regulation may have implications for the development of therapies targeting specific diseases, including neurodegenerative disorders.

The upregulation of glutaminyl cyclase (QC) activity is strongly linked to the progression of AD; thus, QC inhibitors are a topic of interest for disease treatment. Several studies show evidence of QC inhibitors being biologically active and able to promote treatment. One, in particular, focused on QC inhibitor 23 in PC12 cells, which exhibited upregulation of heat shock proteins 70 and 90, along with the regulation of many other biochemical components related to AD pathology, such as actin gene expression. These results were analyzed with the use of biological assays such as Western blot and ELISA, along with quantitative real-time PCR (Yu et al., 2019).

### 3.5 Altering brain function by HDAC modulation

Histone deacetylase (HDAC) enzymes are important players in regulating gene expression, most notably in epigenetic regulation, with particular relevance to cognitive processes (Figure 2; Saha and Pahan, 2006; Cho and Cavalli, 2014). HDACs are responsible for removing acetyl groups from histone proteins, leading to chromatin compaction and transcriptional repression (Bhansali et al., 2011; 2014; Seto and Yoshida, 2014). This process, known as histone deacetylation, can impact the expression of genes involved in synaptic plasticity, learning, and memory formation (Latcheva et al., 2019). By altering histone acetylation patterns, HDACs modulate the transcription of genes critical for cognitive function. For instance, HDACs influence synaptic plasticity by regulating the expression of genes involved in synaptic remodeling and strengthening, such as neurotrophins, synaptic proteins, and neurotransmitter receptors (Ahmad Ganai et al., 2016). In particular, HDAC activity has been found to influence synaptic plasticity and memory formation. HDACs also play a role in neurogenesis, the generation of new neurons, in the adult brain (Guan et al., 2009; Nieto-Estevez et al., 2022). It was long believed that neurogenesis only occurred during development and early stages of life, but it has now been established that neurogenesis also takes place in specific regions of the adult brain, including the hippocampus (Cuccioli et al., 2015; Kempermann et al., 2015). Deficits in adult hippocampal neurogenesis have been observed in various neurological and psychiatric disorders, including AD. Inhibiting HDAC activity has been associated with increased neurogenesis which considered to be is a neuroprotective process (Shukla and Tekwani, 2020) suggesting a potential role in cognitive enhancement. By regulating gene expression, HDACs influence the expression of genes involved in neuronal survival, antioxidant defence, stress response, and chronic pain (Descalzi et al., 2015; Falkenberg and Johnstone, 2014). Given the involvement of HDACs in cognitive processes, HDAC inhibitors have emerged as potential therapeutic agents for cognitive disorders (Hamze, 2020; Melesina et al., 2021; Thomas et al., 2008). In preclinical studies, HDAC inhibitors have shown promising effects in enhancing synaptic plasticity, promoting memory formation, and ameliorating cognitive deficits. However, it is important to note that further research is needed to fully understand the specific roles of individual HDAC isoforms and to develop selective inhibitors with minimal side effects (Santana et al., 2023).

Finally, immunoprecipitation assays are also useful in quantifying disease components, such as tau protein aggregates, known to cause neurofibrillary tangles^.^ which are detrimental to brain function. Here, a natural alkaloid, protopine, found in plants that may exert certain biological activities, was employed for the purpose of reducing tau protein aggregate formation for memory improvement. Immunoprecipitation assays were used to study the effects of the drug’s influence on the ubiquitination of pathological tau. Fluorometric assays were useful in assessing how protopine also appears to promote the acetylation of α-tubulin, suggesting that it serves as an HDAC inhibitor (Sreenivasmurthy et al., 2022). which strongly influences neurogenerative diseases’ pathology (Sanchez and Blower, 1997).

### 3.6 Targeting GPCR-cAMP signalling axis

**FIGURE 4:**
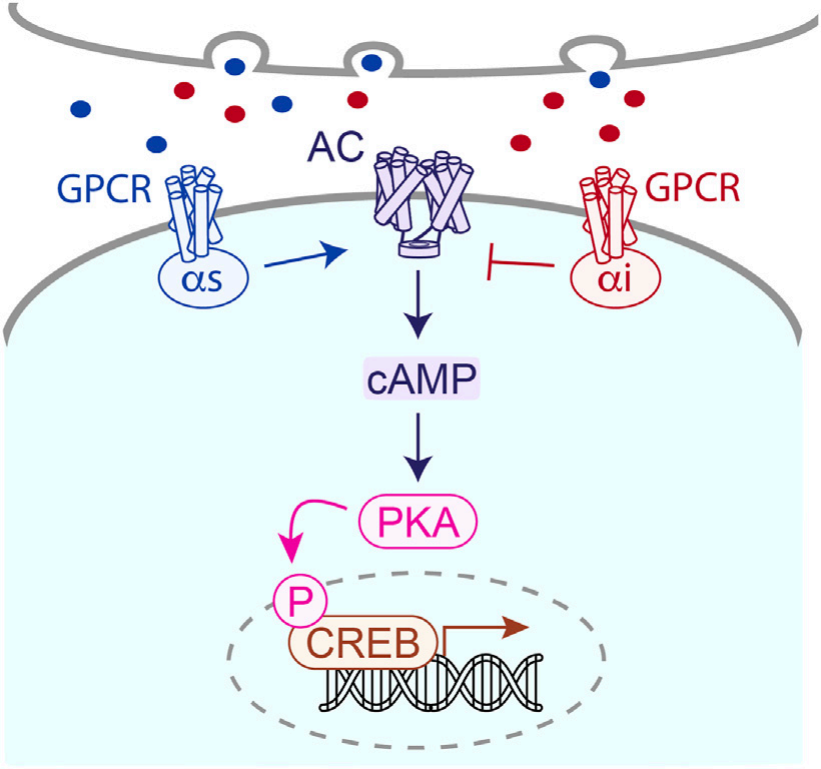
Many GPCRs feed into the cAMP cascade, which regulates kinases and transcription factors that ultimately modulate neuronal activity important for learning and memory. Adjusting cAMP via pharmacology at GPCRs is suggested to improve brain function in AD.

The regulation of BACE-1 activity by the cyclic adenosine monophosphate (cAMP) dependent signalling pathway is an emerging topic of interest in AD research (Thathiah and De Strooper, 2011). The cAMP pathway can influence BACE-1 expression at the transcriptional level (Zhao et al., 2016). Activation of stimulatory G protein-coupled receptors (GPCRs) by their ligands, such as neurotransmitters or hormones, leads to the activation of adenylyl cyclase and subsequent production of neuronal cAMP (Figure 4; Cooper, 2003; Muntean et al., 2018) Increased cAMP levels activate protein kinase A (PKA), which can phosphorylate and activate certain transcription factors, including CREB (cAMP response element-binding protein) (Kandel, 2012). Activated CREB can then bind to specific regions of the BACE-1 gene promoter and enhance its transcription, resulting in increased BACE-1 expression (Sambamurti et al., 2004). In addition to transcriptional regulation, the cAMP pathway can also modulate BACE-1 activity through post-translational mechanisms (Nowak et al., 2006; Tamagno et al., 2012). The cAMP pathway can influence the intracellular trafficking and subcellular localization of BACE-1. PKA-mediated phosphorylation of APP has been shown to affect its cellular distribution and trafficking, potentially altering its enzymatic activity and substrate accessibility (Marambaud et al., 1996). The cAMP pathway facilitates crosstalk with other signalling pathways that regulate BACE-1. For example, the cAMP pathway can interact with the Wnt/β-catenin signalling pathway, which has been implicated in AD pathogenesis. Activation of the Wnt/β-catenin pathway has been shown to regulate BACE-1 expression and Aβ production (Chen et al., 2019; Elliott et al., 2018). Understanding the regulation of BACE-1 by the cAMP pathway is important for elucidating the mechanisms underlying Aβ production in AD and for identifying potential therapeutic targets. Importantly, modulating the cAMP pathway or targeting specific GPCRs involved in its activation could be explored as a strategy to modulate BACE-1 expression and activity, ultimately reducing Aβ production in AD. However, it is worth noting that the cAMP pathway is complex and can have pleiotropic effects in different cell types and brain regions, so careful consideration of specificity and potential side effects is crucial in developing targeted interventions. Further research is needed to fully uncover the intricate regulatory mechanisms and their therapeutic implications in AD. Nonetheless, targeting GPCRs has emerged as a potential therapeutic strategy for AD. The cholinergic system, particularly the muscarinic acetylcholine receptors (mAChRs), has been a focus for GPCR-based therapies in AD (as noted above). The loss of cholinergic neurons and a decrease in acetylcholine levels are prominent features of AD. By targeting mAChRs, it is possible to modulate cholinergic neurotransmission and enhance cognitive function. Selective agonists of the M1 and M2 subtypes of mAChRs have been investigated for their potential in improving cognitive deficits in AD. (Brown et al., 2021; Sanjay et al., 2022) Activation of the M1 receptor subtype has shown cognition-enhancing effects by increasing synaptic plasticity, improving memory formation, and promoting neuroprotective mechanisms. M2 receptor activation can also have beneficial effects by modulating neurotransmitter release and reducing Aβ peptide production. Beyond the cholinergic system, other GPCRs have also been investigated as potential targets for AD therapy. For example, the metabotropic glutamate receptors (mGluRs) (Abd-Elrahman et al., 2021) particularly the mGluR5 subtype, have been targeted to modulate glutaminergic signalling and synaptic function (Kumar et al., 2015). Activation of certain serotonin receptors, such as 5-HT6 receptors, has also shown potential in improving cognitive impairments (Benhamú et al., 2014; Czarnota-Łydka et al., 2022; Mdawar et al., 2020). Given the complex nature of AD pathology, combination therapies targeting multiple GPCRs or GPCRs alongside other therapeutic approaches are being explored. Adenosine receptors, particular the A1 and A2A subtype, have recently emerged as putative neuronal GPCR targets in AD (Trinh et al., 2022). Given the role of the noradrenergic system in cognition (Borodovitsyna et al., 2017) and adjusted norepinephrine levels in AD brains (Mann et al., 1982) adrenergic dysfunction in AD represents additional putative GPCR targets (Gannon et al., 2015). The orphan receptors GPR3 and GPR6, of which the endogenous ligand has not yet been uncovered, are additional GPCRs that have gained traction in AD research. [127]. GPR3 overexpression enhances Aβ production while receptor depletion prevents Aβ (Huang et al., 2015; Ricardo and Lehmann, 2009). On the other hand, GPR6 is thought to facilitate neuroprotective effects in AD through the complement pathway (Huang et al., 2015; Ricardo and Lehmann, 2009). Combining treatments that target distinct aspects of the disease, such as Aβ production, neuroinflammation, and synaptic dysfunction, may offer synergistic effects and better therapeutic outcomes. It is important to note that while targeting GPCRs holds promise for AD treatment, the development of GPCR-based therapies is still in the early stages, and much more research is needed to validate their efficacy, safety, and long-term effects in clinical settings. Additionally, the heterogeneity of AD and the diverse functions of GPCRs require careful consideration of patient selection and personalized treatment approaches.

Monitoring cAMP dynamics offers critical insight toward signal transduction, and therefore information transfer, between neuronal circuits. Utilization of genetically encoded cAMP biosensors is beneficial to understanding signaling logistics in the native neuronal environment, as successfully employed in various models of neuronal pathology (Masuho et al., 2018; Muntean et al., 2019; 2021; Sutton et al., 2019). The approach is empowered by the *cAMP E*ncoded *R*eporter (*CAMPER*) mouse model that conditionally expresses the FRET-based TEpacVV cAMP biosensor (Muntean et al., 2018). Microglia are of particular importance in the understanding or AD and therapeutic potential because microglial GPCRs are involved in the degradation of amyloid plaques (Haque et al., 2018). This interplay allows for a range of study approaches, especially in respect to neuromodulatory second messengers like cAMP.

## 4 Medicinal chemistry

### 4.1 Cholinesterase inhibitors

**FIGURE 5:**
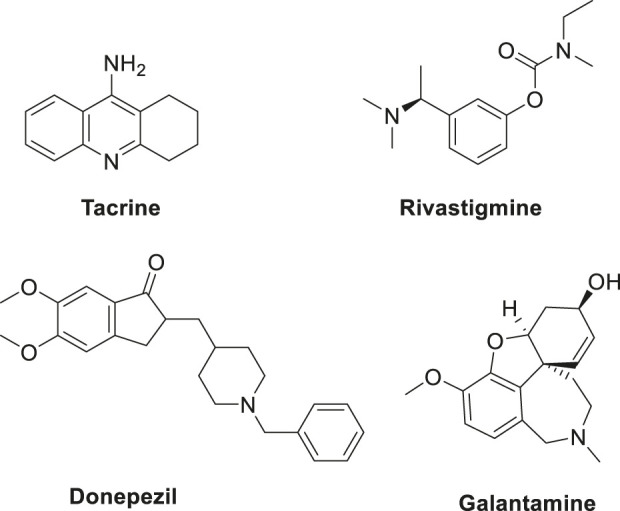
Various approved drugs for AD treatment.


Cholinesterases such as acetylcholinesterase (*AChE*) and butyrylcholinesterase (*BuChE*) are established drug targets for treatment of Alzheimer’s disease. Various approved drugs targeting cholinesterases, such as tacrine, rivastigmine, donepezil, and galantamine (Figure 5), are available, but these drugs have certain limitations.

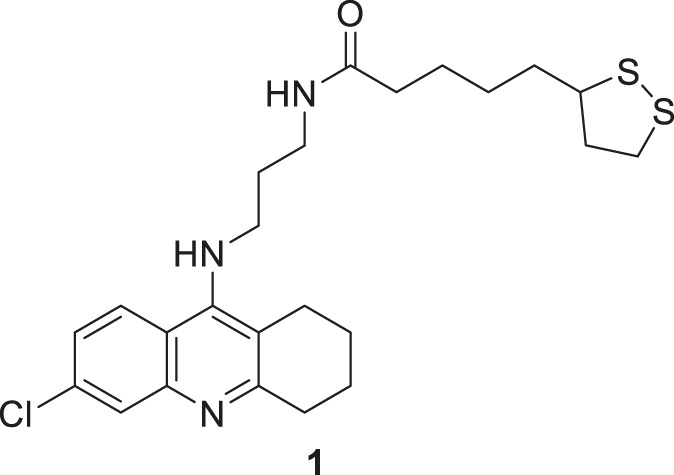


Despite limitations, tacrine is an important scaffold for developing new potent anti-Alzheimer’s agents. Rosini et al. have designed and evaluated a new tacrine and a lipoic acid hybrid called lipocrine. Compound **1** showed good anti-cholinesterase activity. Compound **1** (*N*-(3-((6-chloro-1,2,3,4-tetrahydroacridin-9-yl)amino)propyl)-5-(1,2-dithiolan-3-yl) pentanamide), a chlorine-substituted tacrine, has three carbon containing chain between lipoic acid. It exhibited low nanomolar inhibitory activity against *AChE* (IC_50_ = 0.253 ± 0.016 nM) and *BuChE* (10.8 ± 2.5 nM) in the Ellman’s assay. Lipocrine is one of the first compounds that inhibit *AChE* and *AChE*-induced Aβ aggregation and protects against oxidative radical species (Rosini et al., 2005).


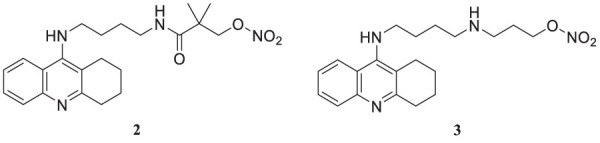


Fang et al. synthesized and evaluated 14 NO-donor-tacrine hybrids having anti-Alzheimer’sAlz ‘s’activity. All of them exhibited promising cholinesterase inhibitory activity *in-vitro*. While compound **2** (2,2-dimethyl-3-oxo-3-((4-((1,2,3,4-tetrahydroacridin-9-yl)amino)butyl)amino) propyl nitrate) depicted high selectivity towards butyrylcholinesterase (IC_50_ = 7.3 ± 2.0 nM) than acetylcholinesterase (IC_50_ = 226.0 ± 91 nM) with a 31-fold selective ratio. Compound **2** binds more efficiently to *BuChE* than *AChE* due to the steric hindrance of the bulky alkylenediamine spacer. Hepatotoxicity associated with tacrine is the main concern of its prolonged use. In that regard, compound **3** (3-((4-((1,2,3,4-tetrahydroacridin-9-yl)amino)butyl)amino)propyl nitrate) exhibited good *AChE* inhibitory activity (IC_50_ = 5.6 ± 0.7 nM) and was less hepatotoxic than tacrine (Fang et al., 2008).


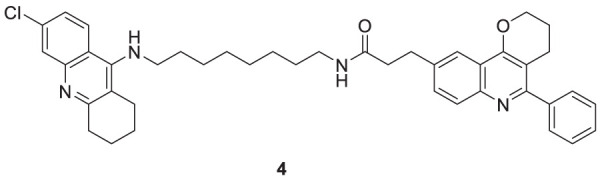


Camps et al. designed, synthesized, and evaluated a series of dual-binding site *AChE* inhibitors. *N*-(8-((6-chloro-1,2,3,4-tetrahydroacridin-9-yl)amino)octyl)-3-(5-phenyl-3,4-dihydro-2*H*-pyrano [3,2-*c*]quinolin-9-yl)propenamide (**4**) demonstrates potent activity towards inhibition of *AChE* (IC_50_ = 14 ± 1.2 nM). The newly discovered compounds comprise chlorotacrine connected to a pyrano [*3,2-c*]-quinoline structure through an oligomethylene linker and an amido group. They exhibited a dual binding effect, binding to the active site through tacrine and to a peripheral site through the pyrano [*3,2-c*]-quinoline moiety. Compound **4** crosses the blood-brain barrier and shows *in-vitro* inhibition of Aβ aggregation (Camps et al., 2009).


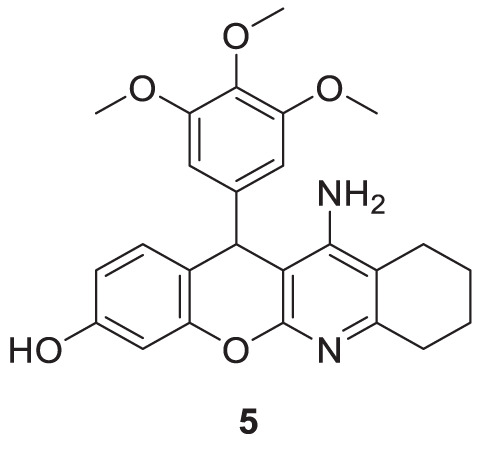


Oset-Gasque et al. developed a non-competitive inhibitor chromenotacrines **5** (11-amino-12-(3,4,5-trimethoxyphenyl)-7,9,10,12-tetrahydro-8*H*-chromeno [2,3-*b*]quinolin-3-ol), which is less toxic to human liver cells than tacrine. Compound **5** was identified as a potential *AChE* inhibitor (IC 50 = 0.041 ± 0.001 µM) binding to the peripheral anionic site. Compound **5** exhibited selective inhibition towards *AChE,* and kinetic studies revealed moderate brain permeability (Oset-Gasque et al., 2014).


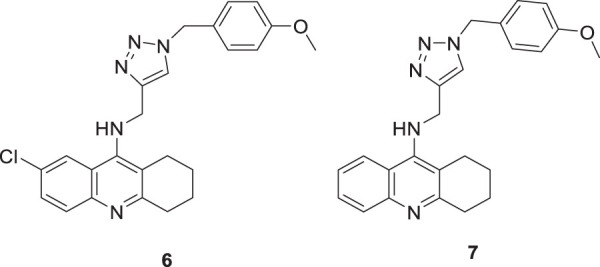


Najafi et al. designed and evaluated new tacrine hybrids introducing triazine substitution at the amine group of tacrine. Compounds **6** (7-chloro-*N*-((1-(4-methoxybenzyl)-1*H*-1,2,3-triazol-4-yl)methyl)-1,2,3,4-tetrahydroacridin-9-amine) and **7** (*N*-((1-(4-methoxybenzyl)-1*H*-1,2,3-triazol-4-yl)methyl)-1,2,3,4-tetrahydroacridin-9-amine) illustrated anti-cholinesterase activity. Although both compounds **6** and **7** have 4-methoxyphenyl connected to a 1,2,3-triazole moiety, compound **6** with chlorine substitution at 7-position of acridine ring displayed the best *AChE* inhibitory activity (IC_50_ = 0.521 ± 0.025 µM), and compound **7** indicated activity against *BuChE* (IC_50_ = 0.055 ± 0.012 µM). This reveals that these small structural changes lead to changes in the preferential binding pattern of the enzyme. The compounds were evaluated for neuroprotection and radical scavenging ability. Compound **6** showed moderate neuroprotective activity at 10 mM (cell viability = 65.40% and P < 0.05 vs. H_2_O_2_ treatment alone), and both compounds **6** and **7** indicated no notable antioxidant activities. Tacrine-1,2,3-triazole hybrids are considered potential agents with anti-cholinesterase activity (Najafi et al., 2017).


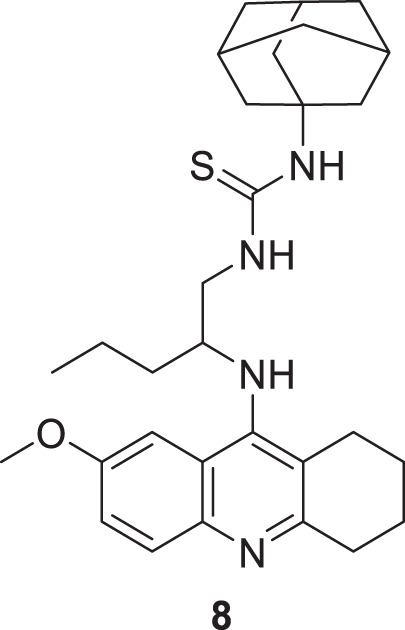


Spilovska et al. developed 7-methoxy derivatives, 9-amino-7-methoxy-1,2,3,4-tetrahydro acridine (7-MEOTA) linked with urea and thiourea and amantadine, found less hepatotoxic compared to tacrine. 1-(Adamantan-1-yl)-3-(2-((7-methoxy-1,2,3,4-tetrahydroacridin-9-yl) amino)pentyl)thiourea (**8**) linked with thiourea from this series showed potent *hAChE* (IC_50_ = 0.47 ± 0.09 µM) and *BuChE* (IC_50_ = 0.11 ± 0.02 µM) inhibitory activity (Spilovska et al., 2015).


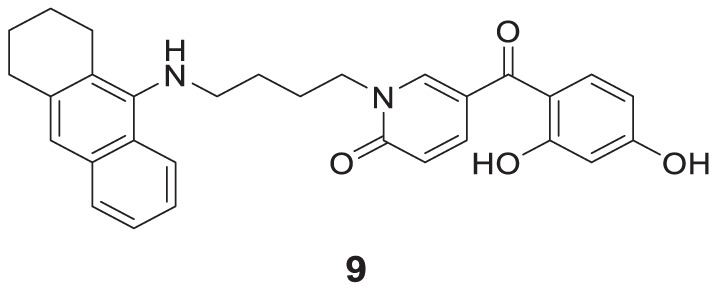


Chand et al. designed tacrine conjugates with hydroxyl benzyl-pyridone (TAC-HBP), showing dual binding at both the catalytic active site (CAS) and peripheral anionic site (PAS) of cholinesterase. 5-(2,4-Dihydroxybenzoyl)-1-(4-((1,2,3,4-tetrahydroacridin-9-yl)amino)butyl) pyridin-2(1*H*)-one (**9**) showed promising inhibitory activity against *AChE* (IC_50_ = 0.521 ± 0.025 µM). Also, this compound had radical scavenging activity comparable with tacrine (Chand et al., 2016).


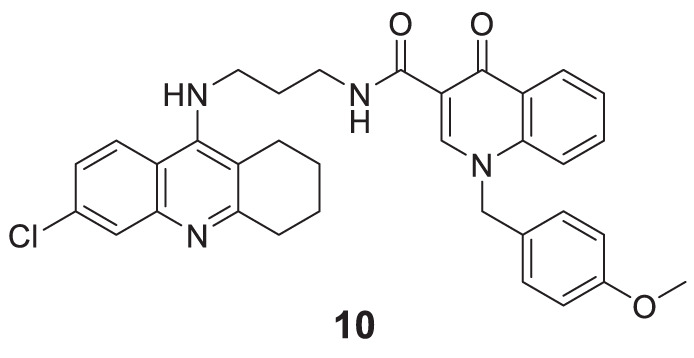


Hepnarova et al. designed novel tacrine-benzyl quinolone carboxylic acid (tacrine-BQCA) hybrids with the rationale that tacrine moiety would be responsible for *AChE* inhibition and BQCA have M1 receptor antagonist properties. *N*-(3-((6-chloro-1,2,3,4-tetrahydroacridin-9-yl)amino)propyl)-1-(4-methoxybenzyl)-4-oxo-1,4-dihydroquinoline-3-carboxamide **10** indicated a non-selective cholinesterase inhibition profile (IC_50_ = 0.0745 ± 0.0031 μM) with an affinity towards the M1 receptor and moderate brain permeability (Hepnarova et al., 2018).


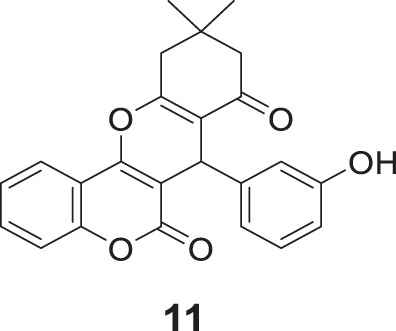


Ebrahimi et al. designed a new series of hetero-annulated chromene-fused coumarins against cholinesterase. 7-(3-Hydroxyphenyl)-10,10-dimethyl-7,9,10,11-tetrahydro-6*H*,8*H*-chromeno [4,3-*b*]chromene-6,8-dione (**11**) having 3-hydroxyphenyl moiety showed the highest inhibitory activity against *AChE* (IC_50_ = 3.28 µM) and *BuChE* (IC_50_ = 2.19 µM).

Researchers modulated selectivity for *AChE* and *BuChE* by introducing substitution at the 3-hydroxyphenyl group. The docking studies with *AChE* enzyme complexed with donepezil revealed that the coumarin ring was involved in π-π stacking with Trp279 and hydrogen bond with the hydroxyl group, that enabled tight binding with a receptor (Ebrahimi et al., 2016).


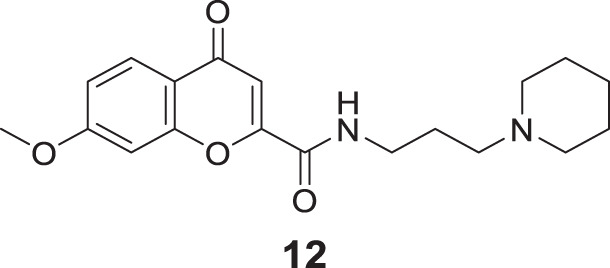


Suwanhom et al. similarly studied carboxamide derivatives and designed chromon-2-carboxamido alkylamines. 7-Methoxy-4-oxo-*N*-(3-(piperidin-1-yl)propyl)-4*H*-chromene-2-carboxamide (**12**) displayed a potent *AChE* inhibitory activity (IC_50_ = 0.09 ± 0.02 μM) than tacrine (IC_50_ = 0.13 ± 0.02 μM). The enzyme kinetics revealed that compound **12** is an uncompetitive inhibitor, and the docking study speculated the compound as a dual-binding inhibitor. Also, the cytotoxic effect was less, and the neuroprotective effect was more (Suwanhom et al., 2020).


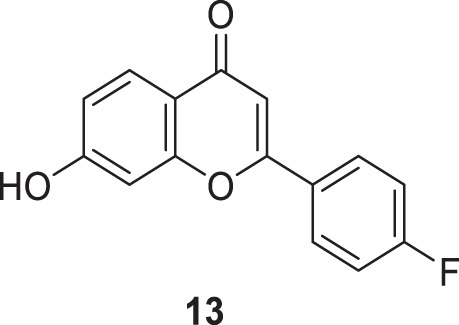


Singh et al. designed a novel class of 2-phenyl-4*H*-chromene-4-one derivatives as *AChE* inhibitors. 2-(4-Fluorophenyl)-7-hydroxy-4*H*-chromen-4-one (**13**) showed higher inhibitory activity (IC_50_ = 8.0 ± 0.37 µM) than donepezil (IC_50_ = 12.7 nM). The derivatives also exhibited the ability to inhibit advanced glycation end products with additional radical scavenging activity. Docking of compound **13** revealed good binding affinity at *CAS* and *PAS* of the enzyme active site (Singh M. et al., 2018).


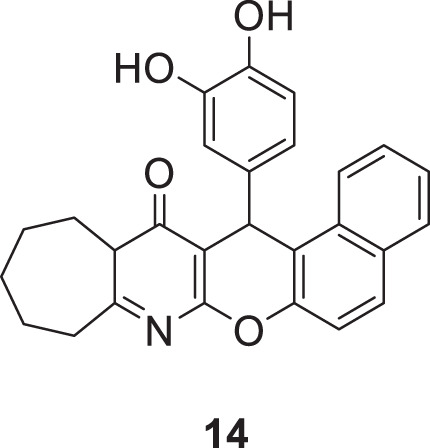


Macha et al. designed and synthesized tetrahydro-9*H*-benzo [5,6]chromeno [2,6-*b*]quinoline-13(14*H*)-one derivative against *AChE* and *BuChE*. The most potent inhibitory activity was shown by 15-(3,4-dihydroxyphenyl)-10,11,12,13,13a,15-hexahydrobenzo [5,6]chromeno [2,3-*b*]cyclohepta [*e*]pyridin-14(9*H*)-one (**14**) bearing 3,4-dihydroxy phenyl group at 15th position of hexahydrobenzo [5,6]chromeno [2,3-*b*]cyclohepta [*e*]pyridine-14(15*H*)-one scaffold against *AChE* (IC_50_ = 0.65 ± 0.06 μM) and *BuChE* (IC_50_ = 1.32 ± 0.06 μM). Compound **14** was safe with no hepatotoxicity and was equally active in behavioral studies Y maze, rectangle maze, and jumping box test compared to tacrine. A docking study revealed that the designed compounds bind well within the enzyme’s active site (Machaa et al., 2021).


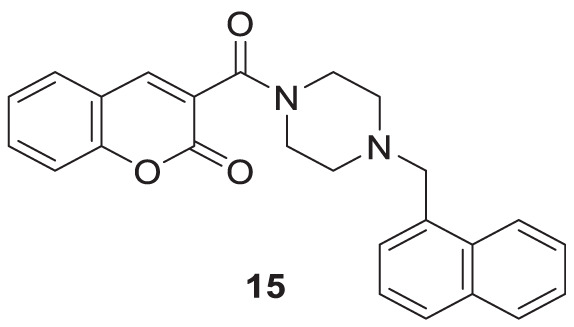


Zhang et al. developed a series of novel cholinesterase inhibitors by hybridizing coumarin and piperazine pharmacophores. 3-(4-(naphthalen-1-ylmethyl)piperazine-1-carbonyl)-2*H*-chromen-2-one (**15**) was identified as a potent inhibitor against *hAChE* (IC_50_ = 8.78 ± 0.22 μM), docking study revealed that hybrids target both *CAS* and *PAS* of *hAChE*, also showed no cytotoxicity against neuroblastoma cells (Zhang and Jiang, 2018).


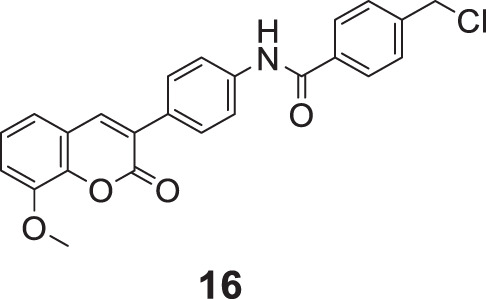


Hu et al. designed 3–(4-aminophenyl)-coumarins for AD treatment, 27 compounds were evaluated for anti-Alzheimer’s activity, and a behavioral inhibition study was performed on a model of zebrafish juveniles. 4-(Chloromethyl)-*N*-(4-(8-methoxy-2-oxo-2*H*-chromen-3-yl)phenyl)benzamide (**16**) exhibited the highest activity against *AChE* (IC_50_ = 0.091 ± 0.011 μM) but was slightly weaker than donepezil (IC_50_ = 0.012 ± 0.001 μM), and *BuChE* inhibitory activity (IC_50_ = 0.559 ± 0.017 μM) was more significant than donepezil (IC_50_ = 2.665 ± 0.015 μM) (Hu et al., 2019).


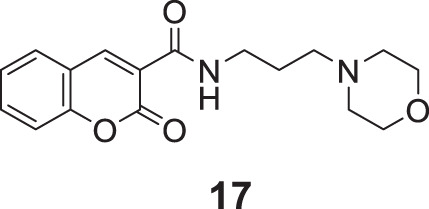


Tehrani et al. developed coumarin-3-carboxamide-*N*-morpholine hybrids as cholinesterase inhibitors. Among these compounds, propyl morpholine derivative *N*-(3-morpholinopropyl)-2-oxo-2*H*-chromene-3-carboxamide (**17**) with unsubstituted coumarin moiety depicted the highest *AChE* inhibitory activity (IC_50_ = 6.21 ± 0.03 μM). Docking and kinetic studies revealed the dual binding ability of compound **17** (Tehrani et al., 2019).


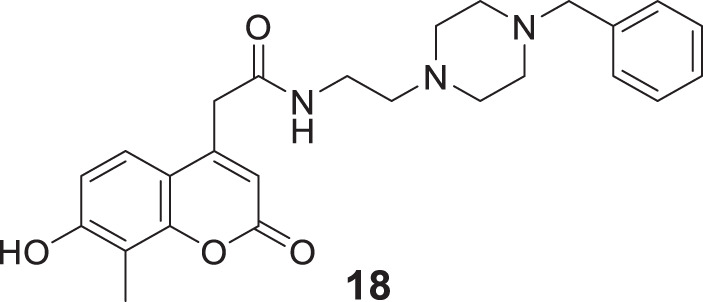


Kara et al. reported coumarin-based compounds as novel inhibitors of cholinesterase, and 2-(2-oxo-2H-chromen-4-yl) acetamide were identified as potent *AChE* inhibitors. Among the compounds in the series, *N*-(2-(4-benzylpiperazin-1-yl)ethyl)-2-(7-hydroxy-8-methyl-2-oxo-2*H*-chromen-4-yl)acetamide (**18**) depicted a good pharmacokinetic profile and high activity against both *huAChE* (IC_50_ = 0.04 ± 0.01 μM) and *BuChE* (IC_50_ = 0.68 ± 0.07 μM) than donepezil *huAChE* (IC_50_ = 0.004 ± 0.0001 μM) and *BuChE* (IC_50_ = 1.90 ± 0.02 μM). The docking study showed good interaction with the enzyme’s active site, and no hepatotoxicity was observed (Kara et al., 2019).


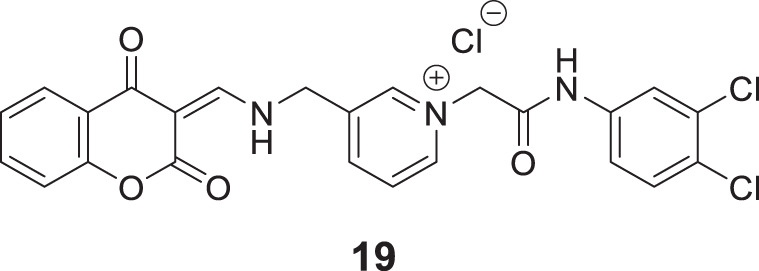


In another captivating research, Mollazadeh et al. synthesized 2,4-dioxochroman-*N*-phenyl pyridinium acetamide and evaluated for its *AChE* and *BuChE* inhibitory activities. (*Z*)-1-(2-((3,4-dichlorophenyl)amino)-2-oxoethyl)-3-((((2,4-dioxochroman-3-ylidene)methyl)amino) methyl)pyridin-1-ium chloride (**19)** was identified as a potent inhibitor for *BuChE* (IC_50_ = 3.66 ± 0.11 μM) and also showed good inhibition against *AChE* (IC_50_ = 10.30 ± 1.05 μM) compared with the standard drug donepezil. Docking and molecular dynamic studies confirmed that compound **19** interacted with the critical residues of the enzyme’s active site. Also, the *in-vitro* and *in silico* toxicity assays demonstrated the active compound to be non-toxic (Mollazadeh et al., 2020).


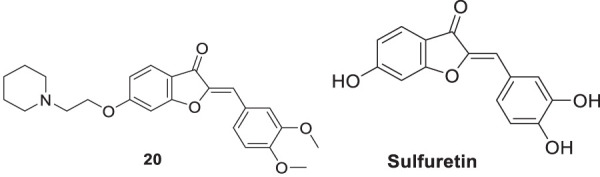


Lee et al. synthesized alkyl-substituted aurone derivative (*Z*)-2-(3,4-dimethoxybenzylidene)-6-(2-(piperidin-1-yl)ethoxy)benzofuran-3(2*H*)-one (**20**) using sulfuretin as a hit molecule. Researchers compared its potency with sulfuretin and galantamine, which have *AChE* inhibitory activity. Compound **20** displayed good inhibitory activity against *AChE* (IC_50_ = 0.40 ± 0.03 µM), was ca. 1700-fold higher than sulfuretin (IC_50_ = 698.9 μM) and ca. 6-fold higher than galantamine (IC_50_ = 2.50 μM) (Lee et al., 2015).


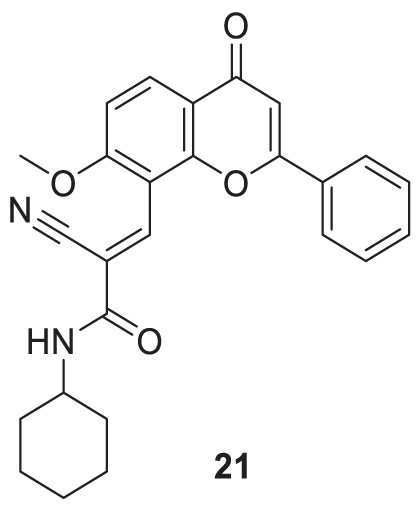


Shaikh et al. synthesized seventeen flavones-8-acrylamides and evaluated them for anti-Alzheimer activity. (*E*)-2-Cyano-*N-*cyclohexyl-3-(7-methoxy-4-oxo-2-phenyl-4*H*-chromen-8-yl)acrylamide (**21**) demonstrated higher selectivity (IC_50_ = 0.064 ± 0.004 µM) and inhibition than the approved cholinesterase inhibitors; galantamine and tacrine. Further, kinetic and molecular docking studies indicated that these molecules exhibit mixed inhibition. Compound **21** significantly reduces Aβ-induced toxicity. It contains the *N*-cyclohexyl group at the amido functional group, offering a neuroprotective effect and less toxicity to human neuroblastoma cells in all concentrations. This series of compounds also displayed anti-oxidant activity (Shaik et al., 2019).


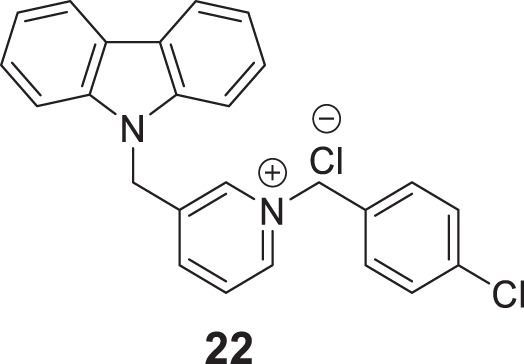


Ghobadian et al. reported *N*-benzyl-3-carbazolylpyridines as *BuChE* inhibitors. Compound **22** (3-((9*H*-carbazol-9-yl)methyl)-1-(4-chlorobenzyl)pyridin-1-ium chloride) (IC_50_ = 0.073 ± 0.003 µM) was identified as a potent and selective inhibitor of *BuChE*. A molecular docking study revealed a strong interaction of this compound with CAS and PAS of the enzyme with favorable physicochemical properties as a CNS drug. In addition, compound **22** demonstrated inhibition of self-induced Aβ peptide aggregation and neuroprotective activity (Ghobadian et al., 2018).


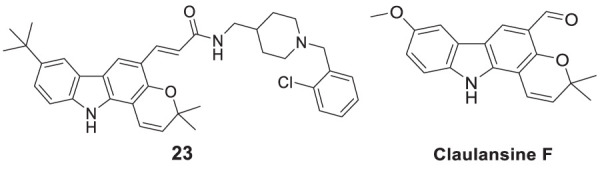


In another project by Zang et al., co-workers developed a series of Claulansine F-donepezil hybrids as multitarget drugs. Among 26 compounds studied, six compounds showed excellent *AChE* inhibitory activity. (*E*)-3-(8-(*t*-butyl)-3,3-dimethyl-3,11-dihydropyrano [3,2-*a*]carbazol-5-yl)-*N*-((1-(2-chlorobenzyl)piperidin-4-yl)methyl)acrylamide (**23**) was the most potent (IC_50_ = 4.34 ± 0.46 μM) and displayed the strongest *in-vitro* neuroprotective activity. Most importantly, **23** was able to cross BBB and also demonstrated radical scavenging activity *in-vitro*, proving to be a potential candidate for the treatment of AD (Zang et al., 2021).


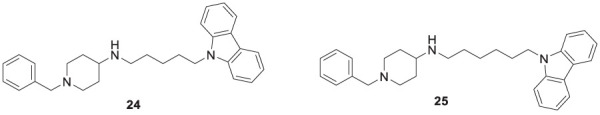


Sadeghiana et al. reported a novel series of anti-Alzheimer’s agents. In this study, they designed, synthesized, and evaluated a carbazole-benzyl piperidine hybrid for cholinesterase inhibition. *N*-(5-(9*H*-carbazol-9-yl)pentyl)-1-benzylpiperidin-4-amine (**24**) and *N*-(6-(9*H*-carbazol-9-yl)hexyl)-1-benzylpiperidin-4-amine (**25**) from this series indicated potent anticholinesterase activity. Compound **24** showed IC_50_ of 16.5 μM for *AChE* and IC_50_ of 0.59 μM for *BuChE*) and **25** showed IC_50_ of 26.5 μM for *AChE* and IC_50_ of 0.18 μM for *BuChE*. These two compounds also displayed *β-secretase* inhibition. Here, benzyl piperidine is linked with carbazole via a carbon chain linker. This linker with five (**24**) and six (**25**) carbon atoms exhibited good activity (Sadeghian et al., 2020).


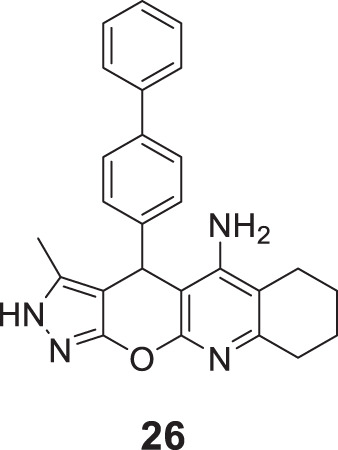


Derabli et al. developed new tacrine analogues modified with pyrano-pyrazole. Among the analogues, 4-([1,1′-biphenyl]-4-yl)-3-methyl-2,4,6,7,8,9-hexahydropyrazolo [4′,3':5,6]pyrano [2,3-*b*]quinolin-5-amine (**26**) displayed strong *AChE* inhibition (IC_50_ = 0.044 ± 0.002 μM) compared to the reference drug galantamine (IC_50_ = 21.82 ± 4.00 μM) (Derabli et al., 2018).


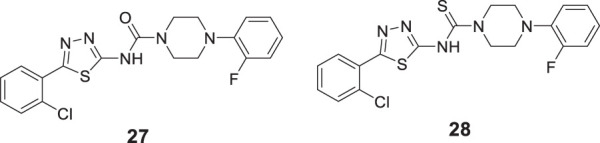


Kulshreshtha et al. studied novel urea and thiourea derivatives as cholinesterase inhibitors. Among them, *N*-(5-(2-chlorophenyl)-1,3,4-thiadiazol-2-yl)-4-(2-fluorophenyl)piperazine-1-carboxamide (**27**) (IC_50_ = 3.78 ± 0.63 μM) and *N*-(5-(2-chlorophenyl)-1,3,4-thiadiazol-2-yl)-4-(2-fluorophenyl)piperazine-1-carbothioamide (**28**) (IC_50_ = 1.51 ± 0.25 μM) showed promising activity against *AChE*. *In-vivo*, behavioral studies on scopolamine-induced animal models indicated that thiourea compound **28** was more potent than urea derivative **27** in alleviating cognition decline (Kulshreshtha and Piplani, 2018).


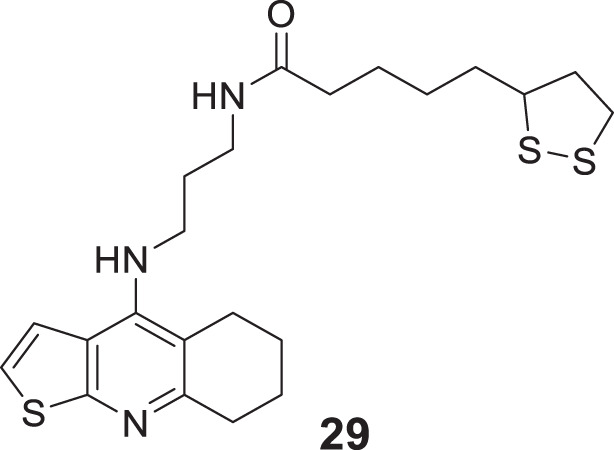


Pyridine is among the most studied heterocycles in drug design for various diseases, and also it is explored as anti-Alzheimer agent. Badran et al. applied a modern drug design strategy and developed tacrine heterodimer analogues thienopyridines, replacing the benzene ring of tacrine with bio isostere thiophene. 5-(1,2-Dithiolan-3-yl)-*N*-(3-((5,6,7,8-tetrahydrothieno [2,3-*b*]quinolin-4-yl)amino)propyl)pentanamide (**29**) with lipoic acid moiety exhibited higher *AChE* inhibitory activity (inhibition = 56.73%) than tacrine (inhibition = 54.91%). Lipoic acid moiety possesses antioxidant properties and dual binding affinity to the cholinesterase enzyme (Badran et al., 2013).


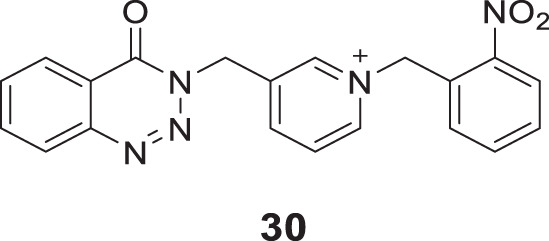


In a separate study, Hosseini et al. introduced substitution at the nitrogen atom of the pyridine ring and designed pyridinium derivatives replacing benzyl piperidine moiety of donepezil to obtain the lead compound and further modified to develop 4-oxobenzo [*d*]1,2,3-triazin benzyl pyridinium derivatives. Among them, 1-(2-nitrobenzyl)-3-((4-oxobenzo [*d*][1,2,3]triazin-3(4*H*)-yl)methyl)pyridin-1-ium bromide (**30**) with 2-nitro substitution in the benzene ring, revealed higher *AChE* inhibitory activity (IC_50_ = 0.10 ± 0.01 μM) than donepezil. Docking studies revealed that compound **30** interacted with CAS, PAS, catalytic triad, and oxyanion hole of the *AChE* enzyme (Hosseini et al., 2020).


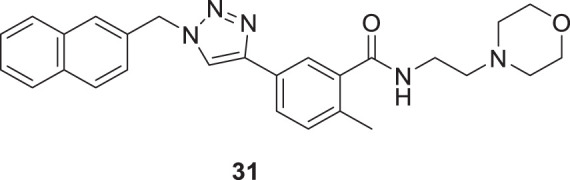


Li et al. synthesized novel triazole-based derivatives as cholinesterase inhibitors. 2-Methyl-*N*-(2-morpholinoethyl)-5-(1-(naphthalen-2-ylmethyl)-1*H*-1,2,3-triazol-4-yl)benzamide (**31**), having naphthalene substitution at the triazole ring, exhibited improved anti-cholinesterase activity and selectivity for *AChE* (IC_50_ = 7.23 ± 0.16 µM) than *BuChE* (IC_50_ = 90.76 ± 0.21 µM). SAR studies demonstrated that the benzene ring with both, electron-withdrawing and donating group substitutions reduced the potency and selectivity of the cholinesterase enzyme. Interestingly, the molecules have physicochemical properties similar to *CNS* drugs with less cytotoxicity, as observed in human *keratinocytes* HaCaT and murine *fibroblasts* NIH-3T3 cell lines. Also, the neuroprotective effect of the molecules was studied *in-vitro* in SH-SY5Y cells (Li J. C. et al., 2016).


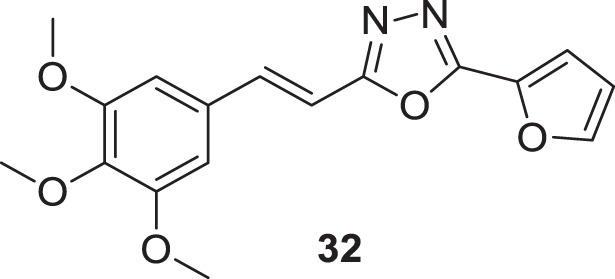


Kamal et al. designed and synthesized a library of (*E*)-2-aryl-5-styryl-1,3,4-oxadiazole derivatives following a molecular modeling strategy. The library demonstrated good to moderate activity against *AChE and* docking studies indicated binding of these derivatives was similar to donepezil. (*E*)-2-(furan-2-yl)-5-(3,4,5-trimethoxystyryl)-1,3,4-oxadiazole (**32**) exhibited higher activity (IC_50_ = 13.72 ± 0.01 µM) compared to other heterocyclic derivatives studied (Kamal et al., 2014).


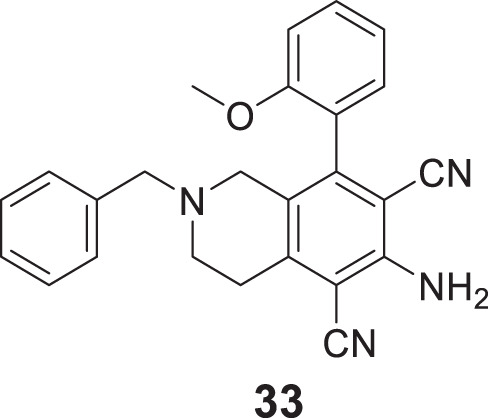


Sukumarapillai et al. developed *N*-benzyl piperidine-4-one derivatives as anti-Alzheimer’s agents. The compounds synthesized were mono-substituted and di-substituted, showing selective inhibition against *BuChE* and *AChE,* respectively. 6-amino-2-benzyl-8-(2-methoxyphenyl)-1,2,3,4-tetrahydroisoquinoline-5,7-dicarbonitrile (**33**) bearing ortho-methoxy group was potent with mixed-mode inhibitory activity against both *AChE* (IC_50_ = 5.61 ± 0.22 µM) and *BuChE* (IC_50_ = 0.87 ± 0.03 µM) compared to galantamine (AChE, IC_50_ = 2.09 ± 0.04 µM and *BuChE* IC_50_ = 19.34 ± 0.10 µM). Docking studies revealed that compound **33** displayed hydrophobic interaction at the catalytic triad and choline-binding sites (Sukumarapillai et al., 2016).


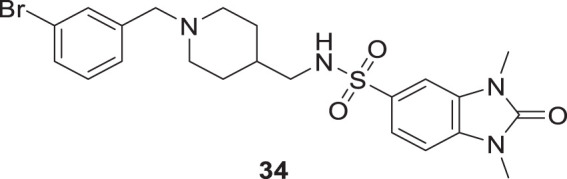


Mo et al. designed novel anti-Alzheimer’s agents linking benzyl piperidine with benzimidazolidinone ring, developing benzyl piperidine linked 1,3-dimethylbenz imidazolidinone derivatives. *In-vitro* cholinesterase inhibition assay demonstrated that the derivatives were good cholinesterase inhibitors. Among the derivatives, *N*-((1-(3-bromobenzyl)piperidin-4-yl)methyl)-1,3-dimethyl-2-oxo-2,3-dihydro-1*H*-benzo [*d*]imidazole -5-sulfonamide (**34**) was identified as a potent inhibitor of both *AChE* (IC_50_ = 0.39 ± 0.15 μM) and *BuChE* (IC_50_ = 0.16 ± 0.04 μM) in the sub-micromolar range. Cytotoxicity studies revealed that compound **34** is less hepatotoxic than donepezil. Considerable amelioration of cognitive impairment was observed in scopolamine-treated mice in the Morris maze test, and it also exhibited cytoprotective and antioxidant activity (Mo et al., 2020).


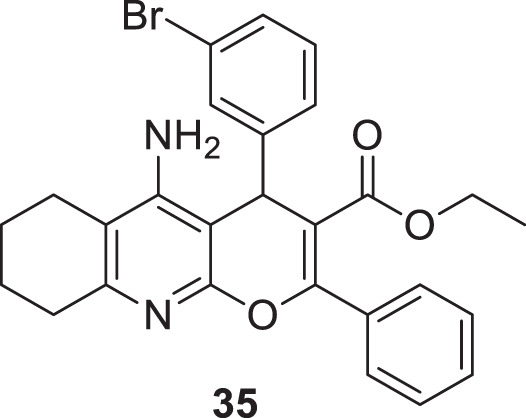


Eghtedari et al. developed tacrine-derived compounds as cholinesterase inhibitors, 5-amino-2-phenyl-4*H*-pyrano [2,3-*b*]-quinoline-3-carboxylates. Ethyl 5-amino-4-(3-bromophenyl)-2-phenyl-6,7,8,9-tetrahydro-4*H*-pyrano [2,3-*b*]quinoline-3-carboxylate (**35**) exhibited the most potent activity against *AChE* (IC_50_ = 0.069 ± 0.005 µM) and *BuChE* (IC_50_ = 1.35 ± 0.07 µM) that was five times more active than tacrine as evaluated by an *in-vitro* cholinesterase inhibition assay. The SAR by modifying substituents at the fourth position of pyrano moiety with substituted-phenyl ring revealed that electron-withdrawing groups such as *chloro* and *bromo* at *ortho* and *meta* positions improve the cholinesterase inhibitory potential of the compounds. The toxicity studies on HepG2 cells indicated that these compounds were less cytotoxic than tacrine (Eghtedari et al., 2017).


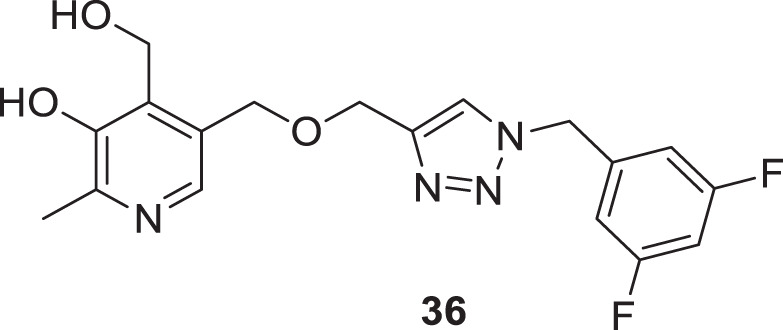


Pal et al. reported the synthesis and biological evaluation of a novel class of pyridoxine-based triazoles as cholinesterase enzyme inhibitors. Out of seventeen compounds, 5-(((1-(3,5-difluorobenzyl)-1*H*-1,2,3-triazol-4-yl)methoxy)methyl)-4-(hydroxymethyl)-2-methylpyridin-3-ol (**36**) showed higher *AChE* inhibitory activity (IC_50_ = 1.5609 ± 0.0237 mM). SAR studies revealed that *meta* and *ortho* substitutions on the aromatic ring with electron-donating groups were favorable for *AChE* inhibitory activity. The antioxidant property of compound **36** found with *ORAC-FL* value was equivalent to trolox, and *in silico* studies revealed that compound **36** has suitable pharmacokinetic and drug-like properties (Pal et al., 2020).


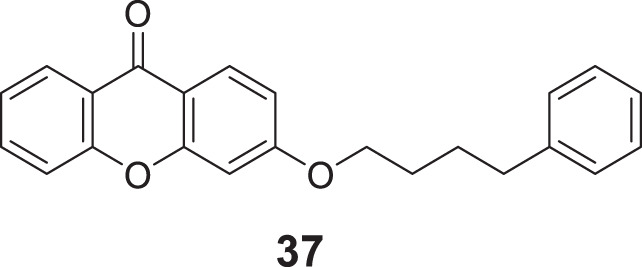


In a separate research, Loh et al. synthesized novel 3-O-substituted xanthone derivatives possessing more robust anti-cholinesterase activity. Eleven derivatives were identified as potent *AChE* inhibitors, and 3-(4-phenylbutoxy)-9*H*-xanthen-9-one (**37**) was the most potent among them with IC_50_ of 0.88 ± 0.04 μM. SAR study depicted hydrophobic interactions and hydrogen bonding of the substituents group, particularly saturated linear hydrocarbon chain having four carbons with the addition of phenyl or oxygenated groups are required to elicit activity. Docking studies revealed that the hydrophobic interaction is due to π-π stacking and hydrogen bonding contributed by the xanthone skeleton (Loh et al., 2021).


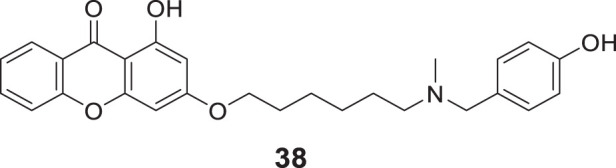


In another study, Zhang et al. developed novel xanthone-alkyl benzylamine hybrids with an alkyl linker and 1-hydroxy-3-((6-((4-hydroxybenzyl) (methyl)amino)hexyl)oxy)-9*H*-xanthen-9-one (**38**) was identified as the most potent *AChE* inhibitor (IC_50_ = 0.85 ± 0.043 μM) with balanced dual cholinesterase inhibition. Kinetic analysis and docking studies indicated compound **38** was a mixed type of inhibitor for *AChE* and *BuChE* with good blood-brain barrier (BBB) penetrability and antioxidant properties equivalent to trolox. Additionally, memory function improvement was observed in scopolamine-induced amnesia mice (Zhang Z. et al., 2021).


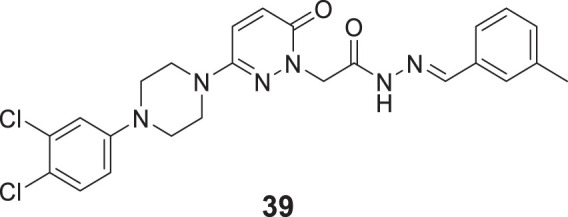


Ozdemir et al. reported the design and synthesis of 6-substituted-3(2*H*)-pyridazine-2-acetyl-2-(*p*-substituted benzalhydrazone) derivatives as potent dual cholinesterase inhibitors. (*E*)-2-(3-(4-(3,4-dichlorophenyl)piperazin-1-yl)-6-oxopyridazin-1(6*H*)-yl)-*N'*-(3-methylbenzylidene) acetohydrazide (**39**) was the most potent compound among the synthesized derivatives for *AChE* inhibition (IC_50_ = 75.52 ± 1.76%) and *BuChE* (IC_50_ = 62.03 ± 1.82%). Docking studies demonstrated that its binding interactions in *AChE* active sites were similar to those of the known inhibitors. However, it did not show binding abilities to the active site of *BuChE* (Özdemir et al., 2017).


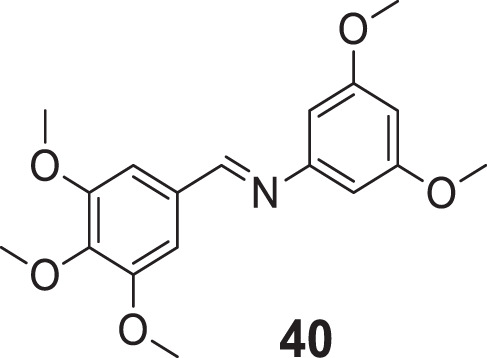


Shrivastava et al. designed and synthesized an *N*-methylene benzenamine nucleus containing 3,5-dimethoxy-*N*-methylene benzenamine and 4-(methylene amino)benzoic acid derivatives. (*E*)-*N*-(3,5-dimethoxyphenyl)-1-(3,4,5-trimethoxyphenyl)methanimine (**40**) depicted higher *AChE* inhibition (IC_50_ = 0.82 ± 0.05 µM) than donepezil evaluated. *Ex-vivo* studies confirmed the ability of compound **40** to cross the BBB and selective inhibition of *AChE*. In addition, compound **40** also exhibited good *in-vitro* radical scavenging ability (Shrivastava et al., 2017).


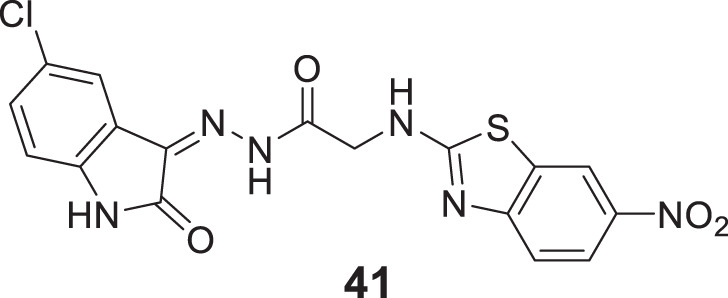


Tripathi et al. developed 2-amino-6-nitrobenzothiazole-derived hydrazones as *AChE* inhibitors. (*Z*)-*N'*-(5-chloro-2-oxoindolin-3-ylidene)-2-((6-nitrobenzo [*d*]thiazol-2-yl)amino) acetohydrazide (**41**) indicated six-fold potency against *AChE* (IC_50_ = 0.0035 ± 0.005 µM) than donepezil and tacrine. This compound demonstrated mixed-type reversible enzyme inhibition with a good docking score. Further, the radical scavenging ability of the active molecules was higher as confirmed by α,α-diphenyl-β-picrylhydrazyl (*DPPH)* radical scavenging assay (Tripathi and Ayyannan, 2018).


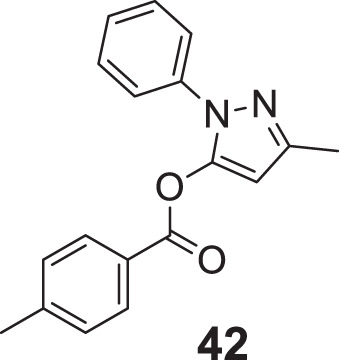


In another captivating research, Carrillo et al. synthesized and evaluated aliphatic and aromatic edaravone derivatives as antioxidant and *AChE* inhibitors by *DPPH* and *in-vitro AChE* inhibition assay, respectively. Aliphatic derivatives were not as active as the standard drug galantamine, but aromatic derivatives exhibited better general activity; among them, 3-methyl-1-phenyl-1*H*-pyrazol-5-yl-4-methylbenzoate (**42**) showed the highest percent inhibition (inhibition = 41.9 ± 7.3%). Fascinatingly, all synthesized compounds showed drug-like properties capable of crossing BBB, and docking analysis revealed compounds have good interaction at the *AChE* catalytic gorge site (Barajas-Carrillo et al., 2021).


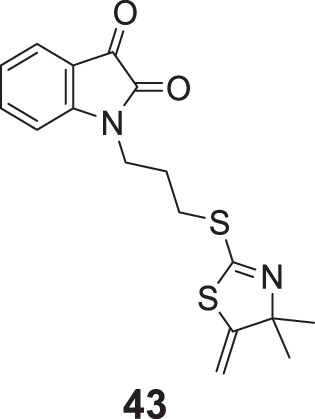


Davis et al. designed a novel series of isatin-linked 4,4-dimethyl-5-methylene-4,4-dihydro thiazole-2-thiols as *AChE* inhibitors. 1-(3-((4,4-Dimethyl-5-methylene-4,5-dihydrothiazol-2-yl)thio)propyl)indoline-2,3-dione (**43**) was the most potent inhibitor (IC_50_ = 18.2 ± 1.2 μM) and the potency was similar to galantamine. Kinetic studies indicated that compound **43** was a non-competitive reversible inhibitor, whereas molecular modeling indicated interaction with *AChE* active site. Isatin moiety showed interaction with CAS and 2-thiazoline moiety with PAS of *AChE* (Davis and Eckroat, 2021).


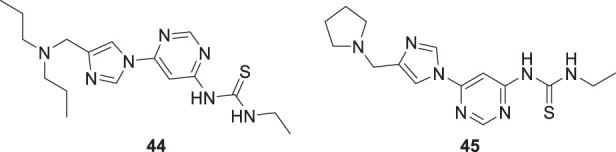


Xiaokang et al. designed, synthesized, and evaluated non-fused pyrimidinyl thiourea derivatives by screening hit compounds and modifications. These are multifunctional agents. 1-(6-(4-((dipropylamino)methyl)-1*H*-imidazol-1-yl)pyrimidin-4-yl)-3-ethyl thiourea (**44**) and 1-ethyl-3-(6-(4-(pyrrolidin-1-ylmethyl)-1*H*-imidazol-1-yl)pyrimidin-4-yl) thiourea (**45**) show good inhibition and binding selectivity for *AChE*. Compound **44** (IC_50_ = 0.204 μM) and compound **45** (IC_50_ = 0.067 μM) have been found to exhibit notable *AChE* inhibition. These compounds demonstrated multiple activities such as specific metal-chelating ability, anti-oxidant effects, and modulation of metal-induced Aβ aggregation (Li X. et al., 2016).


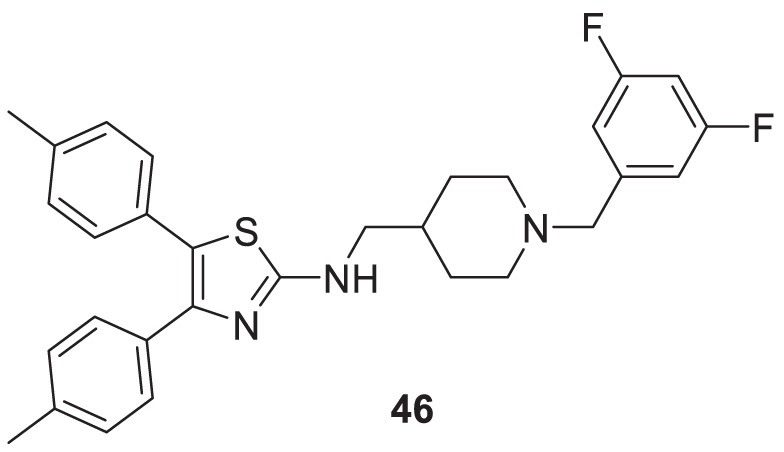


Shidore et al. synthesized novel molecules by fusing cholinesterase inhibitor donepezil and diaryl thiazole. *N*-((1-(3,5-difluorobenzyl)piperidin-4-yl)methyl)-4,5-di-*p*-tolylthiazol-2-amine (**46**) of the series exhibited potent anti-cholinesterase activity with IC_50_ of 0.30 ± 0.01 µM against AChE, and IC_50_ = 1.84 ± 0.03 µM against BuChE. In addition, compound **46** also showed *in-vitro* anti-oxidant and anti-apoptotic properties (Shidore et al., 2016).


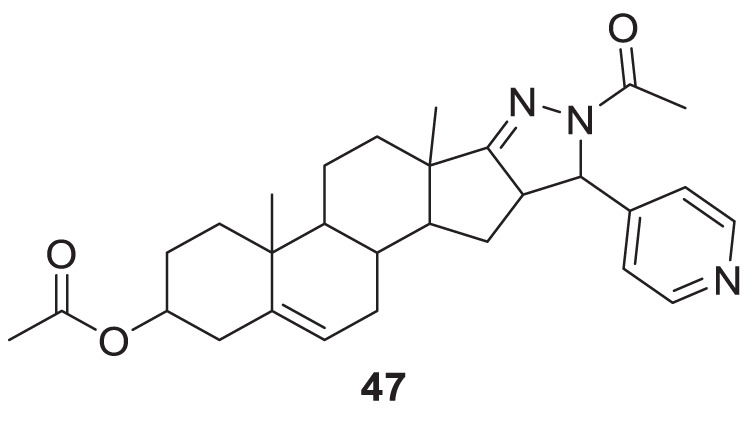


Singh et al. synthesized and evaluated pyrazolyl-substituted steroids as neuroprotective agents. Dehydroepiandrosterone is a steroid used to treat neurodegenerative disorders such as Alzheimer’s and Parkinson’s disease, which is substituted at the 16th and 17th positions with the pyrazolinyl group. 10-acetyl-6a,8a-dimethyl-11-(pyridin-4-yl)-1,3,4,5,6,6a,6b,7,8,8a,10,11, 11a,12,12a,12b-hexadecahydronaphtho [2′,1':4,5]indeno [1,2-*c*]pyrazol-4-yl acetate (**47**) displays potent neuroprotection with inhibition of *AChE* with micro moles of *AChE*/min/mg protein (0.0027 ± 0.0006) (Singh R. et al., 2018).


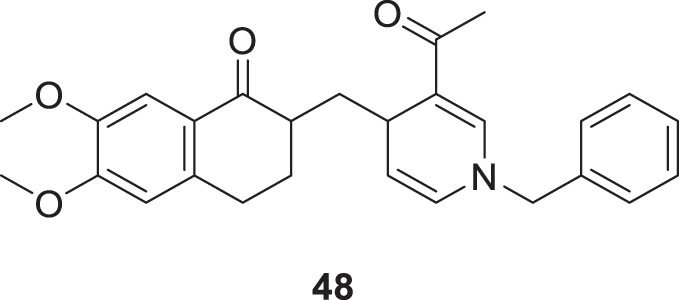


Tintas et al. synthesized and evaluated 1,4-dihydropyridine derivatives with significant *AChE* inhibition. 2-((3-Acetyl-1-benzyl-1,4-dihydropyridin-4-yl)methyl)-6,7-dimethoxy-3,4-dihydronaphthalen-1(2*H*)-one (**48**) displayed a good AChE inhibitory activity (IC_50_ = 0.173 µM). These are chiral 1,4-dihydropyridine derivatives that exhibited selectivity for *AChE* (Ţînţaş et al., 2018).


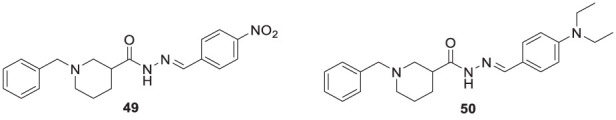


Parlar et al. designed, synthesized, and evaluated a series of *N*-benzylpiperidine-3/4-carbohydrazone derivatives for *AChE* inhibition. These SAR studies showed that the phenyl-substituted compound displayed selectivity for *AChE* binding. (*E*)-1-benzyl-*N*′-(4-nitrobenzylidene)piperidine-3-carbohydrazide (**49**) and (*E*)-1-benzyl-*N*′-(4-(diethylamino) benzylidene)piperidine-3-carbohydrazide (**50**) demonstrated the most potent activity in the given series of derivatives. Compound **49** indicated better *in-vitro AChE* inhibition with IC_50_ = 5.68 μM, and compound **50** diethylamino derivative exhibited *AChE* inhibition with IC_50_ = 0.81 μM. It also showed Aβ42 self-aggregation inhibition and anti-oxidant properties (Parlar et al., 2019).


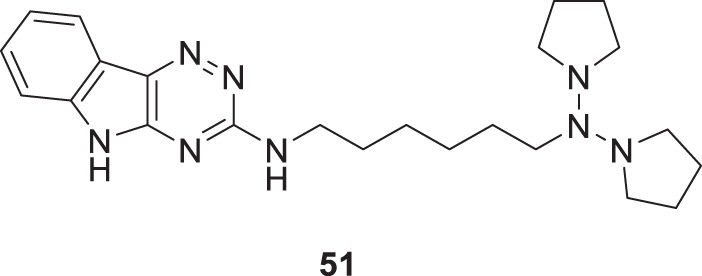


Patel et al. designed, synthesized novel triazinoindole derivatives and evaluated them for anti-cholinesterase activity. *N* ^1^-(5*H*-[1,2,4]triazino [5,6-*b*]indol-3-yl)-*N* ^6^ *,N* ^6^-di (pyrrolidin-1-yl)hexane-1,6-diamine (**51**) exhibited good cholinesterase inhibition from this series of derivatives. Compound **51** indicated IC_50_ = 0.56 ± 0.02 µM for *AChE* and IC_50_ = 1.17 ± 0.09 µM for *BuChE*. Pyrrolidine moiety in compounds shows better activity than compounds with other amines. These tertiary amines show pie-cation interaction with amino acids present in enzymes. A molecular docking study revealed that amino groups present in molecules interact with various amino acids in enzymes by hydrogen bonding. Compound **51** also exhibited improved anti-oxidant and neuroprotective properties than other molecules in this series (Patel et al., 2019).


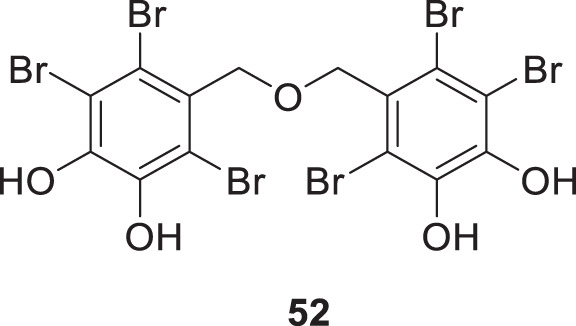


The marine ecosystem has a vast amount of different and unique bioactive secondary metabolites. Paudel et al. extracted bromophenols from red algae called Symphyocladia *latiuscula* Yamada. Further, they evaluated their biological activity against *AChE* and *BuChE*. Among all the extracted compounds, 5,5′-(oxybis (methylene))bis (3,4,6-tribromobenzene-1,2-diol) (**52**) indicated potent inhibition of the cholinesterase enzyme with a K_i_ value of 0.6 μM for *AChE* and 0.37 μM for *BuChE* inhibition. The OH groups in the structure exhibit hydrogen bond interaction with the enzyme revealed by the docking study. It also inhibits Aβ aggregation (Paudel et al., 2019).


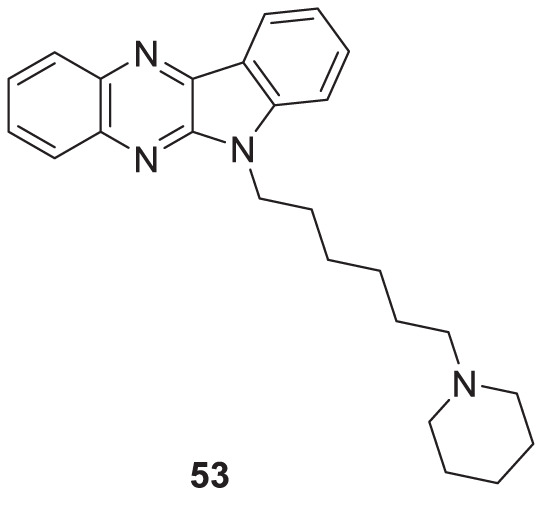


Kanhed et al. developed a series of indoloquinoxaline derivatives. These indoloquinoxaline derivatives produced multitargeted activities against Alzheimer’s disease, such as cholinesterase inhibition, self-induced Aβ aggregation inhibition, and antioxidant activity. 6-(6-(Piperidin-1-yl)hexyl)-6*H*-indolo [2,3-*b*]quinoxaline (**53**) exhibits the most potent and selective inhibition of *BuChE* with IC_50_ of 0.96 ± 0.31 µM and inhibition of *AChE* with IC_50_ of 5.80 ± 0.70 µM, also, 51.24% inhibition of self-induced Aβ aggregation. A docking study revealed that six carbon linkers between amino and indole nitrogen provide better stability to the enzyme ligand complex. At the same time, the pyrrolidine ring presents weak hydrogen bond interaction and the indolo [2,3*-b*]quinoxaline ring form π-π interaction with the active site of the cholinesterase enzyme (Kanhed et al., 2022).


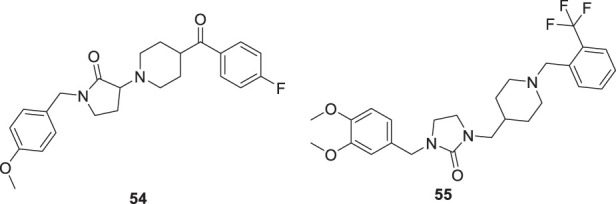


Donepezil is a primary drug used for the treatment of Alzheimer’s disease. Gupta et al. designed the analogues of donepezil based on the SAR of lead compound indanone moiety. The novel synthesized compounds indicated good *in-vivo* and *in-vitro* inhibition of cholinesterase. 3-(4-(4-Fluorobenzoyl)piperidin-1-yl)-1-(4-methoxybenzyl)pyrrolidin-2-one (**54**) and 1-(3,4-dimethoxybenzyl)-3-((1-(2-(trifluoromethyl)benzyl)piperidin-4-yl)methyl)imidazolidin-2-one (**55**) exhibited the most potent AChE inhibitory activity from this series with 0.018 ± 0.001 and 0.022 ± 0.002 µMol of *AChE*/min/mg of protein, respectively (Gupta et al., 2020).


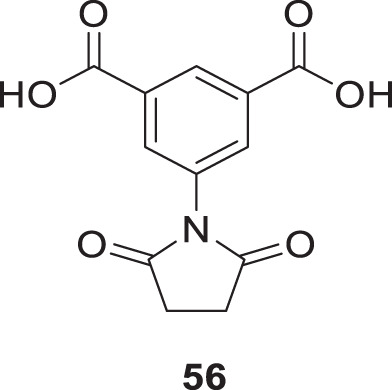


Altamirano-Espino et al. synthesized aminoisophthalic acid derivatives to inhibit acetylcholinesterase with fewer side effects. They have synthesized ten derivatives and evaluated them for *AChE* inhibition *in-vitro* and *in silico*. According to the docking simulation study, electron-poor aminobenzoic acid derivatives show better inhibition than electron-rich ones. However, when a heterocyclic ring is substituted in place of a linear group, it creates a π-π stacking interaction between the aromatic ring and an amino acid in the enzyme, resulting in a lower Ki value. The *in-vitro* assessment indicates that the derivative with a succinimide substitution compound, 5-(2,5-dioxopyrrolidin-1-yl)isophthalic acid (**56**), displays the lowest Ki value of 73 µM compared to the other compounds tested (Altamirano-Espino et al., 2020).


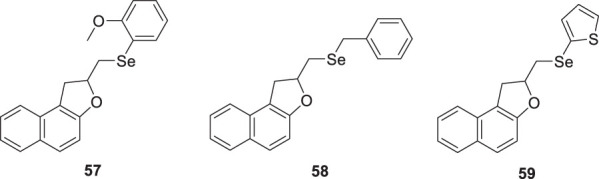


Scheide et al. synthesized allylnaphthol and allylphenol derivatives with diselenides via intramolecular electrochemical oxidation and cyclization. Further, they evaluated these compounds for anti-Alzheimer’s activity. From this series of compounds, three compounds revealed good inhibition of *AChE*. These are 2-(((2-methoxyphenyl)selanyl)methyl)-1,2-dihydronaphtho [2,1-*b*]furan (**57**) with IC_50_ = 10.6 µM, 2-((benzylselanyl)methyl)-1,2-dihydronaphtho [2,1-*b*]furan (**58**) with IC_50_ = 11.6 µM, 2-((thiophen-2-ylselanyl)methyl)-1,2-dihydronaphtho [2,1-*b*]furan (**59**) with IC_50_ = 9.97 µM (Scheide et al., 2020).


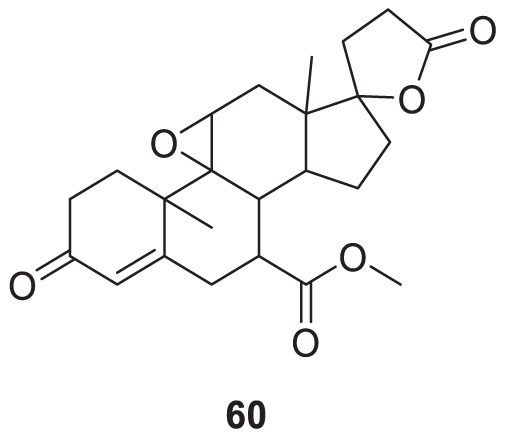


Hira et al. studied the potential aldosterone antagonist eplerenone, methyl 4a,6a-dimethyl-2,5′-dioxo-2,4,4a,4′,5a,5′,6,6a,8,9,9a,9b,10,11-tetradecahydro-3*H*,3′*H*-spiro [cyclopenta [1,2] phenanthro [4,4a-*b*]oxirene-7,2′-furan]-10-carboxylate, (**60**) for the treatment of Alzheimer’s disease. *In-vivo* and *in silico* studies showed that eplerenone is an effective drug for reversing STZ (streptozotocin)-induced memory impairment. It may be helpful in the treatment of Alzheimer’s disease and dementia (Hira et al., 2020).


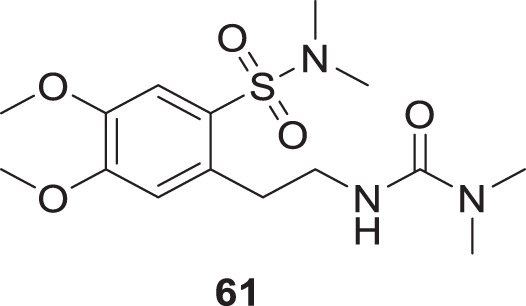


Gok et al. focused on dopamine as a treatment for AD. They have synthesized a series of dopamine analogues by introducing urea and sulfonamide groups into the dopamine moiety, resulting in novel active analogues with potent cholinesterase inhibition and anti-oxidant activity. 2-(2-(3,3-Dimethylureido)ethyl)-4,5-dimethoxy-*N,N*-dimethylbenzenesulfonamide (**61**) with *N,N*-dimethylsulfonamide and *N,N*-dimethylurea substitution exhibited inhibition of both *AChE* and *BuChE* with IC_50_ values of 298 µM and 321 μM, *respectively*. Docking studies revealed that this analog had good binding interaction with cholinesterase enzyme. Additionally, its ADME properties were in the acceptable range (Gök et al., 2021).


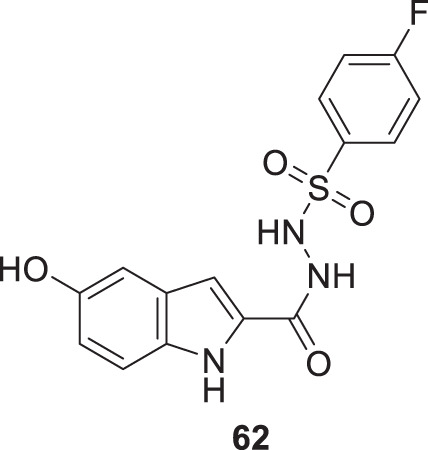


Taha et al. also synthesized the sulfonamide derivatives based on indole as a basic moiety. From this series, 4-fluoro-*N'*-(5-hydroxy-1*H*-indole-2-carbonyl)benzenesulfonohydrazide (**62**) displayed significant inhibition of *AChE* with an IC_50_ value 0.17 ± 0.02 µM. According to the docking study, the sulfonamide group interacts with the active site residues via hydrogen bonding, while the indole aromatic ring forms a π-π interaction. Additionally, the fluorine on the aromatic ring is observed to exhibit an interaction (Taha et al., 2021).


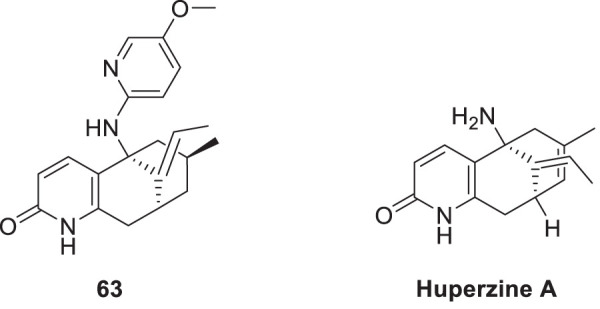


Huperzine A is a natural product having anti-cholinesterase activity, like galantamine. It has been obtained from Huperzia *serrata* as a sesquiterpene alkaloid. It is used in China as a standard therapy for dementia. Miao et al. modified huperzine and synthesized a new series of *N*-hetero (aryl) analogues of huperzine A. Further, they evaluated this analog for its anti-Alzheimer’s activity. From this synthesized series, (5*R*,7*S*,9*S*,*E*)-11-ethylidene-5-((5-methoxy pyridin-2-yl)amino)-7-methyl-5,6,7,8,9,10-hexahydro-5,9-methanocycloocta [*b*]pyridin-2(1*H*) -one (**63**) with 5-methoxy-2-pyridyl substitution displays potent *AChE* inhibition with IC_50_ value of 1.5 µM, which is 7.6 times more potent than huperzine A. It also indicated anti-oxidant activity. A molecular docking study speculates that compound **63** has good binding interaction. Aromatic moiety interacts via π-π stacking; methoxy pyridine shows hydrophobic interaction. This analog exhibited neuroprotective properties (Miao et al., 2021).


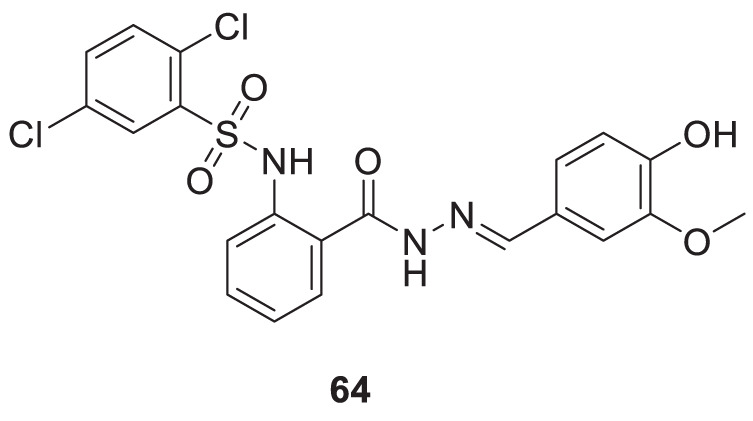


Murtaza et al. designed, synthesized, and evaluated a set of 2-aminobenzohydrazide and 2,3-dihydroquinazolin-4(*1H*)-one derivative as anti-cholinesterase agents. (*E*)-2,5-dichloro-*N*-(2-(2-(4-hydroxy-3-methoxybenzylidene)hydrazine-1-carbonyl)phenyl)benzene sulfonamide (**64**) shows dual inhibition of *AChE* and BuChE with IC_50_ values of 0.12 ± 0.03 µM and 0.13 ± 1.75 µM, respectively. A molecular docking study revealed that the three aromatic rings in **64** make π-π interaction with amino acids at the active site of cholinesterase. Relative to the preceding compound, heteroatoms exhibit distinct hydrogen bond interactions with amino acids. The molecule’s electronegative density is a crucial determinant of its binding capability. Additionally, this derivative displays a neuroprotective effect (Murtaza et al., 2022). See Supplementary Table 1 for a summary of cholinesterase inhibitors.

### 4.2 Aβ aggregates inhibitors

The amyloid hypothesis plays a vital role in the pathogenesis of Alzheimer’s disease. Hence the approach of blocking or slowing of Aβ aggregation attracted the attention of medicinal chemists.


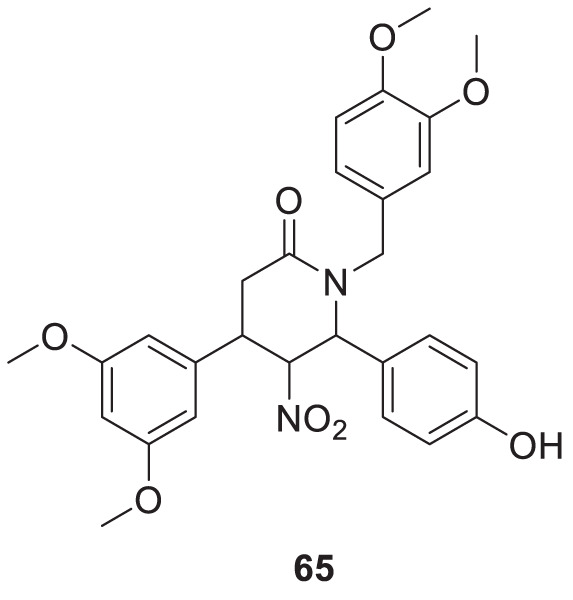


Li et al. developed a series of multipotent 2-piperidones against Alzheimer’s disease. 1-(3,4-Dimethoxybenzyl)-4-(3,5-dimethoxyphenyl)-6-(4-hydroxyphenyl)-5-nitro piperidin-2-one (**65**) exhibited the best concentration-dependant Aβ-self aggregation inhibition (59.11% at 20 µM) evaluated *in-vitro* by *thioflavin T* (ThT) *fluorescence assay*. Further, docking analysis revealed good binding to the active site of *myeloid differentiation factor* 88 (MyD88), preventing the dimerization of peptides. In addition, the synthesized compounds demonstrated less neurotoxicity and anti-inflammatory properties (Li L. et al., 2016).


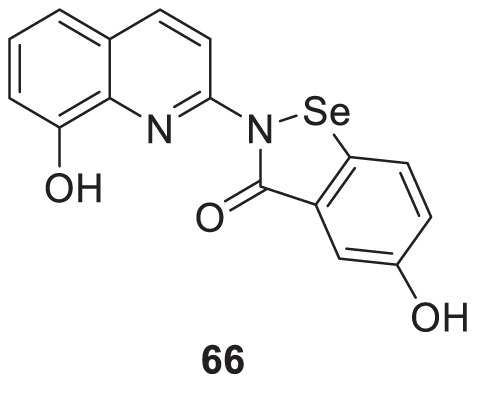


In another study, Wang et al. designed and synthesized 8-hydroxyquinolines bearing (benzo[*d*][1,2]selenazol-3(2*H*)-one) substitution at second position for the treatment of Alzheimer’s disease. Interestingly, 5-hydroxy-2-(8-hydroxyquinolin-2-yl)benzo [*d*][1,2] selenazol-3(2*H*)-one (**66**) demonstrated inhibition of Cu (II)-induced Aβ aggregation among the derivatives confirmed by ThT fluorescence assay. This compound also showed a good radical scavenging activity (2.6 ± 0.3 µM min^-1^) in *oxygen radical absorbance capacity* (ORAC FL) assay (Wang et al., 2016).


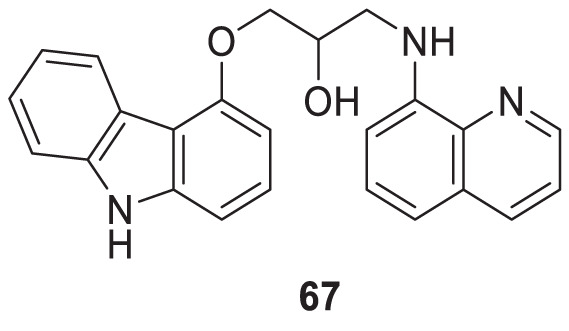


Interestingly, Zang et al. also explored a quinoline scaffold and developed a 4-hydroxy carbazole-8-aminoquinoline dimer, 1-((9*H*-carbazol-4-yl)oxy)-3-(quinolin-8-ylamino)propan-2-ol (**67**) as copper-induced Aβ aggregation inhibitor and reported 22.9% inhibition in ThT fluorescence assay. In addition, compound **67** exhibited good copper selective inhibition and neuroprotective effect against Glu-induced cell death in HT22 cells at 10 µM (Zhang et al., 2018).


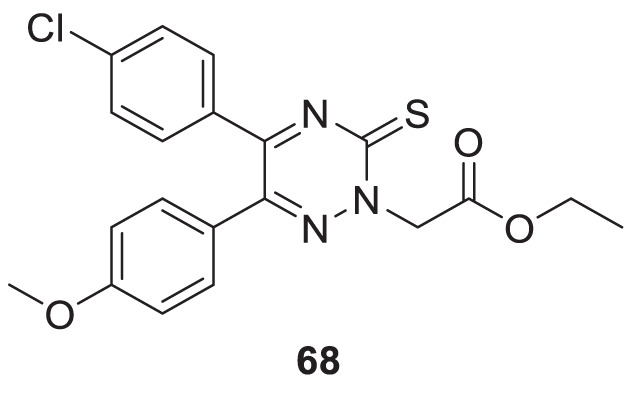


In a separate study by Kucukkilinc et al., co-workers designed, synthesized, and evaluated 5,6-diaryl-1,2,4-triazine-3-thioacetates against Aβ induced neurotoxicity and H_2_O_2_ toxicity. The neuroprotective activity of the compounds was evaluated on PC12 and SH-SY5Y cells; surprisingly, ethyl 2-(5-(4-chlorophenyl)-6-(4-methoxyphenyl)-3-thioxo-1,2,4-triazin-2(3*H*)-yl)acetate (**68**) was identified as the most potent derivative (EC_50_ = 14.44 ± 0.85 µM) and was less potent than quercetin (EC_50_ = 8.18 ± 1.45 µM). Cytometric analysis revealed the possibility of a 40% increase in cell viability in H_2_O_2_-induced apoptosis. Furthermore, *these* compounds also improved neuronal cells neurite outgrowth in *transferase-mediated dUTP nick end labelling* (TUNEL) assay (Tuylu Kucukkilinc et al., 2017).


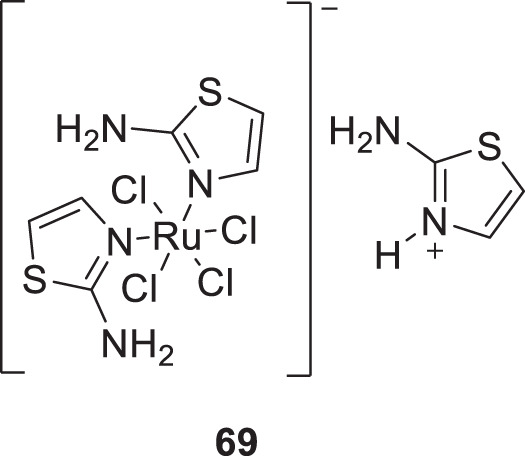


Plexes are an attractive class of drug discovery. Messori et al. studied ruthenium (III) complexes as an anti-Alzheimer’s agent having potent Aβ blocking properties. Among three metal complexes studied, (**69**) displayed potent activity against Aβ aggregation *in-vitro,* and this study confirmed PMRU20 at 20 μM as an effective neuroprotective agent (Messori et al., 2013).


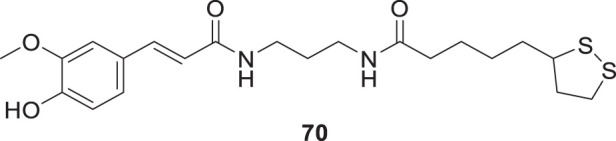


Pagoni et al. synthesized and evaluated 13 novel hybrids of phenols and lipoic acid as Aβ-aggregation inhibitors as well as antioxidant agents. From this series, (*E*)-5-(1,2-dithiolan-3-yl)-*N*-(3-(3-(4-hydroxy-3-methoxyphenyl)acrylamido)propyl)pentanamide (**70**) exhibited *in-vitro* activity against Aβ-aggregation. Additionally, it also displayed antioxidant properties with cytoprotective and non-cytotoxic action. Ferulic and dihydroxyphenylacetic acid derivatives (structures not shown) as phenolic groups indicate strong free radical scavenging and anti-amyloidogenic properties (Pagoni et al., 2020).


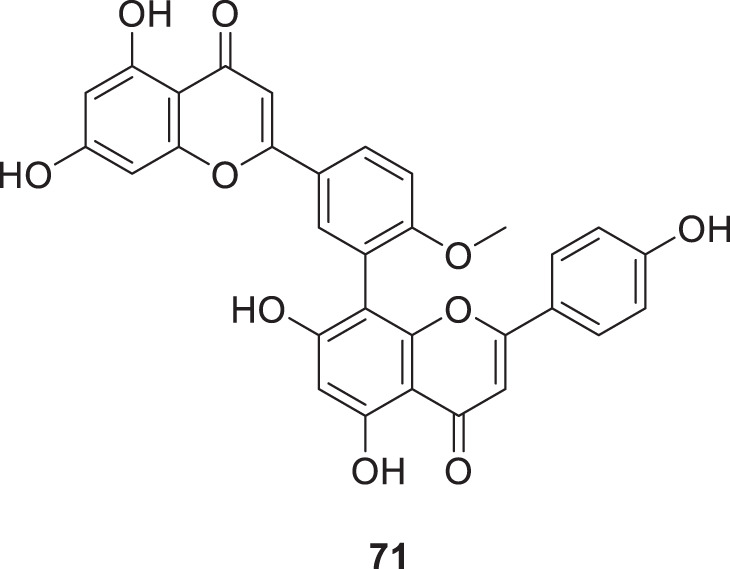


Sirimangkalakitti et al. reported the anti-Alzheimer’s activity of naturally occurring bioflavonoids as β aggregation inhibition. They have studied 27 bioflavonoids with different linkages and methoxy substitution. Among them, flavonoids amentoflavone and its methoxy derivatives show potent inhibition of Aβ aggregation *in vitro* and bilobetin, 8-(5-(5,7-dihydroxy-4-oxo-4*H*-chromen-2-yl)-2-methoxyphenyl)-5,7-dihydroxy-2-(4-hydroxyphenyl)-4*H*-chromen-4-one, (**71**) displayed an IC_50_ value of 4.7 µM (Sirimangkalakitti et al., 2019).


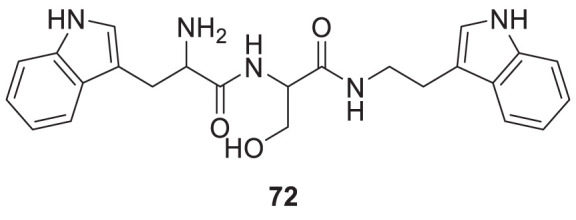


Wongrattanakamon et al. studied the anti-Aβ-aggregation activity of guanidinyl tryptophans. In this study, molecular dynamic simulation of Aβ monomer with various derivatives was conducted. Compound **72** indicated potent interference with Aβ monomer movement into the cell. *N*-(2-(1*H*-indol-3-yl)ethyl)-2-(2-amino-3-(1*H*-indol-3-yl)propanamido)-3-hydroxypropanamide (**72**) exhibited anti-amyloid aggregation with 49.8% ± 1.5% inhibition (Pathomwat et al., 2021).


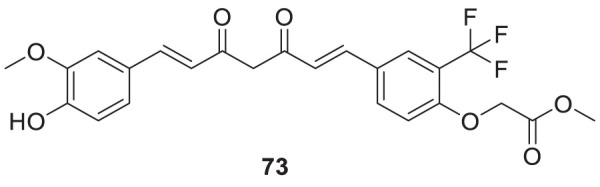


Utomo et al. developed curcumin-based Aβ aggregation inhibitors. Methyl 2-(4-((1*E*,6*E*)-7-(4-hydroxy-3-methoxyphenyl)-3,5-dioxohepta-1,6-dien-1-yl)-2-(trifluoromethyl)phenoxy) acetate (**73**) (IC_50_ = 0.007 µM) possessed 100-fold higher activity as Aβ aggregation inhibition than curcumin. Compounds with hydroxyl and methoxycarbonyl groups show more potent inhibition than curcumin. Compound **73** also showed lower cytotoxicity (Yudi Utomo et al., 2021). See Table 1 for a summary of Aβ aggregates inhibitors.

**TABLE 1:** Aβ aggregates inhibitors.

Sr.No.	Compound	Activity	Assay type
65	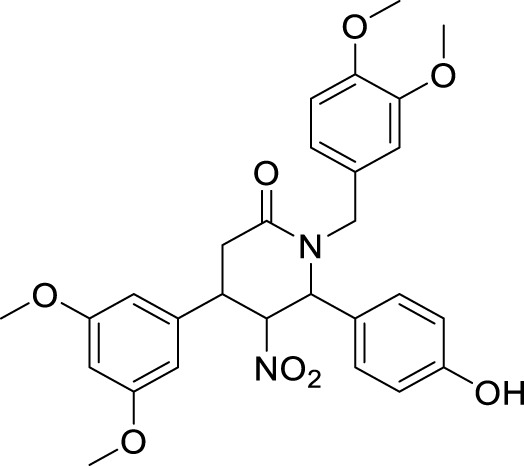	Aβ-self aggregation inhibition = 59.11%	ThT fluorescence assay
66	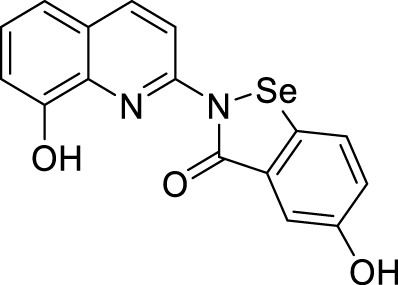	H_2_O_2_ radical scavenging activity = 2.6 ± 0.3 µM min^-1^	ORAC FL assay
67	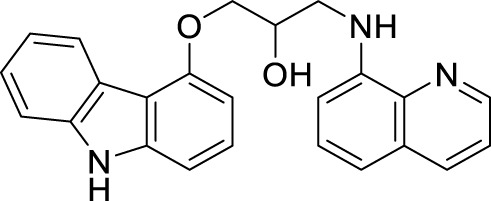	Cu-induced Aβ-aggregation inhibition = 22.9%	ThT fluorescence assay
68	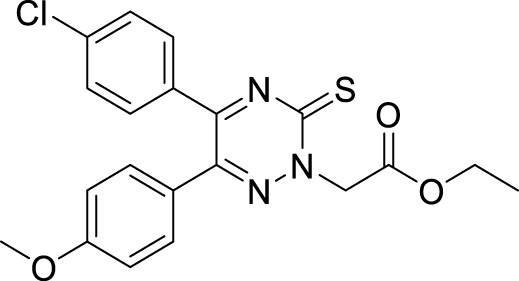	EC_50_ = 14.44 ± 0.85 µM	H_2_O_2_ toxicity assay
69	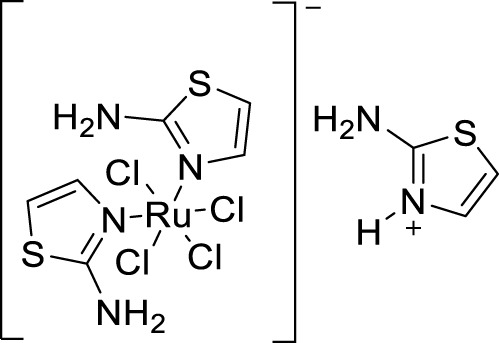	1 μM–40 μM	ThT fluorescence assay
70	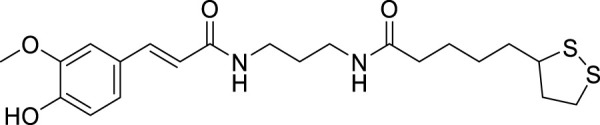	against Aβ1-42 (20 μM)	ThT fluorescence assay
71	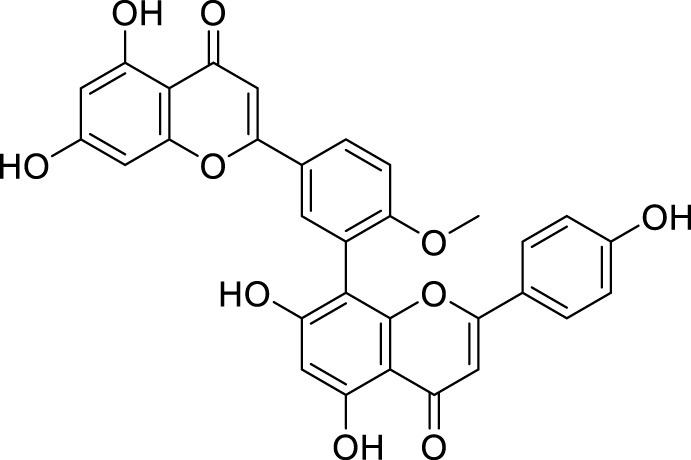	IC_50_ = 4.7 ± 0.7 μM	ThT fluorescence assay
72	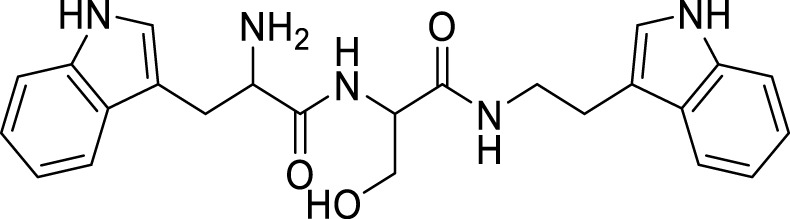	Aβ-aggregation inhibition IC_50_ = 49.8 ± 1.5 μM	- Anti-Amyloid Aggregation
73	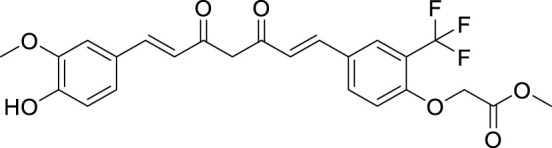	Inhibition of Aβ aggregation IC_50_ = 0.007 ± 0.001 μM	ThT fluorescence assay

### 4.3 BACE-1 inhibitors

Beta-site amyloid precursor protein cleaving enzyme (BACE-1), also known as *β-secretase* involved in Alzheimer’s pathogenesis, is a less explored target by the medicinal chemist for treating Alzheimer’s disease.


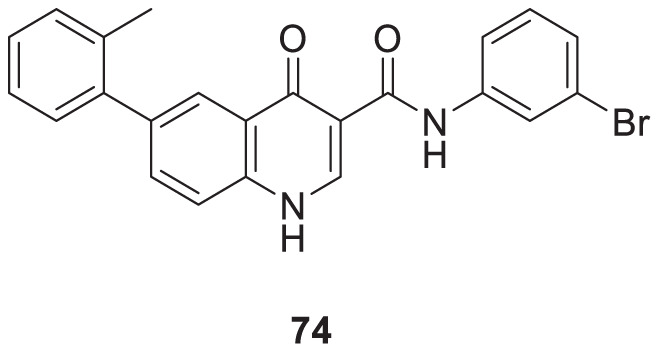


Liu et al. reported a series of 4-oxo-1,4-dihydro-quinoline-3-carboxamides as BACE-1 enzyme inhibitors. *N*-(3-bromophenyl)-4-oxo-6-(*o*-tolyl)-1,4-dihydroquinoline-3-carboxamide (**74**) was identified as a highly potent analog (IC_50_ = 1.89 ± 0.09 µM) and showed a high percentage of BACE-1 inhibition (77.6% ± 4.9%) evaluated by *fluorescence resonance energy transfer* (FRET) *assay*. Docking studies confirmed affinity to the enzyme’s active site. A good BBB permeability, and lower cellular toxicity were also observed for compound **74** (Liu et al., 2014).


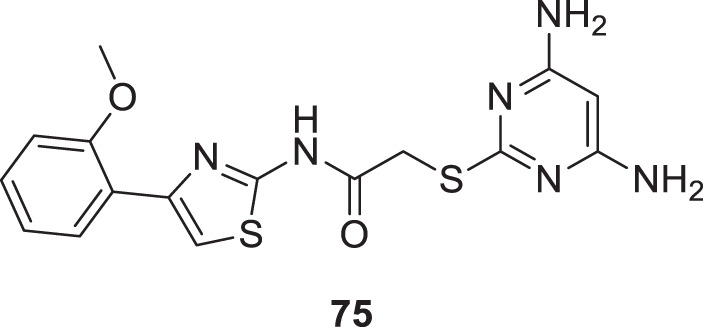


Xu et al. developed a series of 4-aminopyrimidine and 4,6-diaminopyrimidines against Aβ. 2-((4,6-diaminopyrimidin-2-yl)thio)-*N-*(4-(2-methoxyphenyl)thiazol-2-yl)acetamide (**75**) (IC_50_ = 1.4 ± 0.6 μM) was twenty-six-fold more potent than the lead compound as evaluated by FRET assay. Moreover, the parallel artificial membrane permeability assay suggested BBB permeability (Xu X. et al., 2019).


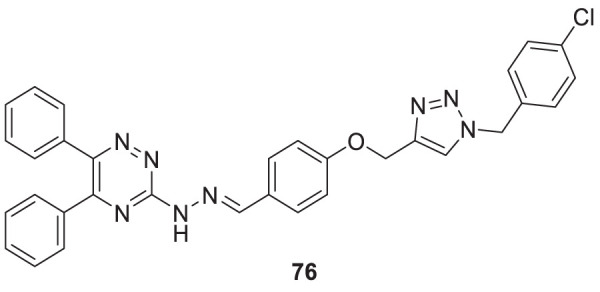


Yazdani et al. designed and synthesized 1,2,4-triazines bearing aryl phenoxy methyl-1,2,3-triazole against the BACE-1 enzyme. Researchers demonstrated that compounds having *chloro-* and *nitro-*substitution at the *para* position of the phenyl ring were potential BACE-1 inhibitors. (*E*)-3-(2-(4-((1-(4-chlorobenzyl)-1*H*-1,2,3-triazol-4-yl)methoxy) benzylidene)hydrazinyl)-5,6-diphenyl-1,2,4-triazine (**76**) with chlorine at the *para* position of the phenyl ring was a potent inhibitor (IC_50_ = 8.55 ± 3.37 μM). The neuroprotective activity was assessed on the PC12 neuronal cell line, and moderate neuronal protection was observed for the active analog. Docking studies revealed that these molecules have a high binding affinity to the enzyme’s active site (Yazdani et al., 2018).


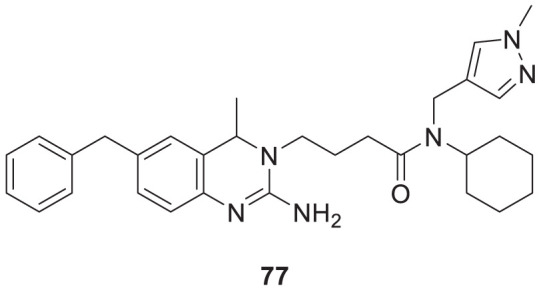


Jagtap et al. synthesized 4-substituted 2-amino-3,4-dihydro quinazoline with hairpin turn side chains as novel inhibitors for the BACE-1 enzyme. Among the derivatives, 4-(2-amino-6-benzyl-4-methylquinazolin-3(4*H*)-yl)-*N*-cyclohexyl-*N*-((1-*N*-methyl-1*H*-pyrazol-4-yl)methyl)butanamide (**77**), having 4-methyl substitution bearing *N*-cyclohexyl-*N*-(1-methyl-1*H*-pyrazol-4-ylmethyl)butanamide, exhibited potent BACE-1 enzyme inhibition with an IC_50_ of 0.38 μM. The docking study showed that the 3,4-dihydro quinazoline scaffold facilitates interaction with the *S*_*1*_, *S*_*2,*_ and *S*_*1*_ *'* subsites of the BACE-1, and the hairpin turn topology of the side chain provides additional interaction with the *S*_*2*_ subsite (Jagtap et al., 2020). See Table 2 for a summary of BACE-1 inhibitors.

**TABLE 2:** BACE-1 inhibitors.

Sr.No.	Compound	Activity	Assay type
74	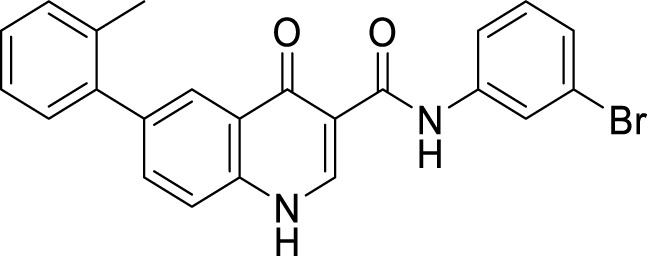	BACE-1 inhibition (77.6% ± 4.9%)	FRET assay
75	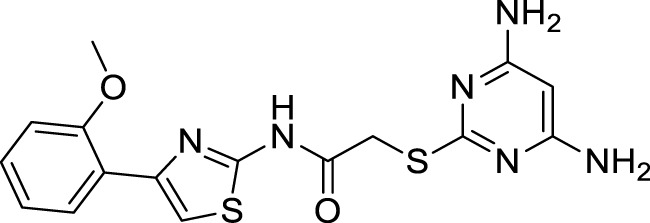	IC_50_ = 1.4 ± 0.6 μM	FRET assay
76	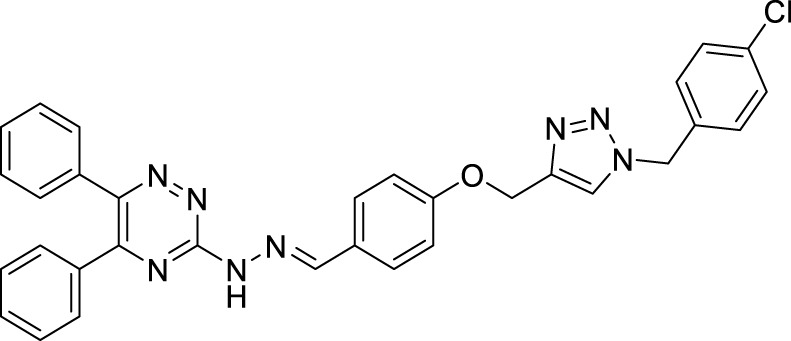	IC_50_ = 8.55 ± 3.37 μM	FRET assay
77	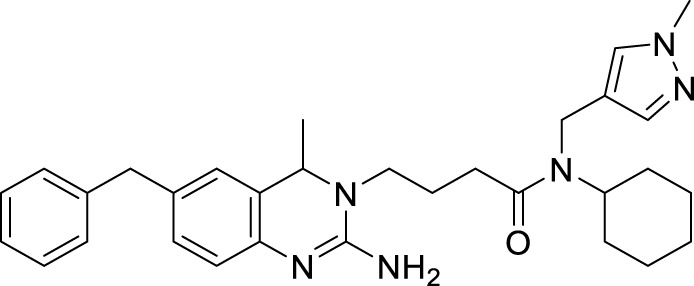	IC_50_ = 0.38 μM	BACE-1 inhibition assay

### 4.4 Glutaminyl cyclase inhibitors


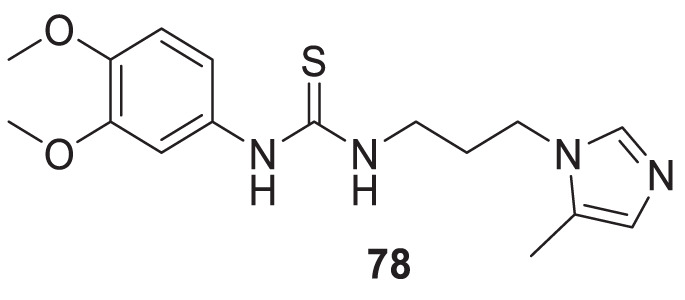


Hoang et al. conducted a study on glutaminyl cyclase (QC), a novel target, upon inhibition reduces the production of toxic pyriform of Aβ in the brain of Alzheimer’s patients. The researchers synthesized and evaluated a series of compounds, including *N*-substituted thiourea, urea, and α-substituted amide derivatives, for their ability to inhibit glutaminyl cyclase *in-vitro*. The synthesized compounds showed good potency in inhibiting glutaminyl cyclase, with 1-(3,4-dimethoxyphenyl)-3-(3-(5-methyl-1*H*-imidazol-1-yl)propyl)thiourea (**78**) exhibiting an IC_50_ value of 1.3 nM for inhibition of *hQC*. Structure-activity relationships (SAR) studies revealed that *N*-substitution increased potency by 20-fold for thiourea, 100-fold for urea, and 8-fold for amide derivatives compared to their unsubstituted counterparts. The *in-vivo* study conducted using the 5XFAD mouse model demonstrated that these compounds reduced the load of pyriform Aβ and total Aβ in the brain (Hoang et al., 2019).


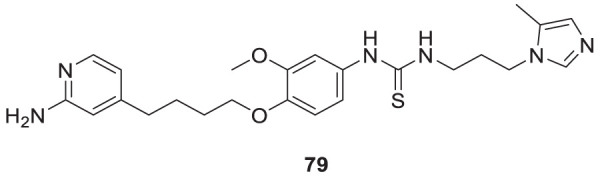


Hoang et al. developed a library of glutaminyl cyclase inhibitors based on the binding mode of Aβ3E-42 with glutaminyl cyclase. Among these compounds, 1-(4-(4-(2-aminopyridin-4-yl) butoxy)-3-methoxyphenyl)-3-(3-(5-methyl-1*H*-imidazol-1-yl)propyl)thiourea (**79**) demonstrated potent inhibitory activity against *hQC in-vitro*, with an IC_50_ value of 4.5 nM. *In-vivo* evaluation demonstrated that this compound effectively achieved the intended therapeutic outcomes. In two different transgenic mouse models of Alzheimer’s disease, APP/PS1 and 5xFAD, compound **79** significantly reduced both total Aβ and pyriform Aβ concentrations in the brain and restored cognitive function. Molecular docking studies revealed that compound **79** exhibited strong interactions with the *hQC* active site (PDB ID: 3PBB). Additionally, 5-methylimidazole chelated with zinc and formed hydrogen bond interactions (Hoang et al., 2017). See Table 3 for a summary of Glutaminyl Cyclase inhibitors.

**TABLE 3:** Glutaminyl cyclase inhibitors.

Sr.No.	Compound	Activity	Assay type
78	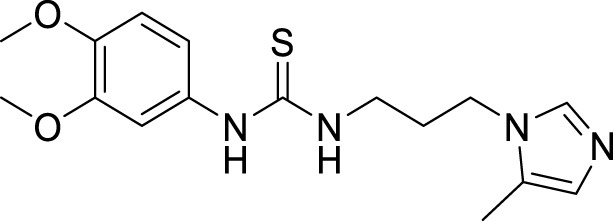	IC_50_ = 1.3 nM	inhibition of *hQC*
79	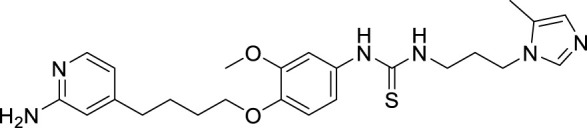	IC_50_ = 4.5 nM	inhibition of *hQC*

### 4.5 Improving brain function by HDAC modulation


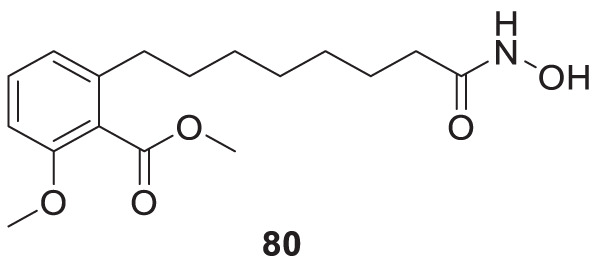


Romeiro et al. synthesized HDAC inhibitors derived from cashew nutshell liquid and its derivatives. Methyl 2-(8-(hydroxyamino)-8-oxooctyl)-6-methoxybenzoate (**80**) exhibited potent inhibition of HDAC1 and HDAC6, with IC_50_ values of 774.7 ± 14.4 nM and 215.4 ± 28.6 nM, respectively. Compound **80** also efficiently modulated glial cell-induced inflammation and reverted the pro-inflammatory phenotype (Romeiro et al., 2019). See Table 4 for a summary of HDAC inhibitors.

**TABLE 4:** HDAC inhibitors.

Sr.No.	Compound	Activity	Assay type
80	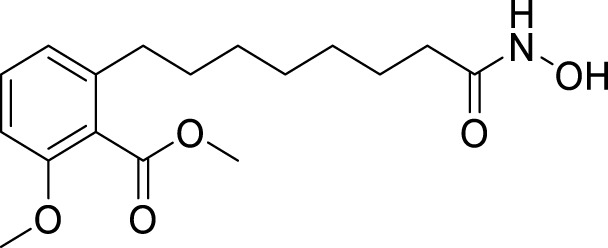	HDAC1 IC_50_ = 774.7 ± 14.4 nM HDAC6 IC_50_ = 215.4 ± 28.6 nM	inhibition of HDAC1 and HDAC6

### 4.6 Dual-target inhibitors

In the past decade, the impact of single-targeted therapies has been modest and transient due to the multifaceted nature of Alzheimer’s disease. Assorted studies have suggested that combination therapy could advance AD treatment despite the lack of evidence that these agents prevent or reverse the disease pathologies. Additionally, these agents prolong the time before the patient requires hospital care.


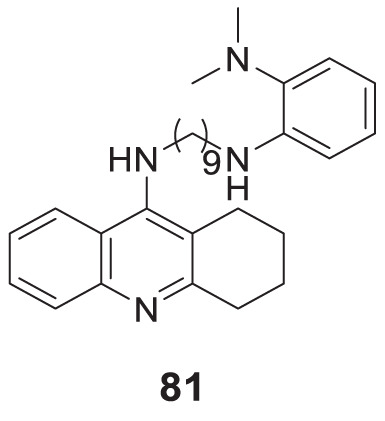


An effort made by Mao et al. designed and synthesized various compounds, including O-hydroxyl or O-amino benzylamine-tacrine hybrids (structure not shown) by reacting *N*-(amino-alkyl) tacrine with a salicylaldehyde or derivatives of 2-aminobenzaldehyde. These compounds were tested as multifunctional anti-Alzheimer’s agents against *AChE* and Aβ aggregates. *N* ^1^,*N* ^1^-dimethyl-*N* ^2^-(9-((1,2,3,4-tetrahydroacridin-9-yl)amino)nonyl)benzene-1,2-diamine (**81**) showed better *AChE* inhibition (IC_50_ = 0.55 ± 0.034 nM) and exhibited the potential to inhibit Aβ aggregates (39.4%) with additional antioxidant and metal chelating properties than tacrine. SAR studies revealed *AChE* inhibitory potency was closely related to the length of the alkylene chain. Hybrids with two, three, and four carbon spacers had weak inhibitory activity. *AChE* inhibitory activity intensified as the carbon spacer increases; the same trend was observed in both O-hydroxyl or O-amino benzylamine-tacrine hybrids. The most potent hybrid compound, **81** with a 9-carbon spacer, showed the highest inhibitory activity (Mao et al., 2012).


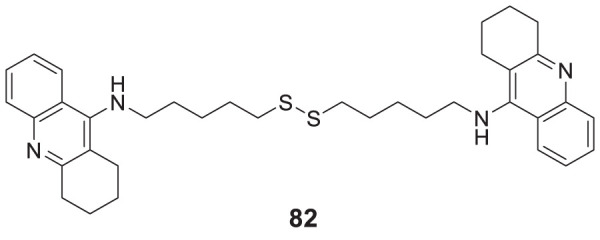


Another study by Roldan-Pena et al. designed tacrine-based homo- and heterodimers with antioxidant tether (selenoureido, dichalcogenide, or selenide) against *AChE* inhibition. Among these compounds, diselenides and disulfides containing dimers exhibited high activity against the *AChE* enzyme. *N,N'*-(disulfanediylbis (pentane-5,1-diyl))bis (1,2,3,4-tetrahydroacridin-9-amine) (**82**) with disulfide linker was the most potent compound showing strong inhibition against *hAChE* (IC_50_ = 1.62 ± 0.10 nM) and good inhibition against Aβ aggregates (61.8% ± 3.4%). Surprisingly, these compounds also displayed promising *anti-proliferative* activity tested against six human solid tumor cell lines and one non-tumor (BJ-hTert, human fibroblasts) cell line (Roldán-Peña et al., 2017).


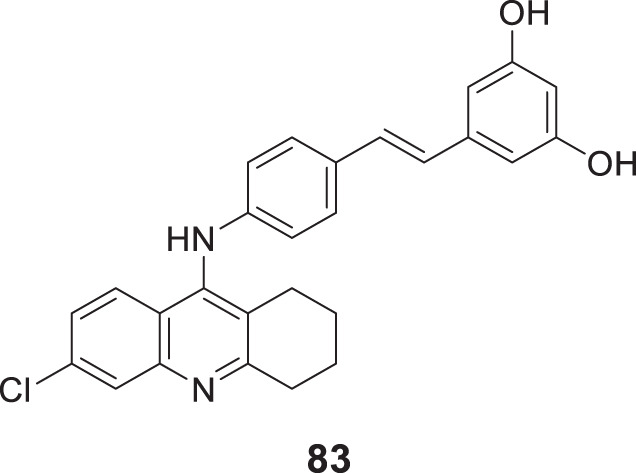


Similarly, Jerabek et al. also studied tacrine hybrids and combined structural features of tacrine with resveratrol having antioxidant and anti-neuroinflammatory activity as multi-target-directed ligands (MTDLs) for AD treatment. (*E*)-5-(4-((6-chloro-1,2,3,4-tetrahydroacridin-9-yl)amino)styryl)benzene-1,3-diol (**83**) was the most potent hybrid against *hAChE* (IC_50_ = 8.8 ± 0.4 µM). Also, it indicated higher inhibition of Aβ self-aggregation (IC_50_ = 31.2 ± 9.0 µM) than resveratrol. However, among the molecules studied, only compound **83** exhibited low neurotoxicity tested on an AD neuroinflammation cell model (Jeřábek et al., 2017).


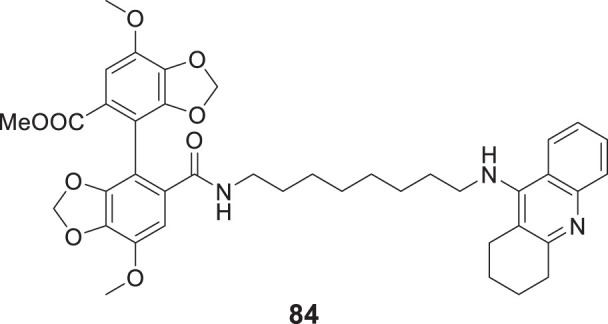


In an another interesting study, Cen et al. synthesized a series of tacrine-bifendate conjugates and evaluated their multi-model action as anti-Alzheimer’s agents. These compounds displayed potent cholinesterase and self-induced Aβ aggregation inhibitory activities with methyl 7,7′-dimethoxy-5'-((8-((1,2,3,4-tetrahydroacridin-9-yl)amino)octyl)carbamoyl)-[4,4′-bibenzo[*d*][1,3]dioxole]-5-carboxylate (**84**) being the most potent conjugate. Compound **84** displayed IC_50_ of 27.32 ± 1.61 nM for *AChE* inhibition and 82.5% ± 10.4% inhibition of Aβ aggregation. Further, a molecular modeling study demonstrated that these compounds target the acetylcholinesterase enzyme’s CAS and PAS sites. Compound **84** also revealed less cytotoxicity on PC12, HepG2, and human liver cell lines (HL-7702) than tacrine (Cen et al., 2017).


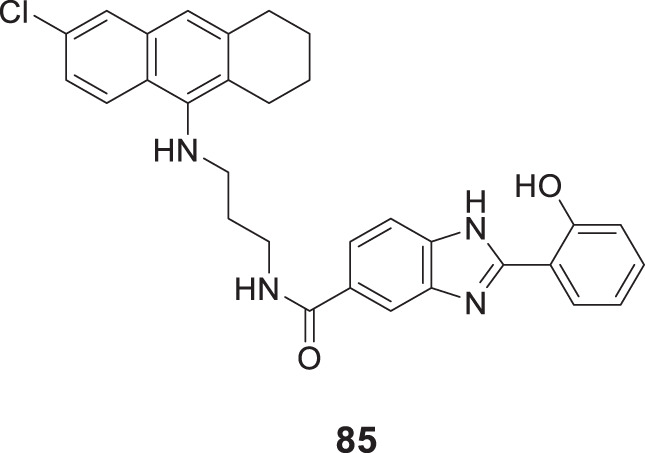


In an alternative exciting research, Hiremathad et al. designed a series of tacrine-hydroxy phenyl benzimidazole (TAC-BIM) as multi-targeted drug ligands (MTDL). *N*-(3-((6-chloro-1,2,3,4-tetrahydroanthracen-9-yl)amino)propyl)-2-(2-hydroxyphenyl)-1*H*-benzo [*d*]imidazole -5-carboxamide (**85**) indicated an improved *AChE* inhibitory activity (IC_50_ = 6.3 nM). Also, it exhibited high inhibition of self-induced and Cu-induced Aβ aggregation (inhibition = 39.4%). Additionally, the synthesized compound displayed moderate radical scavenging activity and metal chelating ability. SAR studies demonstrated that chlorine substitution on tacrine is beneficial for enzyme inhibition due to good fitting at the enzyme active site. Further, the analysis speculated that compound **85** had dual binding capacity with CAS and PAS of acetylcholinesterase enzyme due to π-π stacking with three aromatic residues. Neurotoxicity studies revealed that these compounds could inhibit neurotoxicity in neuronal cells (Hiremathad et al., 2018b).


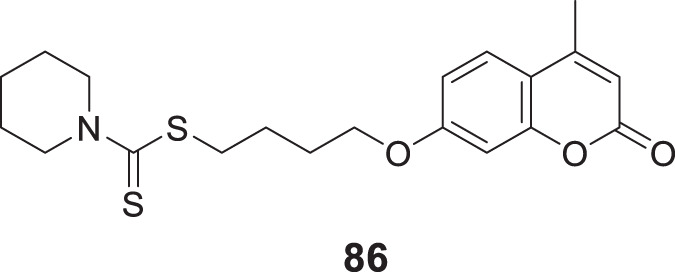


In fascinating research, Jiang et al. reported the first dithiocarbamates with multifunctional activity against AD. Co-workers designed, synthesized, and evaluated novel coumarin-dithiocarbamate hybrids for AD treatment. Biological assay indicated 4-((4-methyl-2-oxo-2*H*-chromen-7-yl)oxy)butyl piperidine-1-carbodithioate (**86**) as potent, selective *hAChE* inhibitory activity (IC_50_ = 0.027 ± 0.002 μM) and moderate Aβ aggregation inhibition (40.19% ± 2.39%). Kinetic and molecular modeling analysis revealed that compound **86** displayed mixed-type inhibition and interacted well with CAS and PAS of *hAChE*. In addition, it also enjoyed the metal chelating ability and low toxicity in SH-SY5Y neuroblastoma cells (Jiang N. et al., 2018).


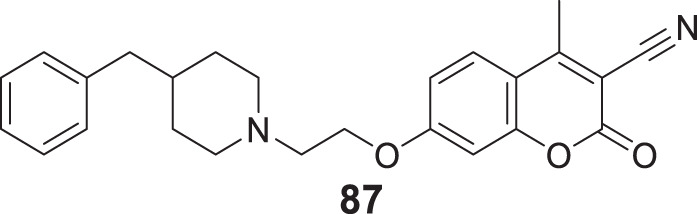


Joubert et al. developed a series of 7-substituted coumarins to study cholinesterase and monoamine oxidase-B (MAO-B) inhibitory activity. These derivatives consisted of a coumarin structure resembling MAO-B inhibitor and a piperidine moiety resembling the 4-benzyl piperidine function of donepezil for *AChE* inhibition connected via alkyl ether linkage at the seventh position of coumarin. The biological activity assessment indicated that all compounds effectively inhibited human MAO-B over MAO-A. 7-(2-(4-Benzylpiperidin-1-yl)ethoxy)-4-methyl-2-oxo-2*H*-chromene-3-carbonitrile (**87**) emerged as the most active derivative against *AChE* (IC_50_ = 9.10 µM) and MAO-B (IC_50_ = 0.30 µM). Molecular modeling suggested that compound **87** can bind to CAS, mid-gorge, and PAS sites of the *AChE* (Joubert et al., 2017).


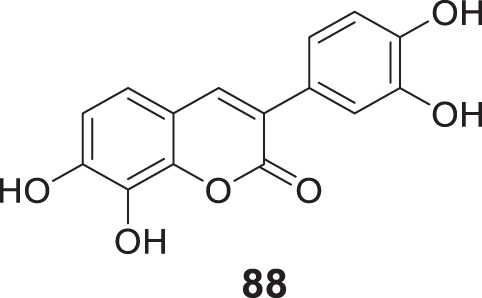


Yang et al. designed and synthesized 3-aryl coumarins and investigated their cholinesterase and MAO inhibitory activity. MAO are one of the enzymes whose levels are increased in neurodegenerative disorders; consequently, MAO inhibitors are explored as a complementary alternative in search of new anti-Alzheimer’s agents. Most of the derivatives exhibited moderate to excellent activity; 3-(3,4-dihydroxyphenyl)-7,8-dihydroxy-2*H*-chromen-2-one (**88**) displayed the highest activity against *AChE* (IC_50_ = 3.04 ± 0.32 μM) and MAO-B (IC_50_ = 27.03 ± 0.50 μM) (Yang et al., 2019).


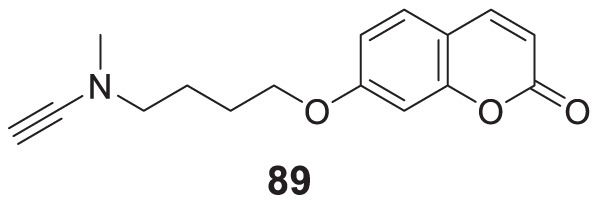


Yang et al. designed, synthesized, and evaluated a series of coumarin-pargyline hybrids as dual inhibitors against Alzheimer’s disease. In particular, 7-(4-(ethynyl (methyl)amino)butoxy)-2*H*-chromen-2-one (**89**) depicted good inhibitory activity against human MAO-A (IC_50_ = 3.275 ± 0.040 µM), MAO-B (IC_50_ = 0.027 ± 0.004 µM) and A*β* aggregation (54%). Moreover, compound **89** indicated low *in-vitro* cytotoxicity (Yang et al., 2017).


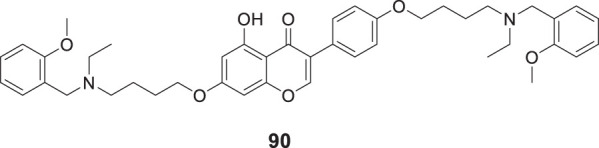


In a separate research study, Qiang et al. reported the design and synthesis of genisteins with carbon spacer-linked alkyl benzylamines as a multifunctional agent against AD. Most of the compounds of the series exhibited potent anti-*AChE* activity and showed high selectivity for *AChE* over *BuChE*. 7-(4-(Ethyl(2-methoxybenzyl)amino)butoxy)-3-(4-(4-(ethyl (2-methoxybenzyl)amino)butoxy)phenyl)-5-hydroxy-4*H*-chromen-4-one (**90**) displayed sub-micromolar *hAChE* inhibition potency (IC_50_ = 0.35 ± 0.03 µM) and moderately inhibited self-induced Aβ aggregation (inhibition = 35.0 ± 1.0%). Also, it expressed high inhibition of Cu-induced Aβ aggregation with less antioxidant effect. A molecular modeling study suggested that compound **90** showed a mixed-type of inhibition binding to both CAS and PAS of *AChE*. Due to multifunctional properties, compound **90** can be a promising candidate against AD (Qiang et al., 2014).


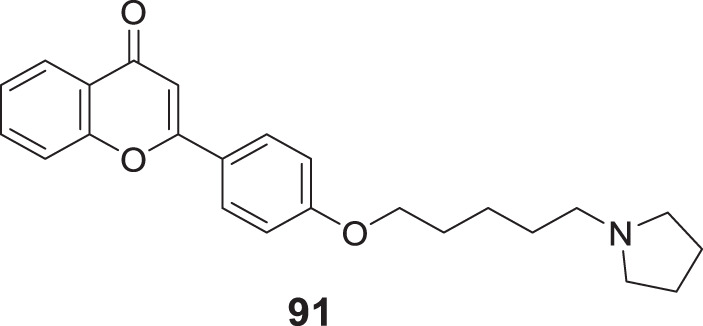


Faraji et al. designed seventeen amino alkyl-substituted flavonoids were evaluated for anti-*AChE* and anti-self-induced Aβ aggregation activity. Among them, 2-(4-((5-(pyrrolidin -1-yl)pentyl)oxy)phenyl)-4*H*-chromen-4-one (**91**) demonstrated the best anti-*AChE* activity (IC_50_ = 0.01 ± 0.001 μM) and also inhibited self-induced Aβ aggregation (49.2% ± 1.3%). In addition, compound **91** also expressed a neuroprotective effect in PC12 neurons against H_2_O_2_-induced cell death (Faraji et al., 2019).


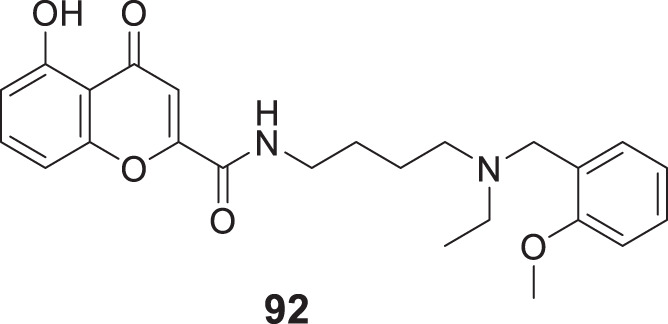


A similar type of study was performed by Liu et al. who designed chromone-2-carboxamido-alkylbenzylamines as multifunctional agents against AD. Among the derivatives, *N*-(4-(ethyl (2-methoxybenzyl)amino)butyl)-5-hydroxy-4-oxo-4*H*-chromene-2-carboxamide (**92**) displayed excellent inhibitory potency against *AChE* (IC_50_ = 0.07 ± 0.01 µM) and a good inhibitory effect towards self-induced Aβ aggregation (59.2% ± 1.6%) with moderate antioxidant and selective metal chelating activity. A molecular modeling study revealed that compound **92** is a mixed-type inhibitor binding to both CAS and PAS of *AChE*. SAR studies have suggested that substitutions at the 7-position significantly affected *AChE* inhibitory activities (Liu et al., 2015).


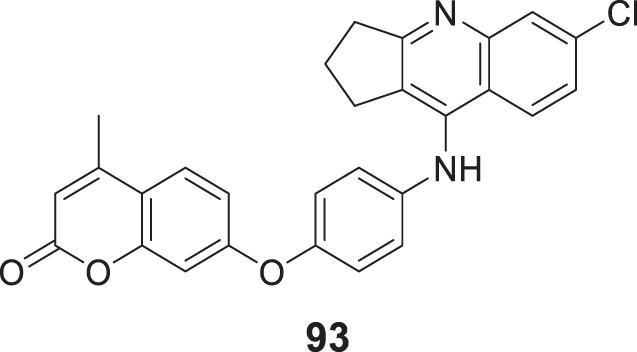


In an another study, Najafi et al. designed a novel series of chromenone hybrids as anti-Alzheimer’s agents. Compound **93** [7-(4-(6-chloro-2,3-dihydro-1*H*-cyclopenta [*b*]quinolin-9-ylamino)phenoxy)-4-methyl-2*H*-chromen-2-one] exhibited the highest *AChE* inhibitory activity (IC_50_ = 16.17 ± 0.02 µM) however, had lower *AChE* inhibitory activity than reference drug rivastigmine (IC_50_ = 11.07 µM). In addition, compound **93** demonstrated *β-secretase* (BACE-1) inhibitory activity with IC_50_ of 7.99 ± 0.916 µM. Kinetic and molecular modeling studies revealed that compound **93** exhibited good interaction with the CAS and PAS of the *AChE*; further satisfactory neuroprotection was observed (Najafi et al., 2016).


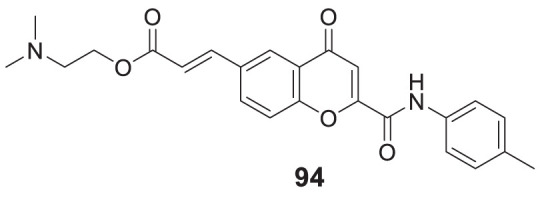


Reis et al. used a chromone scaffold, developed a small library of chromones, and screened against human cholinesterase and MAOs. Among them, compound **94** [2-(dimethylamino)ethyl (*E*)-3-(4-oxo-2-(*p*-tolylcarbamoyl)-4*H*-chromen-6-yl)acrylate] a most promising multi-target inhibitor displaying dual activity against human *AChE* (IC_50_ = 3.69 ± 0.24 μM) and MAO-B (IC_50_ = 0.63 ± 0.01 μM). Overall, compound **94** stands out as a reversible multi-target inhibitor with favourable permeability and toxicological profiles (Reis et al., 2018).


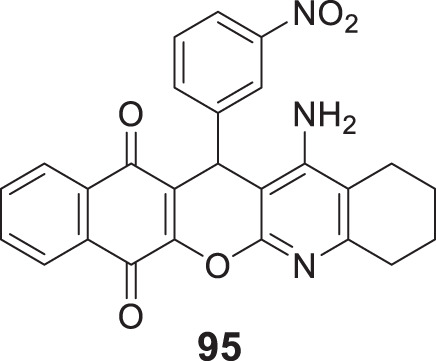


In a separate study, Mahdavi et al. designed and synthesized benzochromenoquinolines and evaluated them for their cholinesterase and BACE-1 inhibitory activities. Among the synthesized compounds, compound **95** (14-amino-13-(3-nitrophenyl)-2,3,4,13-tetrahydro-1*H*-benzo [6,7]chromeno [2,3-*b*]quinoline-7,12-dione) exhibited the highest *AChE* (IC_50_ = 0.86 ± 0.04 μM) and *BuChE* (IC_50_ = 6.03 ± 0.34 μM) inhibitory activity. Also, it depicted potential BACE-1 inhibition (IC_50_ = 19.60 ± 0.9 μM) evaluated by FRET assay. In addition, compound **95** also displayed metal chelating ability, and docking results suggested good interaction with the active site (Mahdavi et al., 2019).


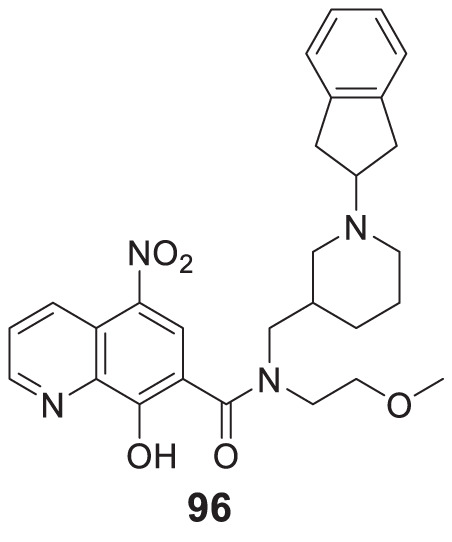


Knez et al. reported the synthesis and evaluation of nitroxoline-based analogues designed by combining an 8-hydroxyquinoline scaffold with that of known selective *BuChE* inhibitors, *N*-((1-(2,3-dihydro-1*H*-inden-2-yl)piperidin-3-yl)methyl)-8-hydroxy-*N*-(2-methoxyethyl)-5-nitro quinoline-7-carboxamide (**96**) showed the best *hBuChE* inhibition (IC_50_ = 0.215 µM) and self-induced Aβ aggregation inhibition (20.1% ± 2.0%). The docking study revealed good interaction of this molecule with the active site, suggesting that chelation of 5-nitroquinolin-8-ol fragment positioned in the acyl binding pocket of *huBuChE* results in additional interaction with active site residues resulting in increased inhibitory activity (Knez et al., 2015).


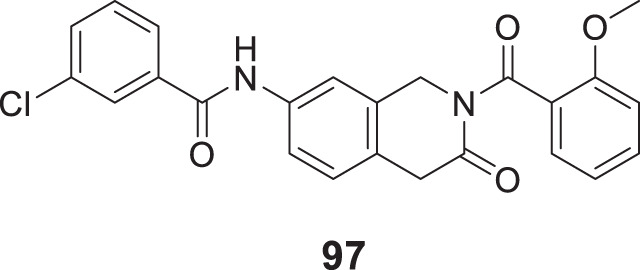


Zhao et al. developed a new class of MTDLs based on 7-amino-1,4-dihydro-2*H*-isoquinolin-3-one for *AChE* and *β-secretase* inhibition. Molecular modeling suggested three aromatic moieties interact with CAS and PAS of *AChE,* and the amide bond enables interaction with BACE-1. *In-vitro* studies revealed that 3-chloro-*N*-(2-(2-methoxybenzoyl)-3-oxo-1,2,3,4-tetrahydroisoquinolin-7-yl)benzamide (**97**) exerted excellent *AChE* (IC_50_ = 18.93 ± 1.02 pM) and *β-secretase* (97.68%) inhibition (Zhao et al., 2017).


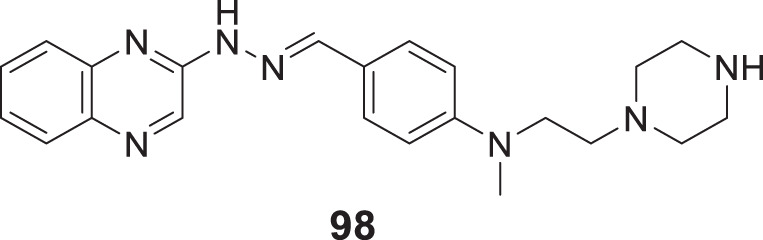


Cevik et al. designed and synthesized various substituted quinoxaline-hydrazones and their *in-vitro* activities were investigated, including cholinesterase and MAO inhibitory activity. (*E*)-*N*-methyl-*N*-(2-(piperazin-1-yl)ethyl)-4-((2-(quinoxalin-2-yl)hydrazineylidene) methyl)aniline (**98**) exhibited nanomolar inhibitory potency for *AChE* (IC_50_ = 0.028 ± 0.001 μM) and MAO-B (IC_50_ = 0.046 ± 0.002 μM) activity. Molecular modeling suggested that compound **98** could bind to the active site of *AChE* and MAO-B (Çevik et al., 2020).


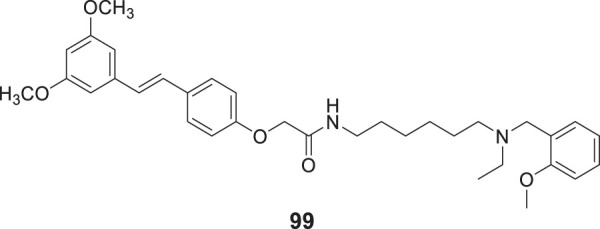


Li et al. developed a novel series of pterostilbene-O-acetamido alkyl benzylamines against AD. The derivatives were evaluated as dual inhibitors for *AChE* and *BuChE*. They also explored the derivatives for antioxidant, self-induced Aβ aggregation, and *hAChE*-induced Aβ aggregation inhibition activities. (*E*)-2-(4-(3,5-dimethoxystyryl)phenoxy)-*N*-(6-(ethyl (2-methoxybenzyl)amino)hexyl)acetamide (**99**) was identified as the most active derivative and displayed good inhibitory activity against *AChE* (IC_50_ = 0.06 ± 0.03 µM) and *BuChE* (IC_50_ = 28.04 ± 1.71 µM). Satisfactory activity was observed against self-induced Aβ aggregation (32.4% ± 1.0%), and *hAChE* inhibited Aβ aggregation. Kinetic analysis and molecular modeling studies revealed that this derivative exhibited mixed-type inhibition with a binding affinity towards both CAS and PAS of the *AChE* (Li Y. et al., 2016).


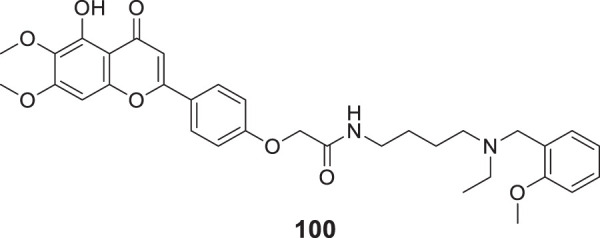


Similarly, Sang et al. reported the design and synthesis of acetamido alkylbenzylamines replacing pterostilbene with scutellarein as multifunctional agents for treating AD. *N*-(4-(ethyl (2-methoxybenzyl)amino)butyl)-2-(4-(5-hydroxy-6,7-dimethoxy-4-oxo-4*H*-chromen-2-yl)phenoxy)acetamide (**100**) was identified as a highly active derivative exhibiting *hAChE* inhibition (IC_50_ = 0.039 ± 0.002 µM), moderate self-induced Aβ aggregation inhibition (57.1% ± 1.9%), Cu-induced Aβ aggregation inhibition, and *hAChE* induced Aβ aggregation inhibition. Also, compound **100** acted as a potential biometal chelator and antioxidant. The improved *AChE* inhibitory activity was found to be due to good interaction of this molecule with CAS and PAS of *AChE*. The neuroprotective effect was studied on H_2_O_2_-induced PC12 cell line and low toxicity in SH-SY5Y cells. Moreover, the scopolamine-induced memory deficit was reversed in mice (Sang et al., 2017b).


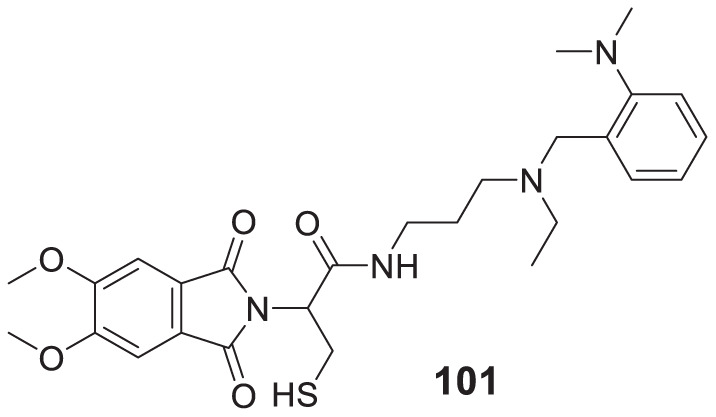


Further exploring the alkyl benzylamine scaffold, Zhang et al. developed a series of phthalimide-(*N*-alkylbenzylamine)cysteamide hybrids based on the *multitarget directed ligands* (MTDL) strategy. *In-vitro* study showed that 2-(5,6-dimethoxy-1,3-dioxoisoindolin-2-yl)-*N*-(3-((2-(dimethylamino)benzyl) (ethyl)amino)propyl)-3-mercaptopropanamide (**101**) displayed high *EeAChE* (IC_50_ = 1.55 ± 0.17 μM) and *hAChE* (IC_50_ = 2.23 ± 0.04 μM) inhibition potency, good self-induced Aβ aggregation inhibition (36.08%) and moderate antioxidant effect. Molecular docking revealed the binding of the compound **101** to CAS and PAS of *AChE*. The neuroprotective effect was also observed against the H_2_O_2_-induced PC12 cell line (Zhang H. et al., 2021).


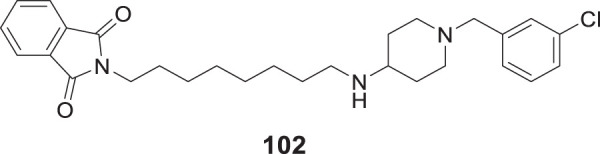


Another study conducted by Wieckowska et al., based on the MTDL approach, designed and synthesized a novel series of *N*-benzyl piperidines containing *N*-benzyl piperidine moiety combined with phthalimide or isoindole moieties. The most promising results were displayed by compound **102** [(2-(8-(1-(3-chlorobenzyl)piperidin-4-ylamino)octyl)isoindoline-1,3-dione)] against *BuChE* (IC_50_ = 0.72 ± 0.038 µM) and Aβ aggregation inhibition (72.5%). Also, improved memory was observed in the scopolamine-induced animal model. Kinetic and docking studies revealed a good molecular interaction with the active site of *BuChE* (Więckowska et al., 2015).


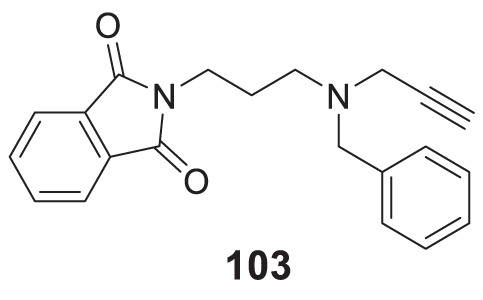


A group of researchers led by Sang et al. also developed a series of phthalimide-alkylamines as multi-functional agents for treating Alzheimer’s disease. Among them, 2-(3-(benzyl (prop-2-yn-1-yl)amino)propyl)isoindoline-1,3-dione (**103**) was identified as the most active molecule exhibiting potent and balanced inhibitory activity towards human *AChE* (IC_50_ = 1.2 ± 0.07 µM) and MAO-B (IC_50_ = 2.6 ± 0.05 µM). Kinetic analysis of *AChE* inhibition and molecular modeling revealed that compound **103** binds to the CAS and PAS site of *AChE*. Furthermore, this molecule was less cytotoxic (Sang et al., 2017d).


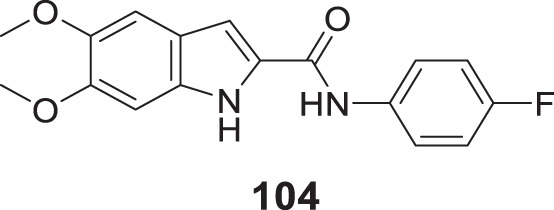


Koca et al. designed and evaluated a series of 5,6-dimethoxy-1*H*-indene-2-carboxamides as multifunctional drug candidates for AD. *N*-(4-fluorophenyl)-5,6-dimethoxy-1*H*-indole-2-carboxamide (**104**) was identified as the most potent derivative with *AChE* (IC_50_ = 2.33 ± 0.021 µM), *BuChE* (IC_50_ = 1.08 ± 0.011 µM), and Aβ aggregation (50.3% ± 2.4%) inhibition. These results suggested that compound **104** exhibited more inhibitory activity against *BuChE* than *AChE*. Kinetic analysis revealed that these compounds act as non-competitive inhibitors, and docking studies demonstrated the presence of many potential hydrogen bonding interactions with the PAS of *BuChE* (Koca et al., 2016).


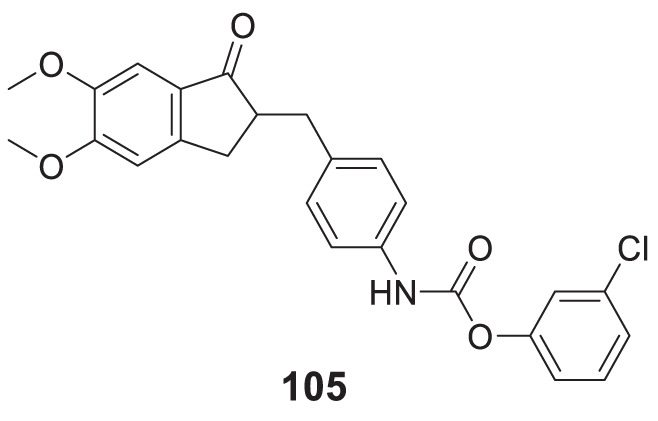


Further, Shahrivar-Gargari et al. studied the carbamates and designed novel indanone-carbamate hybrids by pharmacophore hybridization-based design strategy as anti-Alzheimer’s agents. 3-Chlorophenyl (4-((5,6-dimethoxy-1-oxo-2,3-dihydro-1*H*-inden-2-yl)methyl)phenyl)carbamate (**105**) showed the highest inhibition of *EeAChE* (IC_50_ = 3.04 ± 0.94 μM), and potent Aβ aggregation (77.5%). Kinetic studies indicated a reversible partial non-competitive type of inhibition. The indanone-carbamate scaffold can be structurally modified and optimized to design novel multitargeted agents against AD (Shahrivar-Gargari et al., 2021).


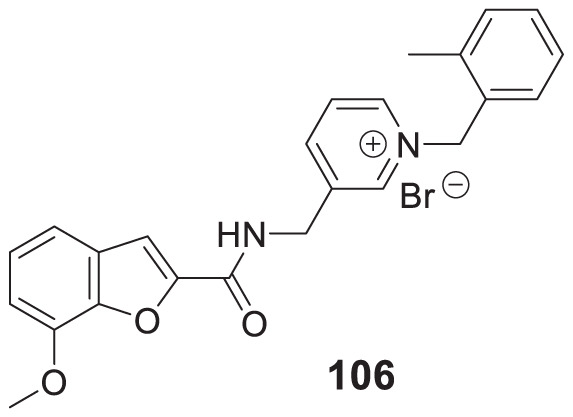


However, Abedinifar et al. developed and synthesized benzofuran-2-carboxamide-*N*-benzyl pyridinium halides as novel cholinesterase inhibitors. *In-vitro* studies revealed that these derivatives are potent inhibitors. Among them, 3-((7-methoxybenzofuran-2-carboxamido)methyl)-1-(2-methylbenzyl)pyridin-1-ium bromide (**106**) was more potent against *BuChE* (IC_50_ = 0.45 ± 0.05 µM) than *AChE* (IC_50_ = 2.1 ± 0.1 µM). In addition, a good inhibitory effect on self-induced Aβ aggregation (46.4% ± 2.2%) was also observed. Docking studies revealed hydrogen bonding interaction with the oxygen atom of benzofuran, the amide group’s nitrogen atom, and the benzofuran methoxy moiety (Abedinifar et al., 2018).


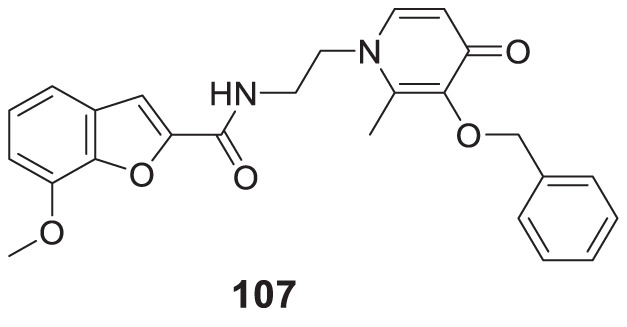


While Hiremathad et al. synthesized a series of (3-hydroxy-4-pyridine)-benzofuran hybrids targeting AD. The activity of these molecules was compared with donepezil and *N*-(2-(3-(benzyloxy)-2-methyl-4-oxopyridin-1(4*H*)-yl)ethyl)-7-methoxybenzofuran-2-carboxamide (**107**) emerged as the most active hybrid demonstrating action against multiple targets of AD including *AChE* inhibitory activity (IC_50_ = 76 µM) and self-induced Aβ aggregation inhibition (36.1%) (Hiremathad et al., 2018a)


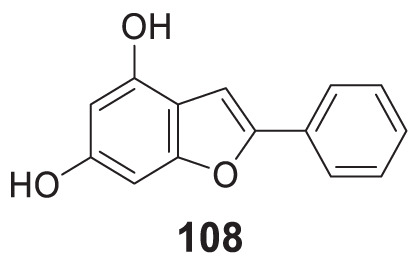


Another study performed by Yun et al. designed and synthesized a series of 2-aryl benzofurans as dual cholinesterase and *β-secretase* inhibitors. 2-Phenylbenzofuran-4,6-diol (**108**) demonstrated the potent inhibitory activity against *AChE* (IC_50_ = 0.086 ± 0.01 µmolL^-1^), *BuChE* (IC_50_ = 16.450 ± 2.12 µmolL^-1^), and *β-secretase* (IC_50_ = 0.043 ± 0.01 µmolL^-1^) due to the presence of hydroxyl groups. Compound **108** also displayed low neurotoxicity against normal cells (Yun et al., 2021).


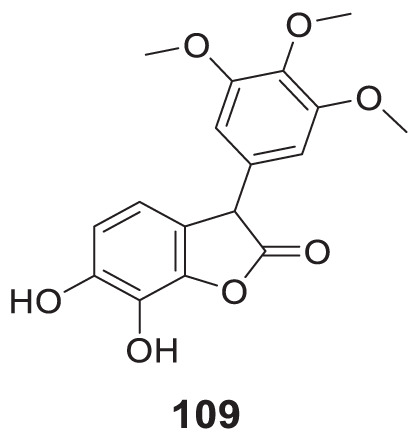


Yang et al. designed a series of 3-arylbenzofuranones and evaluated their cholinesterase and MAO inhibitory activity. Among the series of derivatives, 6,7-dihydroxy-3-(3,4,5-trimethoxyphenyl)benzofuran-2(3*H*)-one (**109**) exhibited potent inhibitory activity against *AChE* (IC_50_ = 0.089 ± 0.01 μM) and moderate inhibitory activity against MAO-B (IC_50_ = 149.21 ± 3.39 μM) (Yang et al., 2020).


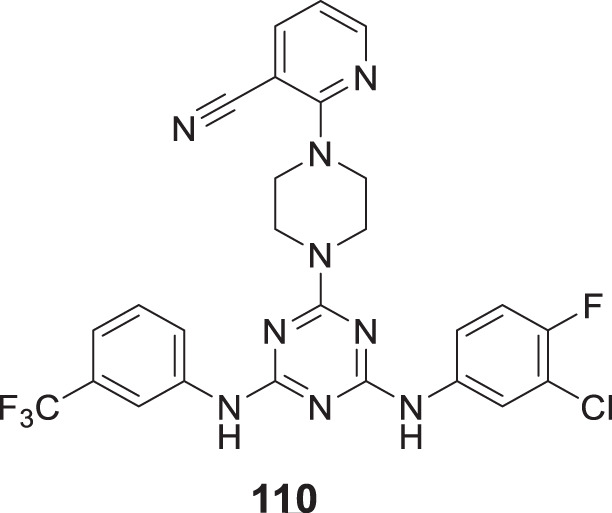


Later, Maqbool et al. developed cyanopyridine-triazine hybrids and screened them as multitargeted anti-Alzheimer agents. Among the hybrids, 2-(4-(4-((3-chloro-4-fluorophenyl)amino)-6-((3-(trifluoromethyl)phenyl)amino)-1,3,5-triazin-2-yl)piperazin-1-yl) nicotinonitrile (**110**) exhibited high potency for *AChE* inhibition (IC_50_ = 0.059 ± 0.003 µM) with more selectivity for *AChE* over *BuChE* and self-induced Aβ aggregation inhibition (83.7% ± 1.13%). Molecular modeling studies revealed these compounds’ interaction with CAS and PAS of *AChE*. Neuroprotection studies revealed reduced neuronal death induced by H_2_O_2_-mediated oxidative stress, and *in silico* studies confirmed drug-like properties (Maqbool et al., 2016).


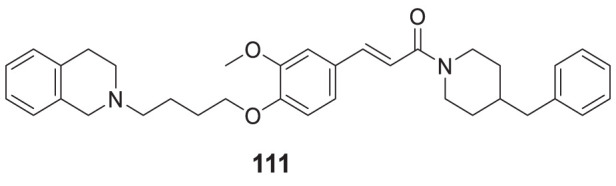


Continuing the MTDL approach, Sang et al. developed a series of ferulic acid-O-alkylamines for AD treatment. *In-vitro* studies indicated these derivatives exhibited impressive *BuChE* and Aβ aggregation inhibition. Notably, (*E*)-1-(4-benzylpiperidin-1-yl)-3-(4-(4-(3,4-dihydroisoquinolin-2(1*H*)-yl)butoxy)-3-methoxyphenyl)prop-2-en-1-one (**111**) was the most potent inhibitor against *equine serum BuChE* (IC_50_ = 2.13 ± 0.01 µM), *rat BuChE* (IC_50_ = 1.8 ± 0.02 µM) and *human serum BuChE* (IC_50_ = 3.82 ± 0.05 µM). Also, potent Aβ aggregation inhibition (50.8% ± 0.82%) and antioxidant activity were indicated. The neuroprotective effect of the active compound with low toxicity against H_2_O_2_-induced PC12 cell injury and the step-down avoidance test of compound **111** marked a significant reversal of scopolamine-induced memory deficit (Sang et al., 2017a).


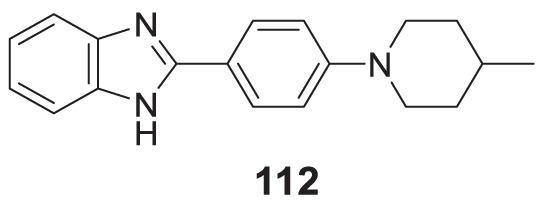


Exploring the multifunctional agents, Unsal-Tan et al. developed and synthesized 2-aryl benzimidazoles as multitarget agents against AD. *In-vitro* studies indicated 2-(4-(4-methylpiperidin-1-yl)phenyl)-1*H*-benzo [*d*]imidazole (**112**) with good inhibitory activity for *BuChE* (IC_50_ = 39.56 μM) and also displayed good Aβ anti-aggregation (67.78% ± 0.16%). A molecular modeling study revealed that the compound could reach the catalytic site of *BuChE* but not *AChE*. In addition, compound **112** displayed a neuroprotective effect against H_2_O_2_-induced cell death (Unsal-Tan et al., 2017).


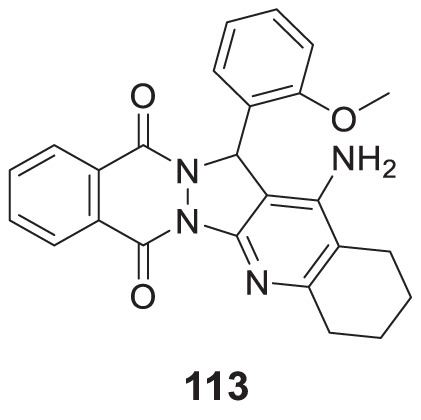


Jalili-Baleh et al. worked on tacrine-like compounds bearing fused pyrazolo [1,2-*b*] phthalazines and screened for AD treatment. Among them, 15-amino-14-(2-methoxyphenyl)-2,3,4,14-tetrahydro-1*H*-quinolino [2′,3':3,4]pyrazolo [1,2-*b*]phthalazine-7,12-dione (**113**) exhibited higher anti-*AChE* activity (IC_50_ = 0.049 µM) than tacrine with high selectivity for *AChE* over *BuChE*. Also, the anti-aggregation effect against self-induced Aβ aggregation (25.5% ± 2.9%) was evaluated by ThT fluorescence assay. Cell-based screening of compound **113** against hepatocytes (HepG2) and neuronal cell line (PC12) reported lower toxicity as compared tacrine (Jalili-Baleh et al., 2017).


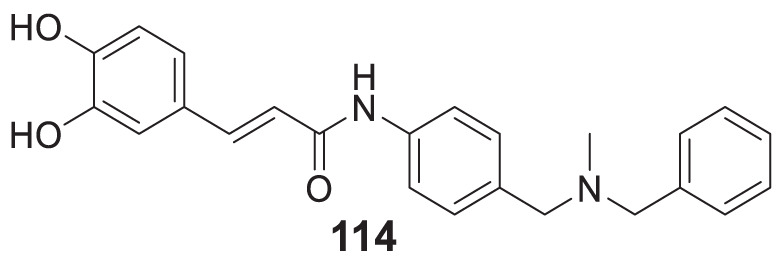


Another research focused on multitarget agents by Wang et al. designed and synthesized a novel series of cinnamide-dibenzyl amine hybrids against AD. (*E*)-*N*-(4-((benzyl (methyl)amino)methyl)phenyl)-3-(3,4-dihydroxyphenyl)acryl amide (**114**) exhibited significant inhibitory activity against *EeAChE* (IC_50_ = 4.64 ± 0.23 µM), *hAChE* (IC_50_ = 5.42 ± 0.25 µM), and self-induced Aβ aggregation (56.2% ± 1.21%) with antioxidant and neuroprotective activity and reduced cell death due to oxidative stress in PC12 cell lines. A molecular modeling study suggested that compound **114** targeted both CAS and PAS of *AChE* (Wang et al., 2017).


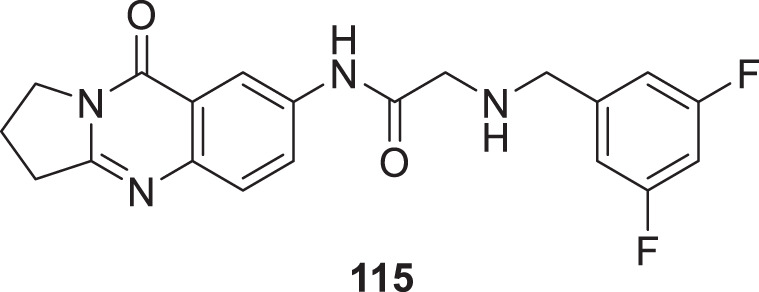


Ma et al. designed a series of multitarget ligands deoxyvasicinones by introducing diverse amino acetamide groups at position 6 of the deoxyvasicinone group. *In-vitro* studies identified 2-((3,5-difluorobenzyl)amino)-*N*-(9-oxo-1,2,3,9-tetrahydropyrrolo [2,1-*b*]quinazolin-7-yl)acetamide (**115**) as the most active compound against *hAChE* (IC_50_ = 7.61 ± 0.53 µM) and exhibited moderate to high self-induced Aβ aggregation inhibition (63.9% ± 4.9%). The kinetic analysis confirmed that compound **115** displayed mixed-type inhibition with binding affinity to both CAS and PAS of *hAChE* (Ma and Du, 2017).


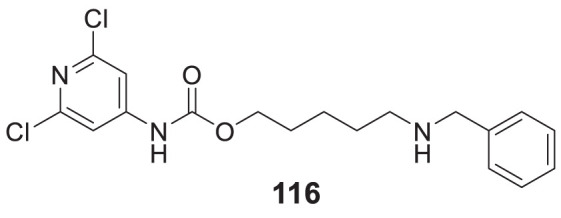


Pandolfi et al. designed pyridines with carbamic or amidic function as cholinesterase inhibitors. Among the series, 5-(benzylamino)pentyl (2,6-dichloropyridin-4-yl)carbamate (**116**) bearing carbamate moiety exhibited the most potent *hAChE* inhibition (IC_50_ = 0.153 ± 0.016 µM). A molecular docking study indicated that compound **116** could bind to *AChE* by interacting with the enzyme’s CAS and PAS with mixed inhibition. Furthermore, the active compound displayed self-induced Aβ aggregation inhibition (26.5% ± 1.2%) with relatively low toxicity against human astrocytoma T67 and HeLa cell lines (Pandolfi et al., 2017).


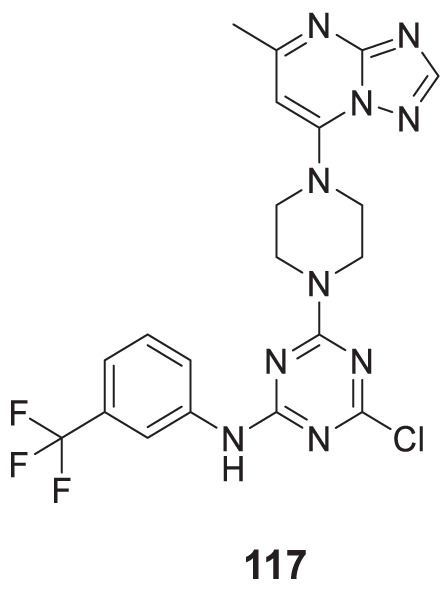


Similarly, Jameel et al. also studied multitarget ligands and developed a series of triazine-triazolo pyrimidine hybrids for AD treatment. Among seventeen synthesized compounds, di-substituted triazine-triazole pyrimidine hybrids displayed more potency against *AChE*. 4-Chloro-6-(4-(5-methyl-[1,2,4]triazolo [1,5-*a*]pyrimidin-7-yl)piperazin-1-yl)-*N*-(3-(trifluoromethyl)phenyl)-1,3,5-triazin-2-amine (**117**) showed the highest activity against *AChE* (IC_50_ = 0.065 ± 0.002 µM) and also modulated self-induced Aβ aggregation (75.32% ± 0.34%). Kinetic analysis revealed the interaction of this molecule with the peripheral anionic site of *AChE,* and *in silico* studies highlighted the drug-like properties of the derivatives (Jameel et al., 2017).


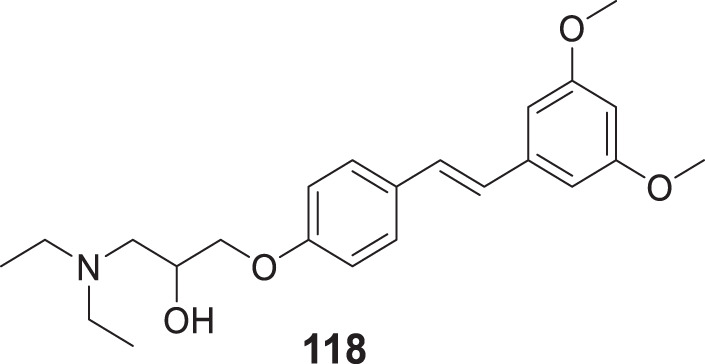


Zheng et al. reported the design and synthesis of pterostilbene β-aminoalcohols for AD treatment. These derivatives exhibited selective *AChE* inhibition; (*E*)-1-(diethylamino)-3-(4-(3,5-dimethoxystyryl)phenoxy)propan-2-ol (**118**) exhibited the higher inhibitory activity against *EeAChE* (IC_50_ = 24.04 ± 1.48 μM) than pterostilbene (less than 5.0% at a concentration of 50 μM). Moreover, compound **118** displayed good self-induced Aβ aggregation inhibition (40.23% ± 1.2%) with moderate antioxidant activity and neuroprotective effect (Zheng et al., 2018).


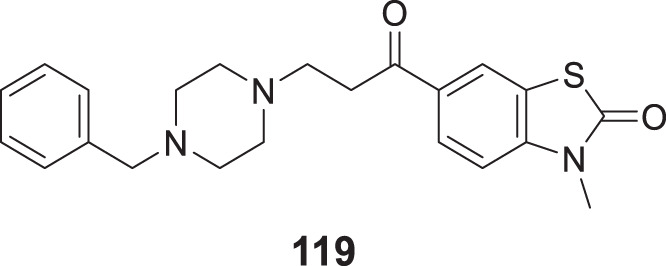


Erdogan et al. demonstrated the design and synthesis of four compounds with benzoxazolone and benzthiazolone cores as multifunctional agents against AD. Among the derivatives, 6-(3-(4-benzylpiperazin-1-yl)propanoyl)-3-methylbenzo [*d*]thiazol-2(3*H*)-one (**119**) bearing ketone group with benzothiazole core was the most potent analog. *In-vitro assay* indicated high *EeAChE* inhibition (IC_50_ = 0.34 ± 0.16 μM) with moderate anti-self-induced Aβ aggregation (57.5% ± 5.3%). SAR study revealed that the benzothiazole ring was most favorable for *AChE* and *BuChE* inhibition, and the ketone group is optimistic for *AChE* inhibition and the amide group for *BuChE* inhibition (Erdogan et al., 2021).


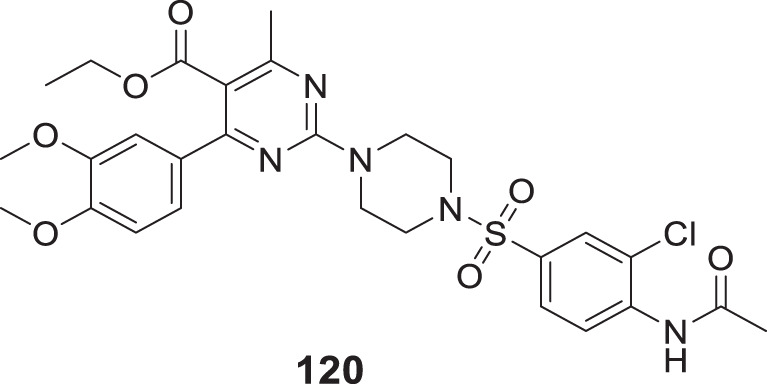


Manzoor et al. developed a series of phenyl sulfonyl-pyrimidine carboxylates for AD treatment. Ethyl 2-(4-((4-acetamido-3-chlorophenyl)sulfonyl)piperazin-1-yl)-4-(3,4-dimethoxyphenyl)-6-methylpyrimidine-5-carboxylate (**120**) among the derivatives displayed excellent inhibitory activity against *AChE* (IC_50_ = 47.33 ± 0.02 nM) over *BuChE* (IC_50_ = 159.43 ± 0.72 nM). Compound **120** also indicated more potent anti-aggregation activity (51.3%). Enzyme kinetics study revealed a non-competitive type of inhibition and good binding interaction with *AChE* active site than *BuChE* suggested by docking. Also, BBB permeability assay and *in silico* studies reported positive results (Manzoor et al., 2021).


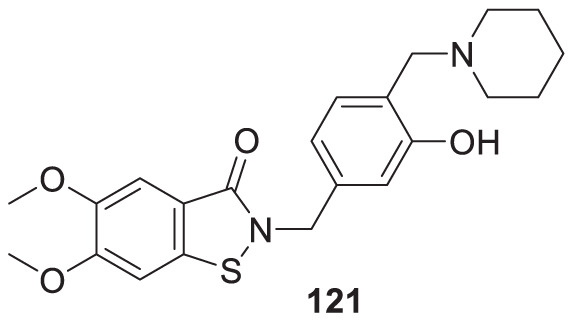


He et al. designed 2-(3-hydroxybenzyl)benzo [*d*]isothiazol-3-(2*H*)-ones Mannich bases against AD and demonstrated 2-(3-hydroxy-4-(piperidin-1-ylmethyl)benzyl)-5,6-dimethoxybenzo [d]isothiazol-3(2*H*)-one (**121**) to possess high *EeAChE* inhibitory activity (IC_50_ = 1.09 ± 0.02 μM) with moderate self-induced Aβ aggregation inhibition (25.0%). In addition, it was also depicted to have metal chelating ability, excellent neuroprotective effect in H_2_O_2_-induced PC12 cell injury, and good BBB permeability. Moreover, the step-down avoidance test demonstrated that compound **121** had improved scopolamine-induced memory deficit in mice (He et al., 2021).


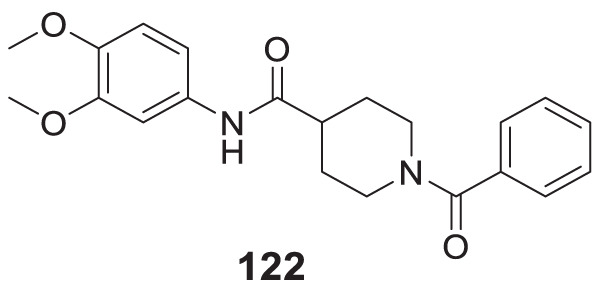


Gabr et al. developed donepezil analogues as MTDLs against Alzheimer’s disease. BACE-1 inhibition by these donepezil analogues was achieved by introducing amide linkers as the backbone capable of hydrogen-binding with the catalytic site of BACE-1. 1-Benzoyl-*N*-(3,4-dimethoxyphenyl)piperidine-4-carboxamide (**122**) emerged as the most active analogues with low nanomolar inhibition against both *hAChE* (IC_50_ = 4.11 ± 0.12 nM) and *β-secretase* (IC_50_ = 18.3 ± 0.17 nM) than donepezil. Moreover, compound **122** demonstrated metal chelating ability and low toxicity on SH-SY5Y neuroblastoma cells (Gabr and Abdel-Raziq, 2018).


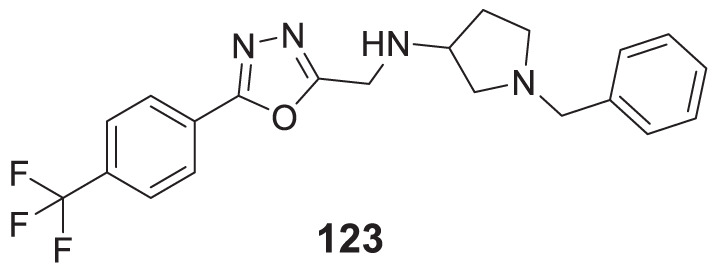


Choubey et al. developed novel *N*-benzyl pyrrolidine and 1,3,4-oxadiazole hybrids and evaluated them against *in-vitro* and *in-vivo* biological activities. Among the synthesized molecules, 1-benzyl-*N*-((5-(4-(trifluoromethyl)phenyl)-1,3,4-oxadiazol-2-yl)methyl) pyrrolidin-3- amine (**123**) displayed extensive inhibition against *hAChE* (IC_50_ = 0.064 ± 0.006 μM)*, hBuChE* (IC_50_ = 0.074 ± 0.016 μM), and *β-secretase* (IC_50_ = 0.143 ± 0.024 μM). Compound **123** has significant PAS site binding capability, BBB permeability, and neuroprotection ability on SHSY-5Y cell lines. The *ex-vivo* activity was executed on rat brains and demonstrated reduced *AChE* levels and oxidative stress (Choubey et al., 2021).


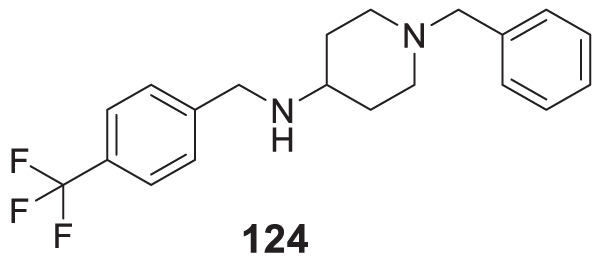


In separate work, Sharma et al. developed a series of *N*-benzylpiperidine analogues as multi-functional and tested them against Alzheimer’s disease biological targets. 1-Benzyl-*N*-(4-(trifluoromethyl)benzyl)piperidin-4-amine (**124**) exhibited excellent inhibitory activity towards *hAChE* (IC_50_ = 0.11 ± 0.02 μM) and *β-secretase* (IC_50_ = 0.22 ± 0.02 μM). Compound **124** displayed good interaction with the PAS site of *AChE*, and no detectable neurotoxicity was observed in SH-SY5Y neuroblastoma cell lines. Moreover, the active molecule ameliorated the scopolamine-induced cognitive impairment in elevated plus and Y-maze experiments (Sharma et al., 2019).


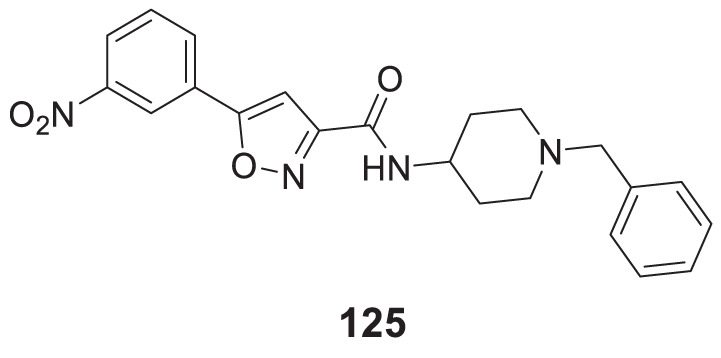


Similarly, Saeedi et al. explored the *N*-benzylpiperidine scaffold and designed a series of *N*-(1-benzylpiperidin-4-yl)-5-arylisoxazole-3-carboxamides and evaluated them as anti-Alzheimer’s agents, and compound **125** was identified as the best candidate with inhibition of *AChE* (IC_50_ = 16.07 ± 0.07 μM), *BuChE* (IC_50_ = 15.16 ± 0.22 μM) and BACE-1 (24.3%). A kinetic study indicated mixed-type inhibition for both enzymes and docking study revealed that *N*-(1-benzylpiperidin-4-yl)-5-(3-nitrophenyl)isoxazole-3-carboxamide (**125**) fitted well in the enzyme’s active site. Also, this active compound exhibited good metal chelating and neuroprotective activity (Saeedi et al., 2021).


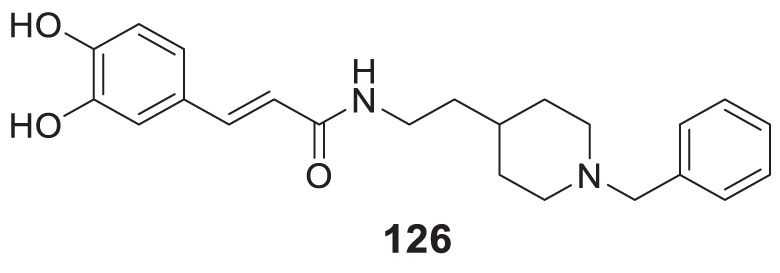


Estrada et al. designed a family of MTDL by linking antioxidant cinnamic-related structures with *N*-benzyl piperidine or *N,N*-dibenzyl (*N*-methyl)amine (DBMA) fragments. The resulting hybrids displayed a balanced biological profile. Among them, (*E*)-*N*-(2-(1-benzylpiperidin-4-yl)ethyl)-3-(3,4-dihydroxy phenyl)acrylamide (**126**) exhibited good activity against human *AChE* (IC_50_ = 1.75 ± 0.12 µM), *BuChE* (IC_50_ = 0.69 ± 0.12 µM), *MAO-A* (IC_50_ = 3.5 ± 0.2 µM) and *MAO-B* (IC_50_ = 6.0 ± 0.4 µM). SAR studies revealed that the presence of p-hydroxy groups in a cinnamic acid fragment was essential for getting inhibition and introducing the second hydroxyl at *ortho*- or *meta*-increased the inhibitory potency towards MAO. Compound **126** displayed a good neuroprotective effect against human neuroblastoma cell lines SH-SY5Y and also, a neurogenic effect by stimulating the differentiation of adult SGZ-derived neuronal stem cells (Estrada et al., 2016).


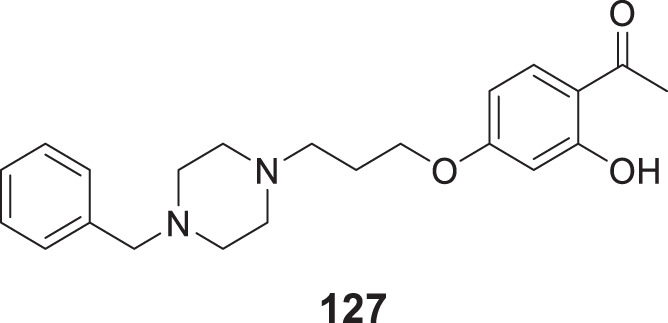


Sang et al. designed 2-acetyl-5-O-(amino-alkyl)phenols and evaluated them as multi-function inhibitors for treating Alzheimer’s disease. The results revealed that 1-(4-(3-(4-benzylpiperazin-1-yl)propoxy)-2-hydroxyphenyl)ethan-1-one (**127**) indicated selective *eeAChE* inhibitory potency (IC_50_ = 0.96 ± 0.01 µM) and high MAO-B inhibitory potency (IC_50_ = 6.8 ± 0.31 µM). Moreover, compound **127** acts as an antioxidant, neuroprotectant, and selective metal-chelating agent (Sang et al., 2017c).


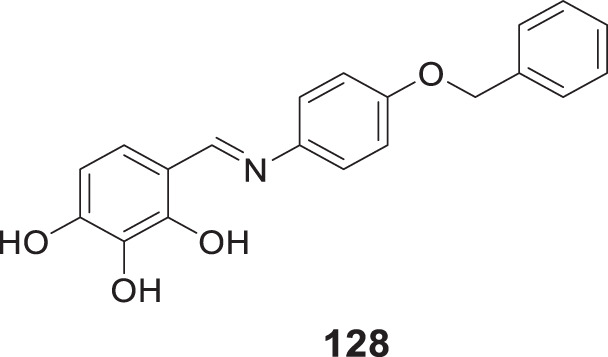


Yang et al. developed salicylaldimines as multi-target-directed ligands for treating Alzheimer’s disease. The biological evaluation identified (*E*)-4-(((4-(benzyloxy)phenyl)imino)methyl)benzene-1,2,3-triol (**128**) exhibiting excellent potency for inhibition of self-induced A*β* aggregation (91.3%) and human MAO-B (IC_50_ = 1.73 ± 0.39 µM). Moreover, compound **128** also displayed remarkable antioxidant, neuroprotective, and significant anti-inflammatory activity (Mezeiova et al., 2021).


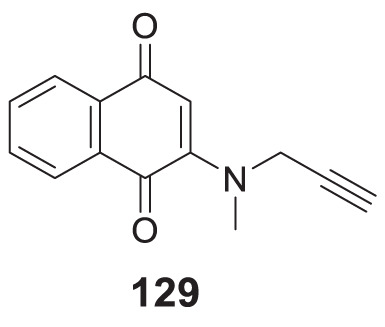


Mezeiova et al. designed novel 2-propargyl amino-naphthoquinones as an anti-Alzheimer agent. Among them, 2-(methyl (prop-2-yn-1-yl)amino)naphthalene-1,4-dione (**129**) exhibited good inhibitory activity against human MAO-A (IC_50_ = 6.64 ± 0.41 μM) and A*β* aggregation (67.4%). Further, compound **129** displayed a low toxicity and an anti-inflammatory profile in the lipopolysaccharide-stimulated cellular model (Mezeiova et al., 2021). See Supplementary Table 2 for a summary of dual-target inhibitors.

### 4.7 Multi-target inhibitors


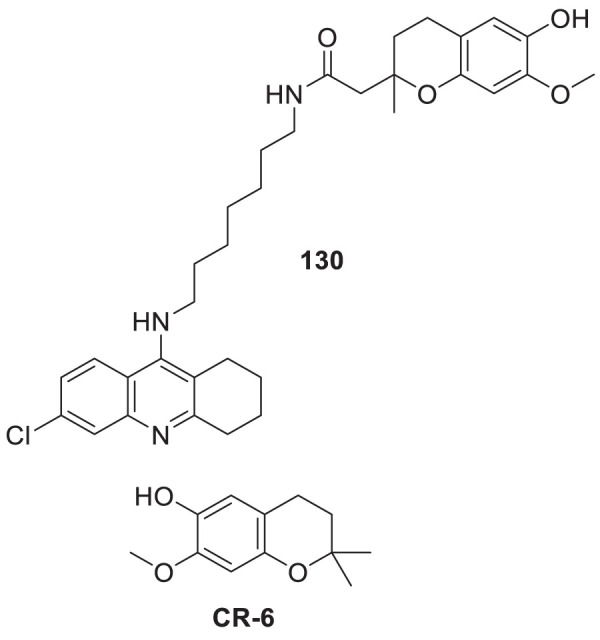


Perez-areales et al. designed, synthesized, and evaluated various derivatives from anti-oxidant lead CR-6 for anti-Alzheimer’s treatment. They have combined two structure backbones of 7-methoxy-2,2-dimethylchroman-6-ol (CR-6) with 6-chlorotacrine with a carbon chain linker. This hybrid molecule demonstrated multiple activities. From this, hybrid *N*-(8-((6-chloro-1,2,3,4-tetrahydroacridin-9-yl)amino)octyl)-2-(6-hydroxy-7-methoxy-2-methylchroman-2-yl) acetamide (**130**) indicated potent *in-vitro* and *in-vivo* biological activity. Such as *hAChE* inhibition with IC_50_ of 3.69 nM, *hBuChE* inhibition with IC_50_ of 170 nM, inhibition of DPPH with IC_50_ of 19.1 µM, inhibition of BACE-1with IC_50_ of 19.0% µM and Aβ42 percent aggregation inhibition <10, percent tau aggregation inhibition = 15 ± 2.1. *In-vivo* efficacy study in double transgenic APP/PS1 mice illustrated a positive tendency to improve cognition, amyloid pathology, and oxidative stress (Pérez-Areales et al., 2020).


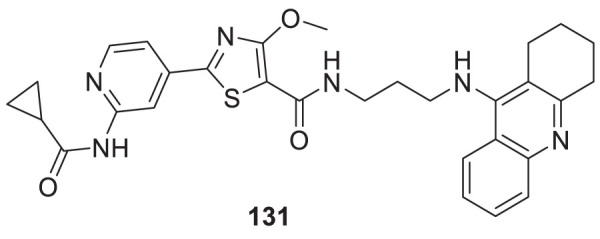


Jiang et al. designed multi-target-directed ligands as anti-Alzheimer’s agents wherein researchers combined GSK-3β-inhibitor and tacrine as *AChE* inhibitors via carbon linker, exhibiting multi-targeted activity. 2-(2-(Cyclopropanecarboxamido)pyridin-4-yl)-4-methoxy-*N*-(3-((1,2,3,4-tetrahydroacridin-9-yl)amino)propyl)thiazole-5-carboxamide (**131**) indicated the most potent activity from the series of synthesized compounds with *AChE* inhibition (IC_50_ = 6.5 nM) and hGSK-3β kinase activity (IC_50_ = 66 nM). *In-vivo* study revealed that compound **131** displayed less hepatotoxicity than tacrine. It also indicated potent inhibition of Aβ aggregation at 20 µM. Also, inhibition of tau protein hyperphosphorylation was studied by Western blot analysis at 30 µM (Jiang X. Y. et al., 2018).


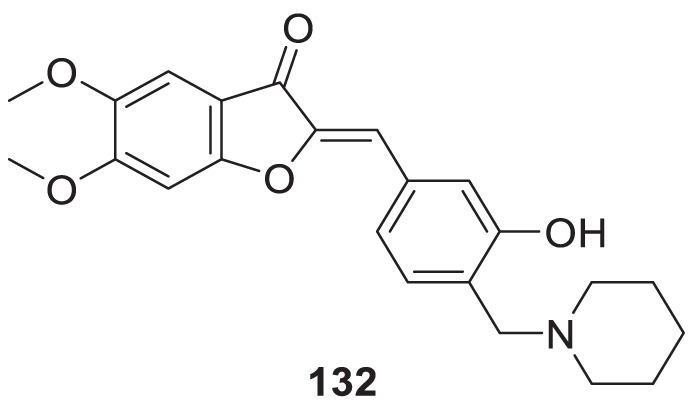


Li et al. designed and synthesized a series of aurone Mannich derivatives as multi-functional agents against Alzheimer’s disease. *In-vitro* assay demonstrated that derivatives are selective *AChE* inhibitors with multifunctional properties. (*Z*)-2-(3-Hydroxy-4-(piperidin-1-ylmethyl)benzylidene)-5,6-dimethoxybenzofuran-3(2*H*)-one (**132**) exhibited excellent activity against human *AChE* (IC_50_ = 0.0371 ± 0.004 µM), self-induced *β*-aggregation (58.1%), and moderate activity against MAO-B (32%). Moreover, compound **132** also displayed neuroprotective activity against H_2_O_2_-induced PC12 cell injury and high antioxidant properties (Li et al., 2017).


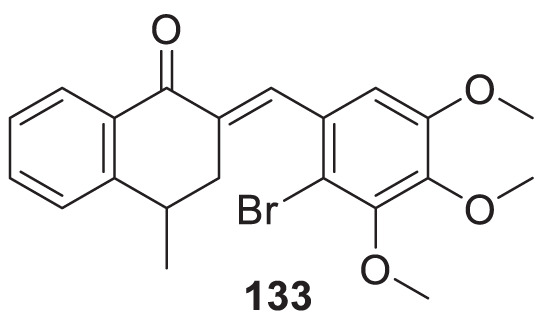


Leng et al. developed a series of α,β-unsaturated carbonyl-based tetralone derivatives against Alzheimer’s disease. *In-vitro* experiments revealed that (*E*)-2-(2-bromo-3,4,5-trimethoxybenzylidene)-4-methyl-3,4-dihydronaphthalen-1(2*H*)-one (**133**) displayed inhibitory activity against *AChE* (IC_50_ = 0.045 ± 0.02 µM), MAO-B (IC_50_ = 0.88 ± 0.12 µM), and self-induced *β*-aggregation (78%). Also, compound **133** displayed a neuroprotective effect against neuronal cell death in PC12 cells (Leng et al., 2016).


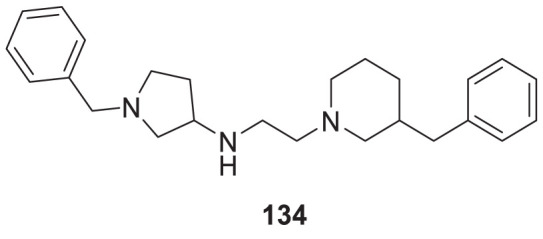


Wichur et al. designed 1-Benzylpyrrolidine-3-amine-based derivatives as novel multi-functional agents for treating Alzheimer’s disease. *In-vitro* studies suggested that 1-benzyl-*N*-(2-(3-benzylpiperidin-1-yl)ethyl)pyrrolidin-3-amine (**134**) efficiently inhibited *eqBuChE* (IC_50_ = 1.94 ± 0.02 μM), *β*-aggregation (49%), tau protein (54%), and BACE-1 (24%). Compound **134** also displayed radical scavenging activity and antioxidant activity (Wichur et al., 2020).


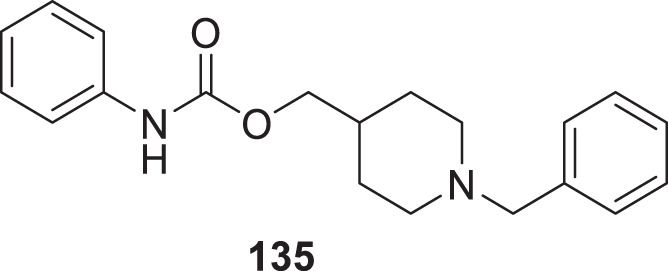


Kosak et al. developed a series of *N*-alkyl piperidine carbamates and evaluated them against *AChE*, *BuChE,* and MAO-B. Among them, (1-benzylpiperidin-4-yl)methyl phenylcarbamate (**135**) was the most promising compound demonstrating activity against *AChE* (IC_50_ = 7.31 μM), *BuChE* (IC_50_ = 0.56 μM), and MAO-B (IC_50_ = 26.1 μM). Enzyme kinetics experiments suggested compound **135** as a reversible and non-time-dependent inhibitor for *AChE* and *BuChE*. Further, compound **135** was not cytotoxic to human neuronal SH-SY5Y and liver HepG2 cells and prevented *β*-aggregation induced neuronal cell death (Košak et al., 2020).


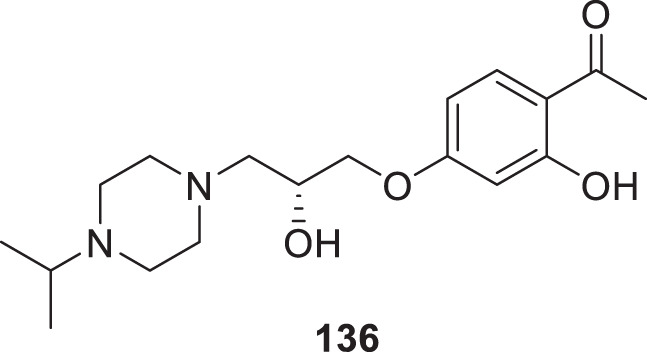


Zhu et al. designed a novel series of piperazine derivatives based on the MTDL strategy against Alzheimer’s disease. (*R*)-1-(2-Hydroxy-4-(2-hydroxy-3-(4-isopropylpiperazin-1-yl)propoxy) phenyl)ethan-1-one (**136**) was identified as an excellent multi-functional agent depicting activity against *eeAChE* (IC_50_ = 7.9 ± 0.26 μM), human MAO-B (IC_50_ = 9.9 ± 0.79 μM), and BACE-1 (IC_50_ = 8.3 ± 0.71 μM). Kinetics and molecular modeling study demonstrated that compound **136** had a mixed-type *AChE* inhibition and good interaction with CAS and PAS site of *AChE*. Also, compound **136** exhibited good antioxidant activity and neuroprotective effects (Zhu et al., 2021).


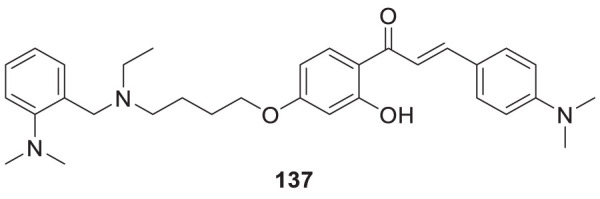


Sang et al. developed a novel series of dimethylamino chalcone-O-alkylamines derivatives as multifunctional agents for treating Alzheimer’s disease. Among the derivatives, (*E*)-1-(4-(4-((2-(dimethylamino)benzyl) (ethyl)amino)butoxy)-2-hydroxyphenyl)-3-(4-(dimethyl amino) phenyl)prop-2-en-1-one (**137**) displayed the greatest inhibitory activity against self-induced *β*-aggregation (IC_50_ = 0.88 ± 0.01 μM), *EeAChE* (IC_50_ = 0.69 ± 0.13 μM), and MAO-B (IC_50_ = 1.0 ± 0.02 μM). Molecular docking study and molecular dynamics simulations provided reasonable explanations for high efficiency. Also, compound **137** exhibited good antioxidant activity and neuroprotective effects (Sang et al., 2021).


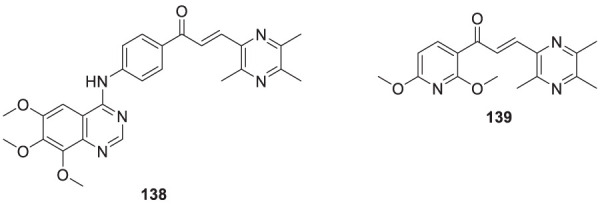


Wang et al. synthesized and evaluated chalcone derivatives for anti-Alzheimer activity. The introduction of tetramethylpyrazine to the structure of chalcone, a newer ligustrazine-based compound, was synthesized. These compounds exhibited various target inhibitions, revealing potent anti-Alzheimer activities. From this set of compounds, analog (*E*)-1-(4-((6,7,8-trimethoxyquinazolin-4-yl)amino)phenyl)-3-(3,5,6-trimethylpyrazin-2-yl)prop-2-en-1-one (**138**) demonstrated the inhibition of *AChE* with an IC_50_ value of 0.10 µM and *BuChE* with an IC_50_ value of 22.4 µM. However, this also inhibited MAO-A (IC_50_ = 47.4 µM) and MAO-B (IC_50_ = 2.6 µM). Similarly, (*E*)-1-(2,6-dimethoxypyridin-3-yl)-3-(3,5,6-trimethylpyrazin-2-yl)prop-2-en-1-one (**139**) displayed inhibition of *AChE* (IC_50_ = 0.025 µM), *BuChE* (IC_50_ = 2.7 µM), MAO-A (IC_50_ = 11.4 µM) and MAO-B (IC_50_ = 8.9 µM). In addition, compound **138** showed better neuroprotection as it is substituted with trimethoxyquinazoline amino moiety than pyrazinyl amino substitution. Moreover, the strong anticholinesterase activity of compound **139** was due to pyridin-3-yl-propanone moiety in the chalcone backbone (Wang et al., 2018).


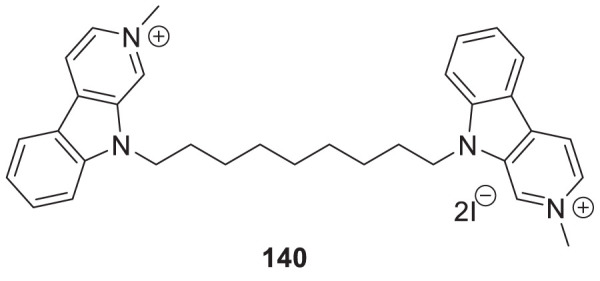


Rook et al. synthesized and evaluated a series of bivalent β-carbolines. These bivalent β-carbolines act on multiple targets such as *AChE*/*BuChE* enzyme and NMDA receptors. This affects both cholinergic and glutamate-induced excitotoxicity to improve treatment. In this series of synthesized agents, 9,9'-(nonane-1,9-diyl)bis (2-methyl-9H-pyrido [3,4-*b*]indol-2-ium) diiodide (**140**) demonstrated potent inhibition of *AChE* (IC_50_ = 0.5 nM) and *BuChE* (IC_50_ = 5.7 nM), whereas it inhibited NMDA receptors with IC_50_ of 1.4 µM. This bivalent compound has 1000-fold more activity than monovalent compounds. Spacer length in compound **140** should help solubility in cell culture, while more than nine carbon spacer molecules did not show activity. Methylation of second nitrogen gives a permanent positive charge to the compound, increasing activity (Rook et al., 2010).


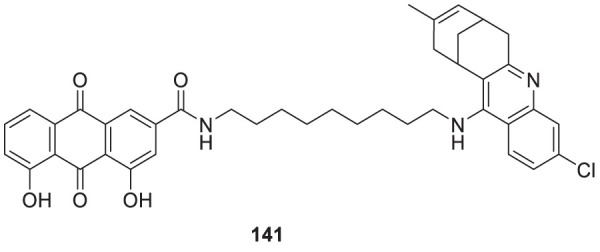


Viayna et al. synthesized multi-targeted disease-modifying anti-Alzheimer’s agents by combining rhein, huprine, and carbon chain spacer to make a hybrid molecule. Rhein and huprine Y are potent tau aggregation inhibitors that act individually. However, the hybrids of these two molecules inhibited cholinesterase and Aβ-42 aggregation. They have synthesized several racemic and enantiopure hybrid compounds of hydroxyanthraquinone drug rhein and connected to huprine Y via 5 to 11 methylene linkers. This exhibited that multi-targeted activity leads to dual binding inhibition of cholinesterase. Further, they inhibited Aβ aggregation and Tau aggregation inhibition. *N*-(9-((3-chloro-9-methyl-6,7,10,11-tetrahydro-7,11-methano cycloocta [*b*]quinolin-12-yl)amino)nonyl)-4,5-dihydroxy-9,10-dioxo-9,10-dihydroanthracene-2-carboxamide (**141**) showed potent *AChE* (IC_50_ = 2.39 nM) and *BuChE* inhibition (IC_50_ = 513 nM). In contrast, the *ex-vivo* activity of compound **141** in hippocampal slices of C57b16 mice proved that this hybrid molecule prevents the loss of synaptic proteins. Although an *in-vivo* study in transgenic APP-PS1 mice displayed that compound **141** can lower the level of hippocampal total soluble Aβ and reduce APP processing with potent BACE-1 inhibitory activity (IC_50_ = 80 nM) (Viayna et al., 2014).


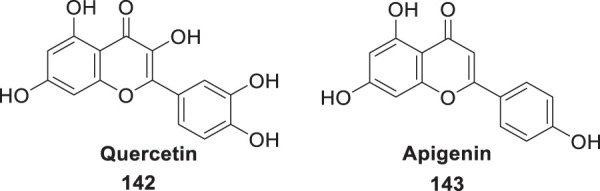


Espargaro et al. studied flavonoids and phenolic compounds combined *in-vitro* and *in silico* activity as anti-Alzheimer’s agents. Self-aggregation of Aβ peptide is a significant cause of Alzheimer’s disease. Researchers have studied various flavonoids for anti-Alzheimer’s activity by docking, molecular simulation, and Aβ aggregation assay. This study revealed that quercetin (2-(3,4-dihydroxyphenyl)-3,5,7-trihydroxy-4*H*-chromen-4-one, **142**) and apigenin (5,7-dihydroxy-2-(4-hydroxyphenyl)-4*H*-chromen-4-one, **143**) exhibited potent anti-aggregation property (Espargaró et al., 2017).


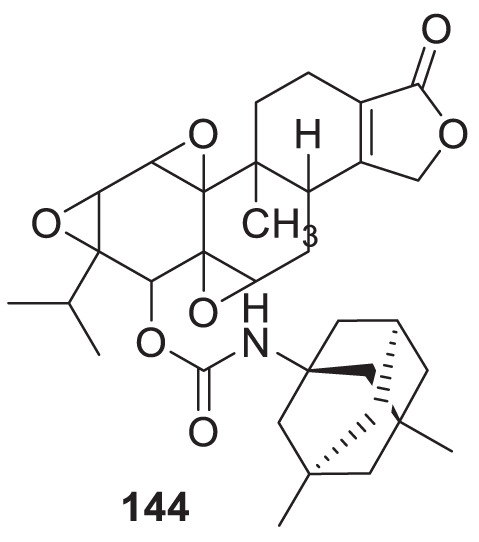


Ning et al. synthesized and studied the structure-activity relationship of triptolide derivatives for their multi-functional anti-Alzheimer activity. 8a-Isopropyl-10b-methyl-3-oxo-1,2,3,5,5b,6,6a,8,8a,9a,9b,10b-dodecahydrotris (oxireno)[2′,3′:4b,5; 2″,3″:6,7; 2‴,3‴:8a,9] phenanthro [1,2-*c*]furan-8-yl ((1*R*,3*R*,5*S*,7*R*)-3,5-dimethyladamantan-1-yl)carbamate **(144)** is a promising neuroprotective and anti Aβ aggregatory at sub-nanomolar concentration SAR studies revealed that the epoxy group is essential for neuroprotective activity (Ning et al., 2018).


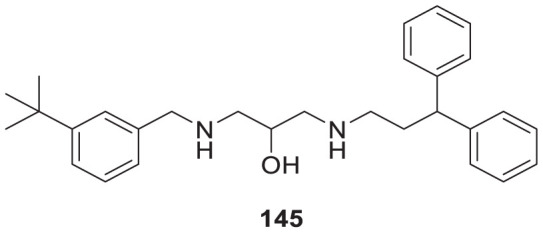


Panek et al. designed, synthesized, and evaluated a novel series of anti-Alzheimer agents. These 1-propane-1,3-diamine derivatives demonstrate multi-functional inhibition of various targets involved in Alzheimer’s disease. It showed activity against cholinesterase, β secretase, Aβ, and tau protein aggregation. From this series of compounds, 1-((3-(*tert*-butyl)benzyl)amino)-3-((3,3-diphenylpropyl)amino)propan-2-ol (**145**) displayed good activity with *BuChE* inhibition IC_50_ = 7.22 µM while inhibition of *β-secretase* with IC_50_ of 41.60 µM. Thioflavin-T assay exhibited inhibition of Aβ aggregation with IC_50_ value of 3.09 µM and tau aggregation inhibition with IC_50_ value of 44.4 µM (Panek et al., 2018).


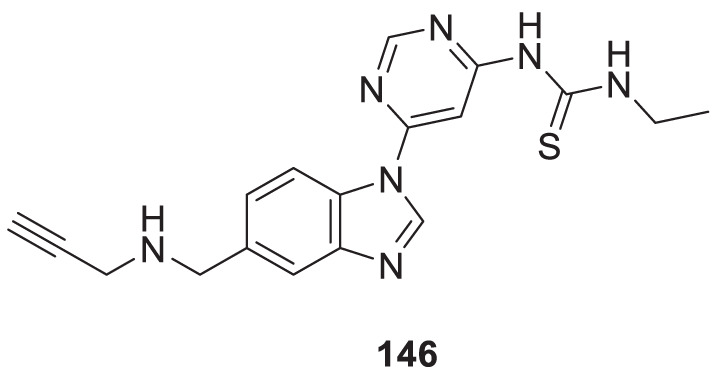


Xu et al. synthesized a novel series of propargylamine-modified pyrimidinylthiourea derivatives as multi-targeted anti-Alzheimer agents. 1-Ethyl-3-(6-(5-((prop-2-yn-1-ylamino) methyl)-1*H*-benzo [*d*]imidazol-1-yl)pyrimidin-4-yl)thiourea (**146**) from this series displays potent dual inhibition with *AChE* inhibition (IC_50_ = 0.032 µM) and MAO-B inhibition (IC_50_ = 2.117 µM). Additionally, compound **146** showed BBB permeability, antioxidant, and *in-vivo* copper chelating properties. An animal study on alleviating scopolamine-induced cognitive impairment in mice confirmed the inhibition of *AChE*/MAO-B activity. The presence of the benzimidazole ring in these compounds showed more potent inhibitory activity than the benzopyrazole substitution (Xu Y. et al., 2019).


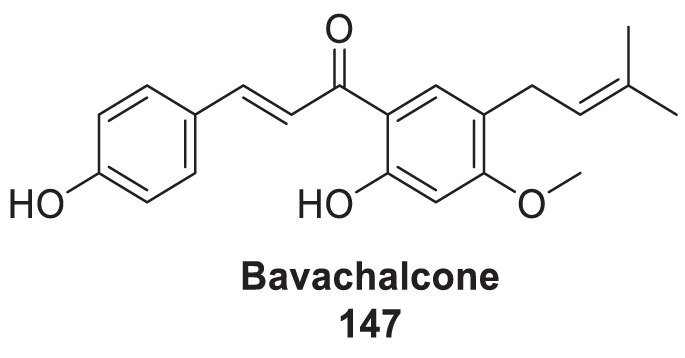


Xia Xu et al. reported the multi-targeted activity of prenylated compounds obtained from the *Psoralea fructus* plant, namely, bavachin, bavachinin, bavachalcone, and isobavachalcone. Bavachalcone ((*E*)-1-(2-hydroxy-4-methoxy-5-(3-methylbut-2-en-1-yl)phenyl)-3-(4-hydroxy phenyl)prop-2-en-1-one, **147**) displayed the most potent inhibition of Aβ42 aggregation, antioxidant activity, and *AChE* inhibition *in-vivo* and *in-vitro*. A docking study revealed that bavachalcone showed good binding interaction with the Aβ42 monomer (Xu et al., 2018).


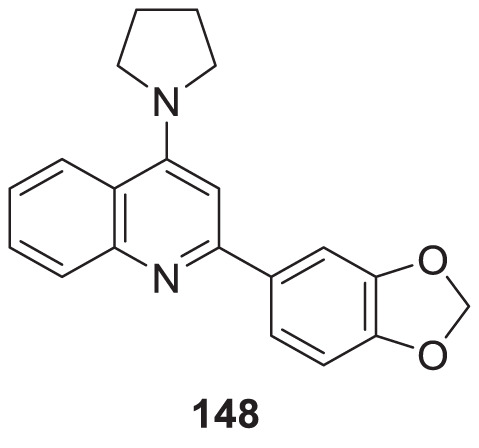


Luo et al. synthesized and evaluated novel graveolinine derivatives as anti-Alzheimer’s agents. From this series of derivatives, 2-(benzo[*d*][1,3]dioxol-5-yl)-4-(pyrrolidin-1-yl)quinoline (**148**) expressed the most potent activity with *AChE* inhibition (IC_50_ = 0.72 µM) and *BuChE* inhibition (IC_50_ = 0.16 µM). It also indicated potent self-induced Aβ aggregation inhibition (62.52%) studied on Thioflavin-T assay. A molecular docking study displayed that quinoline moiety establishes cation-pie interaction with amino acids, while nitrogen from pyrrolidine shows hydrogen bond interaction with the carbonyl group of amino acids (Luo et al., 2020).


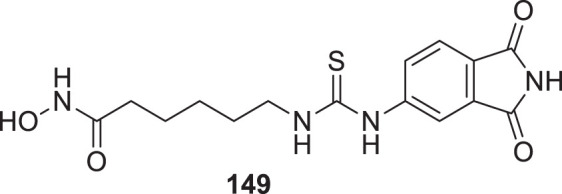


Simone et al. evaluated first-class GSK-3β/HDAC dual inhibitors as disease-modifying anti-Alzheimer’s agents. Histone deacetylase (HDAC) and glycogen synthase kinase 3β are vital targets in Alzheimer’s disease for drug discovery. These are designed by connecting hydroxamic acid with the phthalimide by carbon linker and thioamide functional group. From this series of dual inhibitors, 6-(3-(1,3-dioxoisoindolin-5-yl)thioureido)-*N*-hydroxy hexanamide (**149**) showed potent *in-vitro* activity with GSK-3β inhibition (IC_50_ = 2.69 μM) and inhibition of HDAC1 (IC_50_ = 12.78 μM), and HDAC6 (IC_50_ = 3.19 μM). According to a docking study, compound **149** indicated good H-bond interaction with active site residues of the enzymes. Moreover, compound **149** induced an increase in histone acetylation and reduced tau phosphorylation in *in-vitro* enzyme assays (De Simone et al., 2019).


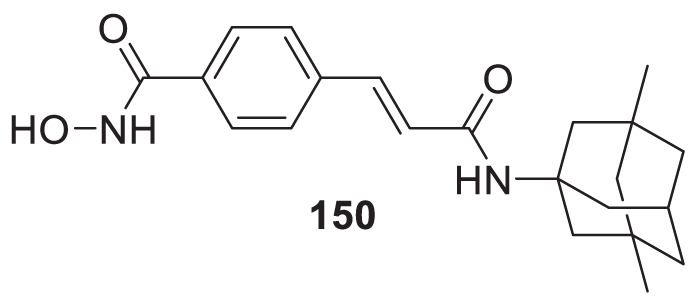


He et al. designed, synthesized, and evaluated hydroxamic acid-based compounds as a dual inhibitor of *N*-methyl-D-aspartase receptor (NMDAR) and HDAC for Alzheimer’s disease. As NMDA and histone deacetylase play important roles in neurodegenerative disorders, they are targeted for discovering novel anti-Alzheimer’s agents. A novel series of compounds were synthesized by linking aliphatic chains resembling a linker and aromatic chains, utilizing HDAC inhibitor SAHA and NMDAR inhibitor memantine as precursors. *In-vitro* HDAC inhibition and NMDAR study reveal that 4-((*E*)-3-(((1*S*,3*S*,7*S*)-3,5-dimethyladamantan-1-yl) amino)-3-oxoprop-1-en-1-yl)-*N*-hydroxybenzamide (**150**) exhibited potent inhibition of HDAC6 (IC_50_ = 0.18 μM) and of NMDAR (K_i_ = 0.59 μM). Further study confirmed a selective increase of the levels of HDAC6-directed substrate acetyl-tubulin in plasma. In addition to the neuroprotective properties, compound **150** was also less hepatotoxic (He et al., 2020).


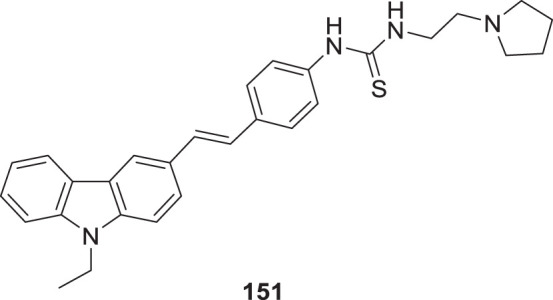


Patel et al. synthesized carbazole-stilbene hybrids as multi-targeted anti-Alzheimer agents. The fusion of carbazole with stilbene-designed hybrids showed activity against multiple targets, such as cholinesterase inhibition, Aβ aggregation inhibition, and anti-oxidant and metal chelating properties. From this hybrid, thiourea substituted (*E*)-1-(4-(2-(9-ethyl-9*H*-carbazol-3-yl)vinyl)phenyl)-3-(2-(pyrrolidin-1-yl)ethyl)thiourea (**151**) displayed potent activity with inhibition of *AChE* (IC_50_ value of 2.64 μM) and *BuChE* (IC_50_ value of 1.29 μM) and Aβ1–42 aggregation (51.29% at 25 μM concentration). It also possessed copper chelation properties. Docking study revealed that compound **151** exhibited strong covalent binding interaction with the active site of *AChE*, *BuChE*, and Aβ peptides (Patel et al., 2020).


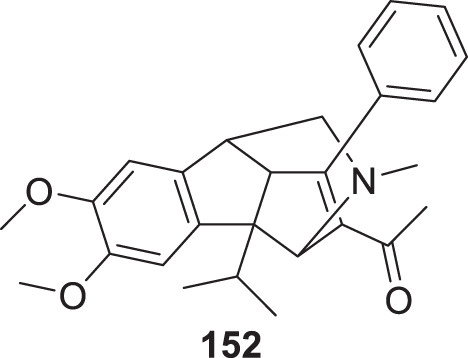


Titov et al. reported nitrogen-bridged cyclopenta [*a*]indenes as novel anti-Alzheimer’s agents. Several molecules were designed, synthesized, and evaluated for their biological activity. 1-(3a-Isopropyl-5,6-dimethoxy-10-methyl-1-phenyl-3,3a,8,8a-tetrahydro-3,8-(epiminomethano)cyclopenta [*a*]inden-2-yl)ethan-1-one (**152**) expressed potent inhibition of butyrylcholine esterase with IC_50_ of 0.034 µM and acetylcholine esterase with IC_50_ = 20.1 µM. It was influential in neuroprotection and also confirmed less cytotoxicity and good brain permeability. The docking study revealed that compound **152** exhibited hydrophobic interaction with *BuChE* binding site, presumably enhancing the inhibitory potency and selectivity over the *AChE* active site (Titov et al., 2021).


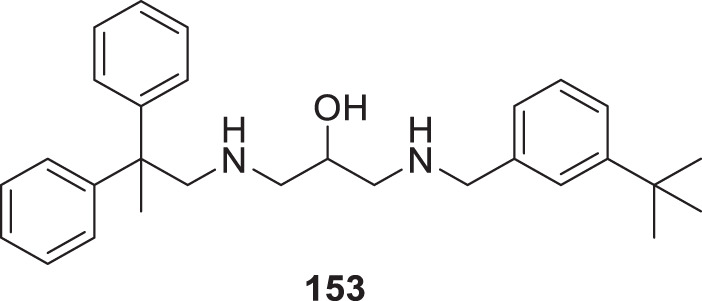


Pasieka et al. tested the library of 1-benzylamino-2-hydroxyalkyl as a multi-functional anti-Alzheimer’s agent and identified it as a dual aggregation inhibitor. From all the tested derivatives, 1-((3-(*t*-butyl)benzyl)amino)-3-((2,2-diphenylpropyl)amino)propan-2-ol (**153**) exhibited potent activity. *In-vitro* inhibition of *hBuChE* displayed IC_50_ of 5.74 μM, inhibition of *hBACE1*with IC_50_ of 41.6 μM and Aβ aggregation inhibition with IC_50_ = 3.09 μM). The docking study indicated hydrophobic interaction with amyloid (Pasieka et al., 2021). See Table 5 for a summary of multi-target inhibitors.

**TABLE 5:** Multi-target inhibitors.

Sr. No.	Compound	Activity	Assay type
130	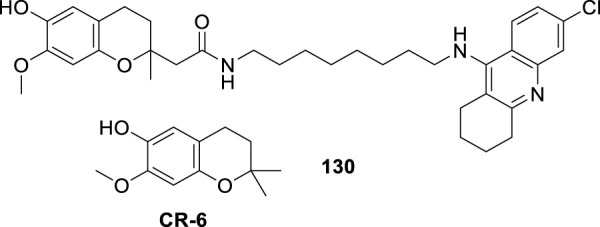	*hAChE* IC_50_ = 3.69 ± 0.19 nM *hBuChE* IC_50_ = 170 ± 9 nM DPPH IC_50_ = 19.1 ± 5.6 uM BACE-1 IC_50_ = 19.0 uM AB42 Aggregation % inhibition <10 Tau aggregation inhibition = 15 ± 2.1%	-Ellman assay -DPPH - fluorescence resonance energy transfer (FRET) assay -Aβ42 and Tau Aggregation Inhibition Assay
131	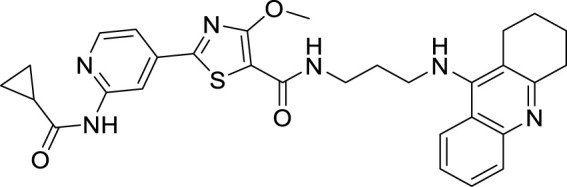	*hAChE* IC_50_ = 6.5 nM hGSK-3β kinase activity IC_50_ = 66 nM	-Ellman’s assay
132	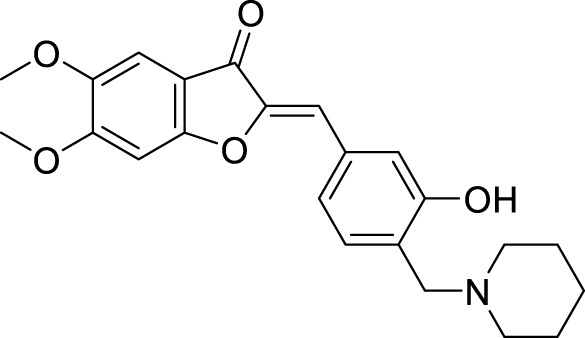	*- AChE* inhibition IC_50_ = 0.0371 ± 0.004 µM - anti-aggregation = 58.1% - MAO-B inhibition = 32%	*- in-vitro AChE* inhibition - Thioflavin-T assay - MAO inhibition assay
133	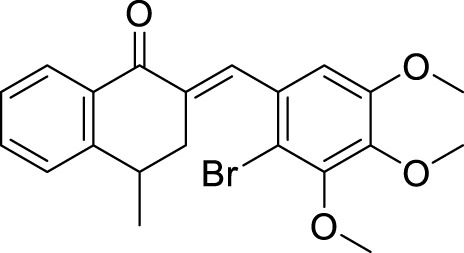	*- AChE* inhibition IC_50_ = 0.045 ± 0.02 µM - anti-aggregation = 78% - MAO-B inhibition IC_50_ = 0.88 ± 0.12 µM	*- in-vitro AChE* inhibition - Thioflavin-T assay - MAO inhibition assay
134	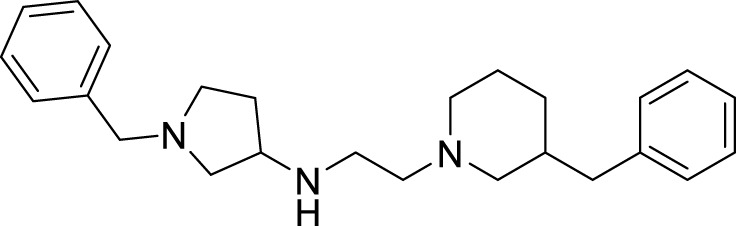	*- eqBuChE* inhibition IC_50_ = 1.94 ± 0.02 μM - anti-aggregation = 49% - BACE-1 inhibition = 24% - tau protein inhibition = 54%	*- in-vitro AChE* inhibition - Thioflavin-T assay - FRET assay
135	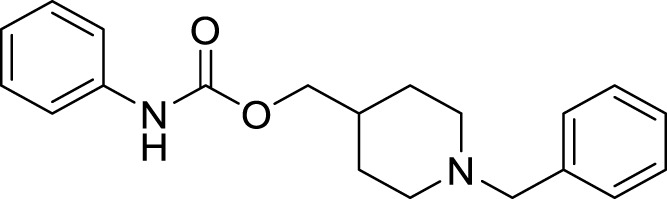	*- AChE* inhibition IC_50_ = 7.31 μM *- BuChE* inhibition IC_50_ = 0.56 μM - MAO-B inhibition IC_50_ = 26.1 μM	*- in-vitro AChE* inhibition - MAO inhibition assay
136	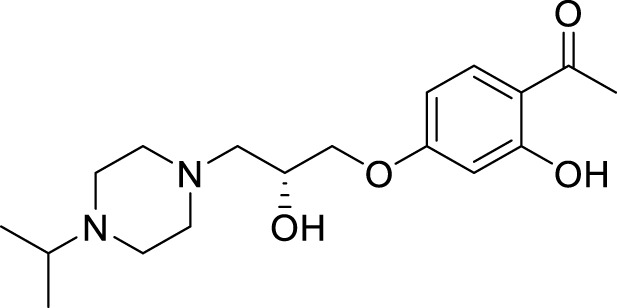	*- eeAChE* inhibition IC_50_ = 7.9 ± 0.26 μM - human MAO-B inhibition IC_50_ = 9.9 ± 0.79 μM - BACE-1 inhibition IC_50_ = 8.3 ± 0.71 μM	*- in-vitro AChE* inhibition - MAO inhibition assay - FRET assay
137	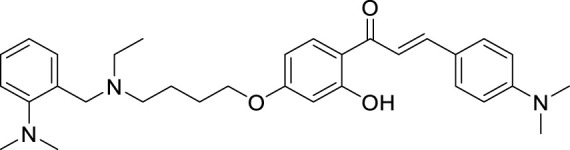	*- EeAChE* inhibition IC_50_ = 0.69 ± 0.13 μM - anti-aggregation IC_50_ = 0.88 ± 0.01 μM - MAO-B inhibition IC_50_ = 1.0 ± 0.02 μM	*- in-vitro AChE* inhibition - Thioflavin-T assay - MAO inhibition assay
138 139	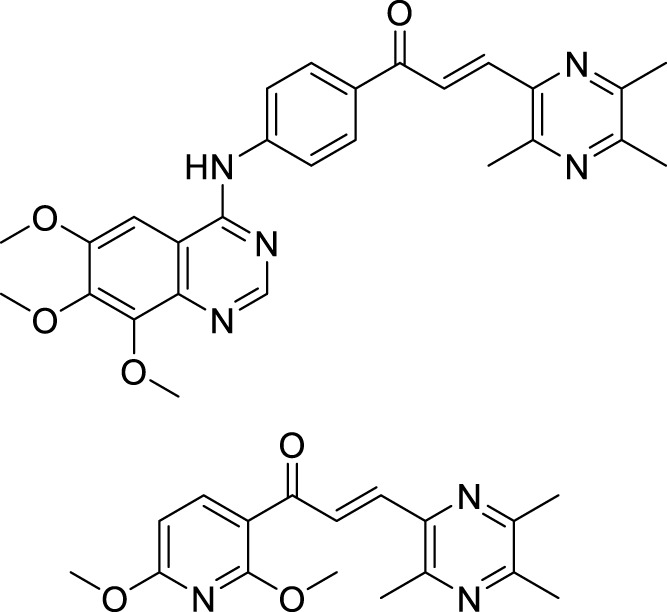	*AChE* IC_50_ = 0.025 ± 0.01 μM *BuChE* IC50 = 2.7 ± 1.4 μM MAO-A IC_50_ = 11.4 ± 2.1 μM MAO-B IC_50_ = 8.9 ± 1.7 μM	-Ellman’s assay -MAO-B inhibition assay
140	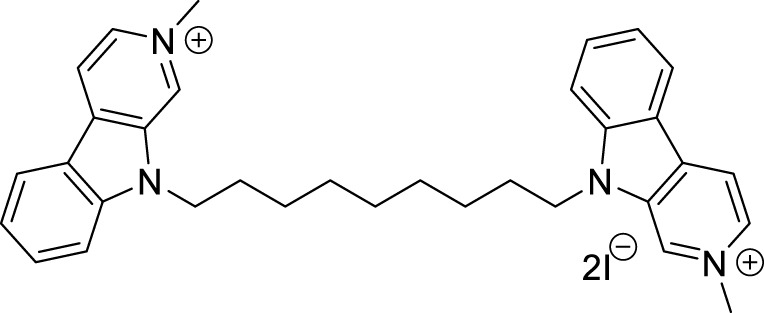	*AChE* IC50 = 0.5 nM *BuChE* IC_50_ = 5.7 nM NR inhibitionL12-G10 IC_50_ = 1.4 ± 0.2 μM L13-E6 IC_50_ = 2.9 ± 1.1 μM	-Ellmans assay -NMDA Receptor Inhibitory
141	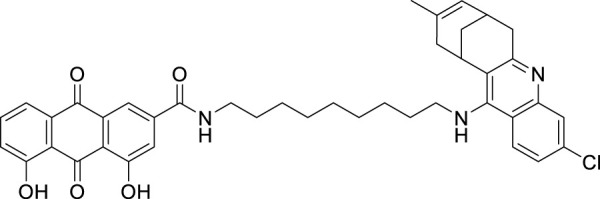	*hAChE* IC_50_ = 2.39 nM BACE-1 IC_50_ = 80 nM - anti-aggregation = 43%	-Ellman’s assay - ThT fluorescence assay
142 143	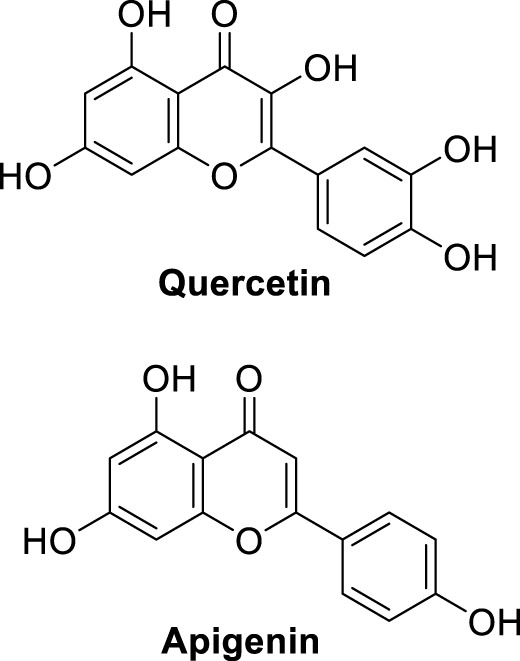	*-*	*-*
144	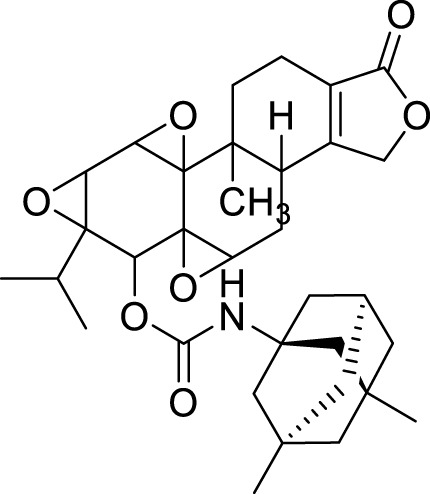	*-*	*-*
145	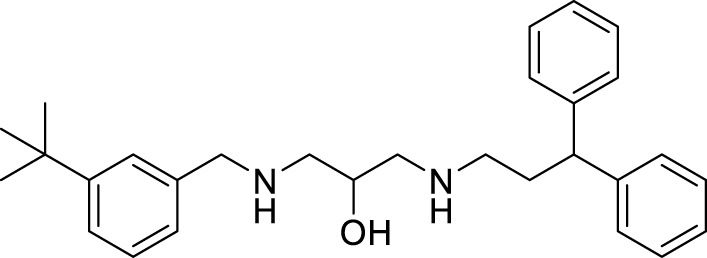	hBACE-1 IC_50_ = 41.60 μM Aβ-aggregation IC_50_ = 3.09 μM tau aggregation = 55% *hBuChE* IC_50_ = 7.22 μM	- Ellman’s assay
146	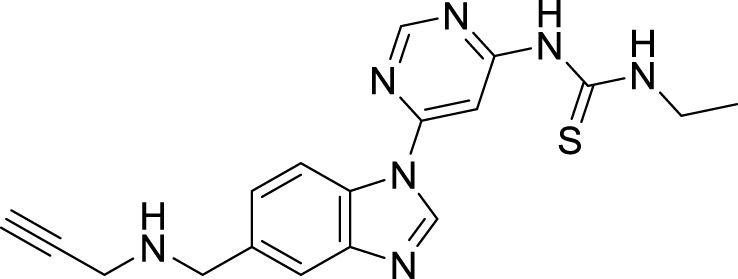	*AChE* IC_50_ = 0.032 ± 0.007 μM MAO-B IC_50_ = 2.117 ± 0.061 μM	-Ellman’s assay -MAO-A/B inhibition assay
147	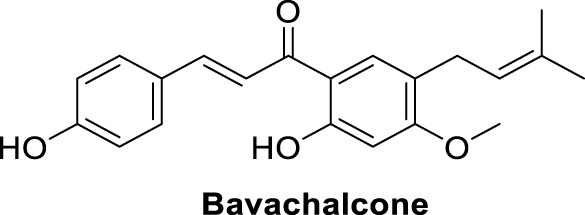	-	-*AChE* activity assays
148	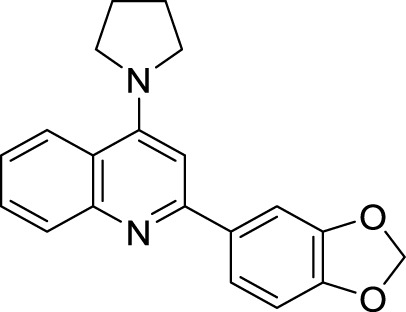	*AChE* IC_50_ = 0.72 μM BuChE IC_50_ = 0.16 μM Aβ inhibition = 62.52%	-Ellman assay -thioflavin-T (ThT) fluorescence
149	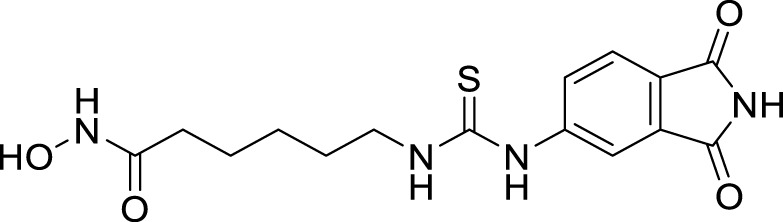	GSK-3β IC_50_ = 2.69 ± 0.01 μM HDAC1 IC_50_ = 12.78 ± 0.11 μM HDAC6 IC_50_ = 3.19 ± 0.08 μM	- *In-vitro* Enzymes Inhibition (GSK-3β, HDAC1, HDAC6)
150	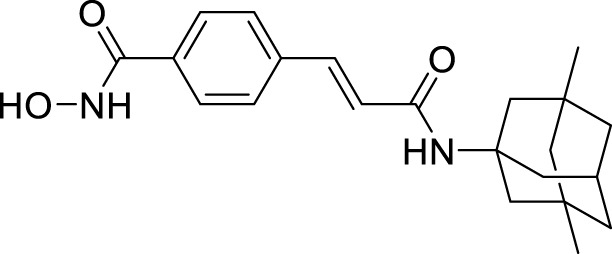	HDAC6 IC_50_ = 0.18 μM NMDAR Ki = 0.59 μM	-*In-vitro* HDACs inhibition -*In-vitro* activities against NMDAR
151	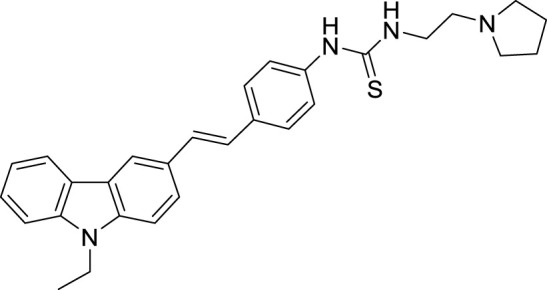	*AChE* IC_50_ = 2.64 μM *BuChE* IC_50_ = 1.29 μM Aβ1–42 aggregation = 51.29%	-Ellman’s assay - Thioflavin T (ThT) fluorescence assay
152	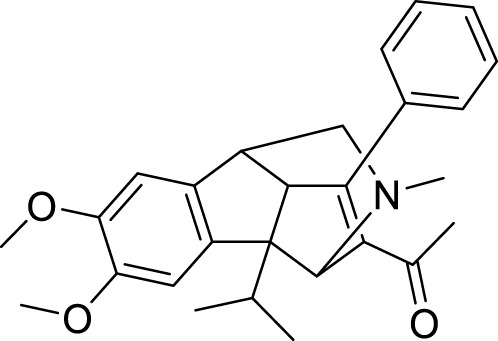	*hAChE* IC_50_ = 20.1 ± 3.4 μM *hBuChE* IC_50_ = 0.034 ± 0.002 μM	-Ellman’s assay - ThT fluorescence assay
153	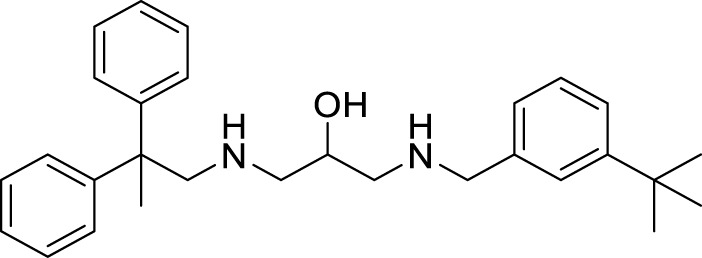	*hBuChE* IC_50_ = 5.74 μM *h*BACE1 IC_50_ = 41.6 μM -Aβ aggregation inhibition IC_50_ = 3.09 μM	-*in cellulo* thioflavin S (ThS) assay -PAMPA assay

#### 4.7.1 Recent representative SAR developments


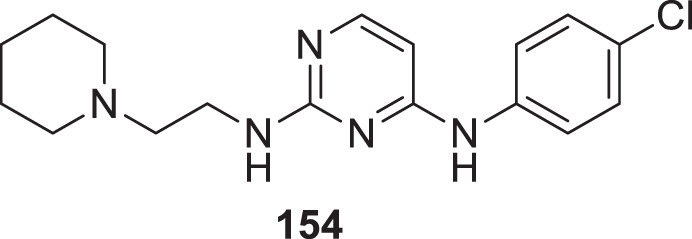


Pant et al. have developed and synthesized a few substituted pyrimidine derivatives and assessed their potential to prevent Alzheimer’s disease. Compound **154** [*N* ^4^-(4-chlorophenyl)-*N* ^2^-(2-(piperidin-1-yl)ethyl)-pyrimidine-2,4-diamine] (% *AChE* inhibition = 40.03 ± 0.04%), one of the synthesized derivatives, showed a superior anti-Alzheimer profile compared to donepezil (% *AChE* inhibition = 19.23 ± 0.05%). Compound **154** showed better neuroprotection suggesting the importance of incorporating *chloro* substitution at the C-4 position and piperidine-substituted secondary amine at the C-2 position indicating better *AChE* inhibitory potency. Molecular docking studies also showed that the phenyl ring-bearing *chloro* substitution at the C-4 position of **154** showed a hydrophobic interaction with the part of the active site of *AChE* consisting of the *PAS*. Further, *in silico* molecular property predictions indicated that all the new compounds have favorable drug-likeness and ADME properties for CNS activity (Pant et al., 2024).


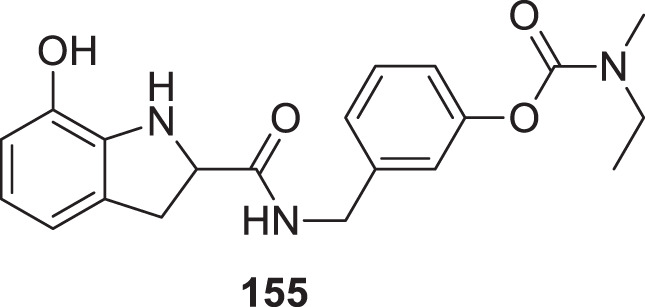


Bon et al. following a multitarget approach, synthesized and evaluated nine rivastigmine indole hybrids for multiple biological properties. Compound **155** 3-((7-hydroxyindoline-2-carboxamido)methyl)phenyl ethyl (methyl)carbamate (IC_50_ = 10.9 ± 0.1 μM) revealed higher *AChE* inhibition than the parent Rivastigmine drug (IC_50_ = 32.1 μM) and compound **155** displayed moderate *BuChE* inhibition (IC_50_ = 10.4 ± 0.4 μM) as compared to rivastigmine’s IC_50_ value of 0.39 μM and a hydroxyl substituent in the indole moiety demonstrated good antioxidant activity (EC_50_ = 14.5 ± 0.5 μM) (Bon et al., 2024).


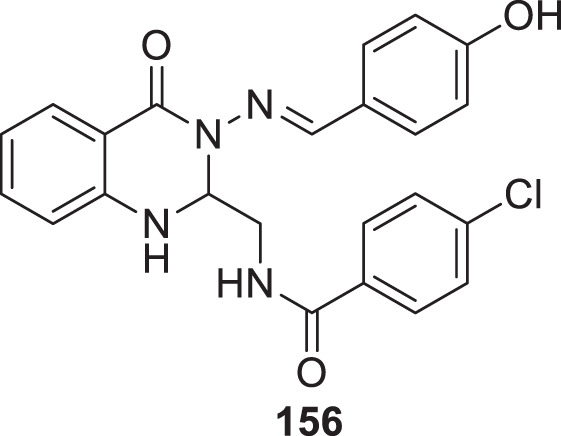


Moftah et al. designed and synthesized a series of quinazolinone-based derivatives as novel, multifunctional anti-AD drugs that exhibit both cholinesterase inhibitory and anti-inflammatory properties. Among the evaluated derivatives, compound **156** ((*E*)-4-chloro-*N*-((3-((4-hydroxybenzylidene)amino)-4-oxo-1,2,3,4-tetrahydroquinazolin-2-yl)methyl)benzamide) found to be potential compound showing anti-acetylcholinesterase (% *AChE* inhibition = 74.54 ± 2.53 at 100 μM), anti-inflammatory (23% reduction in TNF-α) and antioxidant activities. Compound **156** showed the general binding pattern in the *AChE* active site with its tetrahydroquinazolinone moiety in the peripheral active site (Moftah et al., 2024).


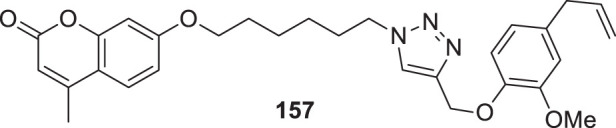


Singh et al. developed a series of triazole-tethered coumarin–eugenol hybrid molecules as potential multifunctional anti-Alzheimer’s agents using donepezil and a template. Among them compound **157** (7-((6-(4-((4-allyl-2-methoxyphenoxy)methyl)-1*H*-1,2,3-triazol-1-yl)hexyl)oxy)-4-methyl-2*H*-chromen-2-one) emerged as a selective *AChE* inhibitor (IC_50_ = 0.047 ± 0.008 μM) over *BuChE* (IC_50_ = ≥ 10 μM) with desired inhibition of Aβ aggregates (% inhibition = 72.21 ± 3.28 at 50 μM). Furthermore, **157** demonstrated protective properties against hydroxyl radicals, and simulation and molecular docking investigations validated the advantageous interactions between AChE and the Aβ monomer intended to suppress them (Singh et al., 2025).


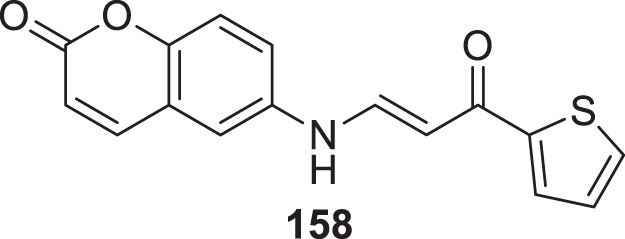


Abd El-Mageed et al. synthesized a novel series of coumarin derivatives as multi-target directed ligands (MTDLs) and assessed their anti-Alzheimer activity. Compound **158** ((*E*)-6-((3-oxo-3-(thiophen-2-yl)prop-1-en-1-yl)amino)-2*H*-chromen-2-one) showed outstanding activity as *hAChE* inhibition (IC_50_ = 26.03 ± 3.99 nM) and demonstrated good inhibitory activity against *hBuChE* (IC_50_ = 90.02 ± 6.71 nM) than donepezil (*hAChE* IC_50_ = 31.54 ± 2.16 nM and *hBuChE* IC_50_ = 614.50 ± 7.30 nM inhibition). Additionally, compound **158** demonstrated low cytotoxicity and inhibited the aggregation of tau protein (IC_50_ = 56.31 ± 3.43 μM) and A_β_ (IC_50_ = 35.04 ± 1.64 μM). According to kinetic and docking studies, compound **158** inhibited *hAChE* in a mixed way (Abd El-Mageed et al., 2025). See Table 6 for a summary of recent representative SAR developments.

**TABLE 6:** Recent representative SAR developments.

Sr.No.	Compound	Activity	Assay type
154	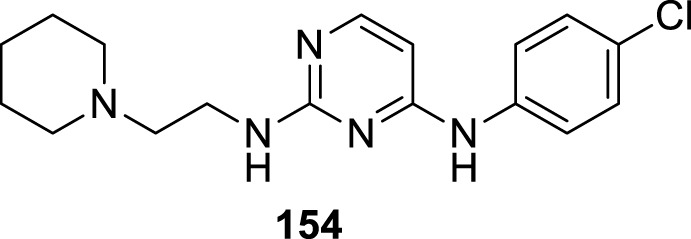	% *AChE* inhibition = 40.03 ± 0.04%	-Ellman’s assay
155	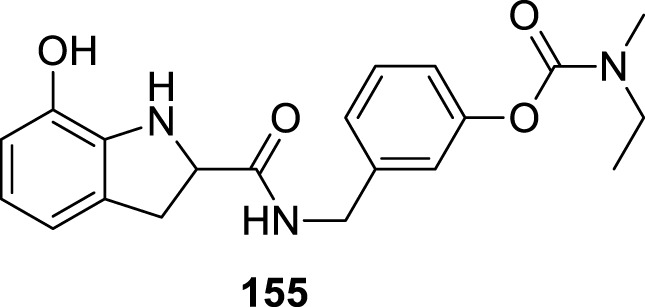	IC_50_ = 10.9 ± 0.1 μM)	*in-vitro AChE* inhibition
156	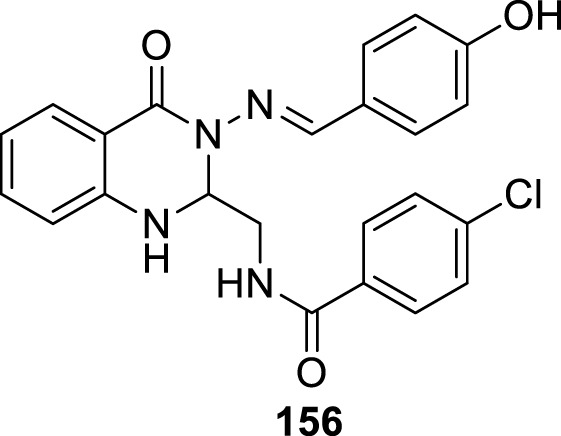	% *AChE* inhibition = 74.54 ± 2.53 at 100 μM	Ellman’s assay
157	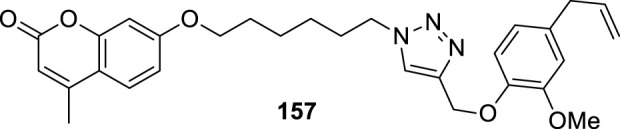	*AChE* inhibitor IC_50_ = 0.047 ± 0.008 μM *BuChE* IC_50_ = ≥ 10 μM	*- in-vitro AChE* inhibition - MAO inhibition assay
158	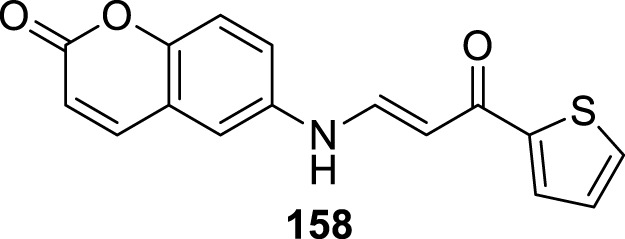	*hAChE* inhibition IC_50_ = 26.03 ± 3.99 nM	*- in-vitro AChE* inhibition

## 5 Discussion and perspectives

The goal of improving the activity of anti-Alzheimer’s disease therapeutics can be achieved by building off the current portfolio of available agents. On one hand it is very exciting that several first-generation drugs are now available for AD treatment. At the same time their limitations highlight the need to evolve molecules that satisfy required therapeutic efficacy devoid of side effects. Therefore, we perused the literature to identify formidable lead molecules available for Alzheimer’s disease and extensively scrutinized their pharmacology from a medicinal chemist viewpoint. To this end, we are hopeful that our assessment of empirical studies will amplify development of novel molecules for AD treatment.

Although it would be premature to predict the outcome of the therapeutic properties based on these early preliminary studies, these would help in prioritizing the additional structure activity relationship and further drug design efforts. Compounds **4**, **5**, and **10** showed moderate to full blood brain barrier permeability. It would be interesting to see further drug design efforts to enhance desired properties and by masking unwanted side effects (Camps et al., 2009; Hepnarova et al., 2018).

We began our evaluation with the most explored AD drug candidates, cholinesterase enzyme inhibitors, where tacrine is the prototypic marketed drug representing this class of compounds. However, due to its hepatotoxic nature, various molecules have been developed and studied to minimize or nullify off-target issues and to enhance anti-cholinesterase activity. Thus, tacrine remains the reference drug with its amino group at the ninth position that additionally provides target modifications at the primary amine position for the synthesis of analogues necessary to explore structure-activity relationships (SAR). These SAR studies also visit the second purpose that is to abolish or lessen the hepatotoxic effects of tacrine. Compound **3** exhibited no hepatotoxicity because of the incorporation of the NO donor structural component in the tacrine (Fang et al., 2008). Chromenotacrines such as compound **5** were less hepatotoxic as compared to tacrine. These chromenotacrines had 3,4,5-trimethoxyphenyl at the 4-position of the chromeno ring in addition to a 7-hydroxy moiety. Chromeno was fused with 4-aminotetrahydroquinoline (Oset-Gasque et al., 2014). Less hepatotoxicity is associated with adamantanyl thiourea derivative (Spilovska Chalupova et al., 2013). In a separate study, it was observed that fused substituted chromenopyridone synthesized compounds, **14** and analogs, had no hepatotoxicity (Machaa et al., 2021). It was interesting to observe that the clubbing of tacrine with GSK-3β inhibitor (compound 131 and analogs) led to a reduction in the hepatotoxicity of tacrine (Jiang X. Y. et al., 2018).

When the hydrogen of the amino group is replaced with three carbon chains, the resulting analog exhibits favorable interactions with the enzyme. In contrast, additional carbons placed on the chain lead to subsequently decreased efficacy. Most of the reported novel molecules bear amide groups as a linker, and substitutions such as a quinolone ring resulted in beneficial effects towards *AChE* inhibition. Replacement of the carbon chain with a five-membered heterocyclic ring increased the activity towards cholinesterase inhibition whereas substitutions to the tacrine’s aromatic ring enhanced selectivity towards acetylcholinesterase.

It is observed that compound **6** with chloro substitution on the acridine ring favourably attaches to *AChE* over *BuChE*. Additionally, similar trends are also observed for other pairs of synthesized compounds containing choro-substituted and unsubstituted by Najafi et al. (2017) Similarly, studies related to compound **85** and analogs proved that the presence of chloro affects *AChE* binding positively (Hiremathad et al., 2018b). In the modified tacrine, substituted pyrano [2,3-*b*]-quinoline (compound **35** and related compounds), substitution of electron-withdrawing groups on the phenyl ring present at the fourth position of the pyrano ring favoured cholinesterase inhibition (Eghtedari et al., 2017). Likewise, in another modified tacrine, substituted fused pyrazolo [1,2-*b*] phthalazines in the compound **113** and analogs have preference for the *AChE* binding over *BuChE* (Jalili-Baleh et al., 2017).

Chromene scaffolds have also been examined for anti-cholinesterase activity. Novel molecules were developed by fusing the chromene ring with coumarin **11** and carboxamido-alkylamines **12**. Although compound **12** exhibited better *AChE* inhibition than tacrine, compounds **11** and **12** were less effective as cholinesterase inhibitors than other tacrine derivatives, offering moderate binding affinity to catalytic active site (CAS) and peripheral anionic site (PAS). In contrast, molecules with fused quinoline rings (**14**) were more active due to greater interaction with the cholinesterase enzyme CAS and PAS sites. Similarly, coumarin derivatives were evaluated as cholinesterase inhibitors; as mentioned above, introducing substitution in the coumarin ring displayed higher activity, and aromatic moiety **16** was more favorable than the heterocyclic ring.

Pyridine is the six-membered heterocycle found in various marketed drugs as an essential structural moiety. The pyridine derivatives have also been evaluated for Alzheimer’s treatment. A hybrid molecule involving a carbazole ring with benzyl and pyridine **22** showed selectivity towards BuChE. A modified donepezil benzyl piperidine moiety with pyridinium structure **30** with the quaternary nitrogen fits well in the oxyanion hole of the AChE active site and, therefore, demonstrated AChE inhibition activity with increased affinity towards CAS and PAS. Moreover, substituted pyrimidine derivatives **154** demonstrated improved neuroprotection and good AChE inhibitory activity, indicating the significance of adding chloro substitution at the C-4 position and piperidine-substituted secondary amine at the C-2 position. In comparison, the phenyl ring with chloro substitution at the C-4 position of 154 showed hydrophobic interaction with the part of the active site of *AChE* consisting of the PAS. Chiral 1,4-dihydropyridine derivatives **48** have also emerged with excellent selectivity towards AChE inhibition. A known caveat is that 1, 4-dihydropyridines are also calcium channel blockers that serve as antihypertensive agents and non-CNS activity would be an important consideration. Yet, this also provides opportunity to examine structure activity relationships and perhaps develop new analogs with CNS specificity. Alternatively, these compounds may exhibit dual benefits (i.e., anti-AD and antihypertensive), as both AD and hypertension can coexist with older age.

Phenolic compounds have been reported for various pharmacological actions, with xanthone representing one such phenolic derivative. The xanthone skeleton contributes to a hydrophobic binding interaction with the active site of the cholinesterase enzyme, thereby improving inhibition activity. Considering xanthones pharmacological importance, derivatives of xanthones against the AChE have been explored extensively. The novel 3-O-substituted xanthone **37** and xanthone alkyl benzylamine variants **38** derivatives have an alkyl chain as a linker, which results in potent inhibition of the AChE. The optimal chain length for AChE inhibition was found to be with four carbons, while increasing chain length results in dual selectivity towards AChE and BuChE. Xanthone derivatives possess additional pharmacological activities including antidiabetic, anticancer, antibacterial, antifungal, and antimalarial properties. Therefore, value will be added in understanding how synthesized xanthone analogs reflect activity in these realms.

Molecules containing several heterocyclic rings have also been explored as cholinesterase inhibitors. For example, oxadiazole, a combination of triazole and naphthalene **31**, pyridoxine **36**, piperidine-4-one **33**, benzimidazole **85** and quinoline with phenyl and pyrano moiety containing derivatives **35**. Among them, the molecules containing quinoline-3-carboxylates with pyrano moiety at the fourth position and phenyl ring having an electron-withdrawing group at meta positions showed the most potent cholinesterase inhibition. Hydrazone derivatives **41** with amino and nitrobenzothiazole exhibited six times greater potency than donepezil and tacrine. The same compound exhibited mixed-type inhibition on AChE with an excellent docking score and additional radical scavenging properties.

Indole derivatives have also been evaluated for cholinesterase inhibition. The triazaindole derivatives **51**, having the triazine ring fused with an indole five-membered ring and additional amine linkage at the third position of the triazine ring with further carbon chain linkage. The derivatives were found to have potent activity against the cholinesterase enzyme. The indoloquinoxaline derivatives **53** displayed more selectivity towards BuChE over AChE. Rivastigmine indole hybrids **155** were also reported as a multitarget approach; these hybrids have dual enzyme inhibition function and higher *AChE* inhibition with equivalent *BuChE* inhibition. Moreover, the hydroxyl substituent in the indole moiety increased antioxidant activity. The hybrids drug-likeness was assessed *in silico*, and the results indicated that it appeared **155** to have potential oral availability. Indanone scaffolds were modified, and the benzyl moiety with mono- and dimethoxy substitution on benzene at nitrogen atoms **54** and **55** exhibited more potency towards cholinesterase inhibition than the indole derivatives mentioned above. Finally, molecules **57**, **58** and **59** with metalloid properties have been developed by utilizing selenium chemistry to modulate enzymatic activity. However, the presence of a selenium element resulted in lower potency against the cholinesterase.

Drugs such as eplerenone (**60**) and edaravone (**42**) exerted relatively poorer inhibitory activity toward cholinesterase enzymes. As the synthesis of these larger complex molecules required significant, challenging, and time-consuming efforts, extraction and purification of the active compounds from plants have been explored. Various notable plant extracts include bromophenols **52**, huperazine A (**63**), flavone-8-acrylamides **21**, and claulansine-F-donepezil hybrids **23**. These compounds along with the development of hybrid scaffolds, for inhibitory enzymatic activity have been evaluated. Among them, huperazine A showed higher potency, whereas the bromophenols displayed lower potency. Finally, the hybrid molecules **(81)** displayed a portfolio of higher activity and higher selectivity towards *AChE* than *BuChE*. It is noteworthy that natural products and their inspired derivatives have provided major drug molecules for many diseases and disorders. Given the integral role of natural products in drug design, lead molecules from this category have promise for future development.

In addition, numerous small molecules have been synthesized as potential inhibitors of Aβ aggregates. AB aggregation inhibitors aim to prevent or reduce the formation of amyloid β (Aβ) plaques, which are implicated in Alzheimer’s disease. This approach holds promise because it targets the early stages of plaque formation, potentially slowing or halting disease progression. However, the effectiveness of these inhibitors has been variable, and challenges include ensuring that the inhibitors reach their target in the brain and minimizing potential side effects. On the clinical side, earlier disease diagnosis would complement therapeutic efficacy. Therefore, ongoing research is focused on optimizing such inhibitors to improve their efficacy and safety. Among these, multipotent derivatives such as 2-piperidone **65** effectively prevented the dimerization of Aβ peptides. Additionally, compounds such as the dimer of 4-hydroxy carbazole-8-aminoquinoline and the selenium complex of quinolone have demonstrated the ability to inhibit Cu (II)-induced Aβ aggregation while showcasing antioxidant properties. Notably, triazine derivatives **68** have exhibited neuroprotective activity. Metal complexes have also emerged as an appealing avenue for drug discovery. The goal is to chelate excess metals to correct imbalances that may contribute to oxidative stress and amyloid plaque formation. While promising, challenges include ensuring specificity and safety, particularly in regard to metal-dependent enzymes required for cellular functions. Thus, further research is needed to evaluate their effectiveness (Messori et al., 2013). Notably, a ruthenium (III) complex **69** that displays potent activity against *in-vitro* Aβ aggregation has been engineered. In the pursuit of Aβ aggregation inhibition, various compounds have shown promise. This includes derivatives of bioflavonoids **71**, guanidinyl tryptophan **72**, and curcumin **73**, and all of which possess the potential to hinder the formation of Aβ aggregates. Moreover, certain compounds such as phenols and lipoic acid exhibit dual characteristics as inhibitors of Aβ aggregation and as antioxidant agents.

Glutaminyl cyclase (GC), histone deacetylase (HDAC), and beta-site amyloid precursor protein cleaving enzyme (BACE-1) are involved in the progression of Alzheimer’s disease.

One particular lesson for medicinal chemists stems from the BACE-1 inhibitor verubecestat (MK-8931), which failed to demonstrate efficacy in Phase III clinical trials and also produced adverse events such as psychiatric effects (Egan et al., 2018). On one hand the conclusions from verubecestat suggest that BACE-1 inhibition may not be able to reverse AD. Yet verubecestat indeed reduced Aβ in the cerebrospinal fluid by over 90% in both rodents and non-human primates (Kennedy et al., 2016; Scott et al., 2016), which clearly demonstrates its mechanism of action. Therefore, the physiological function of regular Aβ in brain health may be more nuanced than expected. As previously suggested when moving forward in the clinical trial setting, it may be important to obtain baseline Aβ measurements for each patient before initiation of treatments such as BACE-1 inhibitors (Doggrell, 2019). Sudden cessation of phase III clinical trials of verubecestat in 2018 was there as it did not show clinical efficacy. Additionally, it had adverse effect on hepatic system. Following lessons can be learnt from the clinical failure of verubecestat: 1) research should focus on delineating the causative mechanism of Alzheimer’s disease. 2) as we do not know exact mechanism or aetiology of Alzheimer’s disease, being medicinal chemists, we should keep ourselves abreast with the recent scientific outcomes particularly from biological area. This would help in the drug design and development of anti-AD drugs. 3) Also, it would be great to propel lead molecules which have lesser side effects particularly related to hepatotoxicity (Doggrell, 2019).

Toward further development of such inhibitors, modifications of the compound **77**, introducing a 4-methyl substitution with *N*-cyclohexyl-*N*-(1-methyl-1*H*-pyrazol-4-ylmethyl)butanamide have demonstrated robust inhibition of the BACE-1. Similar inhibitory effects on the enzyme have been noted with compounds derived from quinolone featuring a 3-carboxamide substitution **74** and derivatives of 4-aminopyridine **75** and 4,6-diaminopyridine. Another class of compounds, namely, the 1,2,4-triazine derivative **76** containing aryl phenoxy methyl-1,2,3-triazole, have also inhibited BACE-1. Notably, compounds containing chloro- or nitro-substituents at the para position of the phenyl ring, such as in compound **76**, have shown promise in inhibiting BACE-1. Glutaminyl cyclase (GC) inhibition is an approach to diminish the generation of toxic pyroforms of Aβ in the brains of Alzheimer’s patients. To this end, a series of compounds (e.g., *N*-substituted thiourea, urea, and α-substituted amide derivatives) have been synthesized and evaluated for their potential to inhibit glutaminyl cyclase. Compounds **78** and **79** have demonstrated effectiveness in this context. Additionally, inhibitors of HDAC derived from cashew nutshell liquid and its derivatives have been investigated. Strikingly compound **80** has displayed potent inhibitory activity against both HDAC1 and HDAC6 isoforms. Histone deacetylase inhibitors such as vorinostat (SAHA), FK-228 (romidepsin) and panobinostat are already available in the market as anticancer agents. To date, an array of HDAC inhibitors have been synthesized mainly to investigate its anticancer properties. Nonetheless, proper attention should be given to its potential non-cancer therapeutic benefits including its promise in the AD treatment. However, inhibition is nuanced by the complexity of existence of 12 HDAC isoforms. Therefore, a prudent opportunity manifests in development of isoform-selective HDAC inhibitors, particularly in the context of Alzheimer’s disease.

As AD is a progressive disease, these dual-target inhibitors may be a more advantageous therapeutic strategy. Various derivatives have been developed taking tacrine as a reference drug and designing homo and heterodimers having diselenides and disulfides. These disulfides containing dimers **82** showed better activity and inhibited both AChE and amyloid aggregates. Using tacrine structural features and adding resveratrol moiety has enabled the molecules to be active, but they exhibit less activity than disulfide dimers. Compound **86**, having coumarin with dithiocarbamate, emerged as a potent dual-target inhibitor against amyloid aggregates and cholinesterase. Taking this compound **86** as an initial point, comprehensive SAR studies should be performed, along with addressing other pharmacokinetic and pharmacodynamic aspects. Coumarin derivative **158** was evaluated as *MTDLs* for anti-Alzheimer activity and exhibited outstanding activity for both *hAChE* and *hBuChE* inhibition. Additionally, they demonstrated low cytotoxicity and inhibited the Aβ and tau protein aggregation. Kinetic and docking studies evaluated **158** as a mixed *hAChE* inhibitor.

Alkyl benzylamine scaffolds have been designed by developing hybrids, including plant-derived compounds. Genistein hybrids **90** with carbon spacer-linked alkyl benzylamine were active against Aβ and cholinesterase. Also, chromone-2-carboxamido alkyl benzylamine **92** and pterostilbene-O-acetamidoalkyl benzylamine **99** displayed good cholinesterase inhibitory activity, and pterostilbene derivatives showed more selectivity towards AChE. Further, the pterostilbene moiety was replaced by scutellarein **100**, resulting in analogues with increased potency. Flavonoid hybrids with aminoalkyl-substituted derivatives also emerged as promising candidates for AD treatment. *N*-benzyl piperidine molecules showed higher activity against BuChE than AChE with amyloid aggregation inhibition.

Triazine hybrids with triazolo pyrimidine and cyanopyridine moieties were active dual inhibitors of AChE and amyloid aggregates. Further substitutions have been introduced for the molecule with triazole-pyrimidine-containing hybrids **117**, resulting in di-substitution, increasing the potency over mono-substitution derivatives. Triazole containing coumarin–eugenol hybrid **157** emerged as a selective AChE inhibitor over BuChE. Additionally, these hybrids displayed inhibition of Aβ aggregates with protective properties against radicals. Simulation studies revealed advantageous interaction with AChE and the Aβ monomer intended to suppress them. Several dual inhibitors were active against AChE but displayed poor amyloid aggregation inhibition; these include pyrazole phthalazine **113** and pyridine derivative **116** with carbamic and amidic functional groups. Deoxyvascinone has also been studied for dual inhibitory activity through the introduction of diverse aminoacetamide groups having amyloid aggregation inhibition and poor *h*AChE inhibition. In contrast, benzofuran scaffold containing hybrids with carboxamide-*N*-benzyl pyridinium halide **106** were active against BuChE and inhibited amyloid aggregates.

Furthermore, several derivatives have been discovered to possess diverse activities within a single molecule, giving rise to what are known as multitarget inhibitors. A number of attempts have been made to design hybrid molecules by combining two distinct molecular features with differing activities, resulting in hybrid compounds that simultaneously target multiple aspects of the disease.

For instance, a hybrid derivative **130** has been formulated by combining CR-6 as an antioxidant with 6-chlorotacrine, linked by a carbon chain. This compound exhibited various activities, including *in-vitro* inhibition of AChE, BuChE, BACE-1 activities, and Aβ, tau aggregation. Another approach involved combining a GSK-3β inhibitor and tacrine, an *AChE* inhibitor, using a carbon linker, resulting in a compound with multi-targeted activity. Similarly, a hybrid molecule has been generated by combining rhein, huprine, and a carbon chain spacer. The hybrid molecule 149 has been created by connecting hydroxamic acid with phthalimide via a carbon linker and a thioamide functional group, resulting in first-class GSK-3β/HDAC dual inhibitors with potential as disease-modifying anti-Alzheimer’s agents. Additionally, carbazole-stilbene hybrids have been developed as multi-target anti-Alzheimer’s agents.

In addition, the natural product research has identified several naturally occurring compounds with anti-Alzheimer’s activity. Flavonoids and phenolic compounds, such as triptolide, quercetin, apigenin, bavachalcone, graveolinine derivatives, and the ethanolic extract of *Artemisia nilagirica*, exhibit potent activity against multiple targets implicated in Alzheimer’s disease.

Moreover, the chalcone derivatives **138** have revealed that those with R-substituted amino groups, particularly the incorporation of tetramethylpyrazine, showed promising activity. Quinazolinone-based derivatives **156** were developed as novel, multifunctional anti-AD drugs that exhibit both cholinesterase inhibitory and anti-inflammatory properties. Additionally, **156** displayed the usual binding pattern in the *AChE* active site with tetrahydroquinazolinone moiety. Compound **138** exhibited enhanced neuroprotection due to its trimethoxyquinazoline amino moiety substitution compared to pyrazinyl amino substitution. The strong anticholinesterase activity of compound **139** can be attributed to the pyridin-3-ylpropanone moiety in the chalcone backbone. Novel multi-functional agents aimed at treating AD have been developed, encompassing a range of heterocyclic derivatives such as, aurone Mannich derivatives **132** and *N*-alkyl piperidine carbamates **135**, piperazine derivatives **136**, bivalent β-carbolines **140**, pyrimidinylthiourea **146**. These compounds offer a promising path toward development of prospective therapeutic interventions for the multifaceted AD.

While outside the scope of our chemical review, there is also promise for antibody-based therapeutics. Notably, both Lecanemab and Donanemab are monoclonal antibodies that recognize Aβ aggregates to promote their clearance. Lecanemab demonstrated efficacy in Phase III trials by reducing cognitive decline and AD biomarkers (van Dyck et al., 2023) Donanemab is selective for the Pyroglutamate-modified version of Aβ aggregates, which has demonstrated efficacy in a recent stage III trial (Salloway et al., 2025). Ongoing trials will continue to investigate its potential and long-term safety profile.

## 6 Conclusion

The path leading to a cure for Alzheimer’s disease has a foundation composed of multiple distinct molecular targets. Here, we broadly examined the chemical space required for potential therapeutic solutions, which included the frequently explored cholinesterase inhibitors, amyloid β aggregate inhibitors, and BACE-1 inhibitors. In particular, we point out the emerging drug development potential regarding actors at glutaminyl cyclase and histone deacetylase. We are hopeful this perspective may expedite the successful quest for AD treatment. It should also be appreciated that additional targets for AD are still emerging. Indeed, recent articles report the beneficial effects of 3-aryl isocoumarin derivatives in neurodegenerative disease via activating the neurotrophin receptor, TrkB, and attenuating the inflammation by inhibiting 5-Lipoxygenase and Prostaglandin E2 production (Ramanan et al., 2016; Sudarshan et al., 2019). Hence, this could be beneficial in mitigating neuroinflammation and oxidative stress. Therefore, isocoumarin derivatives could be included in future lines of study. Although the focus of this article pertains to chemical interventions, it should be noted that gene editing, such as CRISPR/Cas9, may play a role in targeted AD treatment (Lu et al., 2021). This is potentially foreseeable in regard to genes associated with familial AD, such as APP, PSEN1, and PSEN2 mutations (Lanoiselee et al., 2017). While most treatment strategies outlined in this manuscript aim to combat and slow AD progression, unresolved challenges also include avenues that serve to enhance cognitive function. While mitigating the devastation of dementia is paramount, it may be equally important to promote learning and memory in the aging brain. This task may likely require uncovering additional biological targets as well as deeper insight into synaptic mechanisms of memory storage and retrieval. A final collective challenge for all therapeutic options discussed is uncovering the long-term safety profile for potential remedies, which underscores the need to relentlessly pursue both basic science and clinical research in AD.

## References

[B1] Abd El-Mageed M. M. A. Fattah Ezzat M. A. Moussa S. A. Abdel-Aziz H. A. Elmasry G. F. (2025). Rational design, synthesis and computational studies of multi-targeted anti-Alzheimer’s agents integrating coumarin scaffold. Bioorg Chem. 154, 108024. 10.1016/j.bioorg.2024.108024 39642754

[B2] Abd-Elrahman K. S. Sarasija S. Ferguson S. S. G. (2021). The role of neuroglial metabotropic glutamate receptors in alzheimer’s disease. Curr. Neuropharmacol. 21, 273–283. 10.2174/1570159x19666210916102638 34530715 PMC10190143

[B3] Abedinifar F. Farnia S. M. F. Mahdavi M. Nadri H. Moradi A. Ghasemi J. B. (2018). Synthesis and cholinesterase inhibitory activity of new 2-benzofuran carboxamide-benzylpyridinum salts. Bioorg Chem. 80, 180–188. 10.1016/j.bioorg.2018.06.006 29929079

[B4] Ahmad Ganai S. Ramadoss M. Mahadevan V. (2016). Histone Deacetylase (HDAC) Inhibitors-emerging roles in neuronal memory, learning, synaptic plasticity, and neural regeneration. Curr. Neuropharmacol. 14, 55–71. 10.2174/1570159X13666151021111609 26487502 PMC4787286

[B5] Altamirano-Espino J. A. Sánchez-Labastida L. A. Martínez-Archundia M. Andrade-Jorge E. Trujillo-Ferrara J. G. (2020). Acetylcholinesterase inhibition (potential anti-alzheimer effects) by aminobenzoic acid derivatives: synthesis, *in vitro* and *in silico* evaluation. ChemistrySelect 5, 14177–14182. 10.1002/slct.202003471

[B6] Alzheimer’s and Dementia (2024). Alzheimer’s association. Available online at: https://www.alz.org/alzheimer_s_dementia (Accessed on January 21, 2024).

[B7] Anand P. Singh B. (2013). A review on cholinesterase inhibitors for Alzheimer’s disease. Arch. Pharm. Res. 36, 375–399. 10.1007/s12272-013-0036-3 23435942

[B8] Armstrong R. A. (2019). Risk factors for Alzheimer’s disease. Folia Neuropathol. 57, 87–105. 10.5114/fn.2019.85929 31556570

[B9] Avgerinos K. I. Ferrucci L. Kapogiannis D. (2021). Effects of monoclonal antibodies against amyloid-β on clinical and biomarker outcomes and adverse event risks: a systematic review and meta-analysis of phase III RCTs in Alzheimer’s disease. Ageing Res. Rev. 68, 101339. 10.1016/j.arr.2021.101339 33831607 PMC8161699

[B10] Badran M. M. Hakeem M. A. Abuel-Maaty S. M. El-Malah A. Abdel Salam R. M. (2013). Design and synthesis of thienopyridines as novel templates for acetylcholinesterase inhibitors. Med. Chem. Res. 22, 4087–4095. 10.1007/s00044-012-0403-5

[B11] Bagaria J. Bagyinszky E. An S. S. A. (2022). Genetics, functions, and clinical impact of presenilin-1 (PSEN1) gene. Int. J. Mol. Sci. 23, 10970. 10.3390/ijms231810970 36142879 PMC9504248

[B12] Barajas-Carrillo V. W. Estolano-Cobián A. Díaz-Rubio L. Ayllón-Gutiérrez R. R. Salazar-Aranda R. Díaz-Molina R. (2021). Antioxidant and acetylcholinesterase inhibition activity of aliphatic and aromatic edaravone derivatives. Med. Chem. Res. 30, 610–623. 10.1007/s00044-020-02667-5

[B13] Bayer T. A. (2022). Pyroglutamate Aβ cascade as drug target in Alzheimer’s disease. Mol. Psychiatry 27, 1880–1885. 10.1038/s41380-021-01409-2 34880449 PMC9126800

[B14] Bazzari F. H. Bazzari A. H. (2022). BACE1 inhibitors for alzheimer’s disease: the past, present and any future? Molecules 27, 8823. 10.3390/molecules27248823 36557955 PMC9785888

[B15] Benhamú B. Martín-Fontecha M. Vázquez-Villa H. Pardo L. López-Rodríguez M. L. (2014). Serotonin 5-HT6receptor antagonists for the treatment of cognitive deficiency in Alzheimer’s disease. J. Med. Chem. 57, 7160–7181. 10.1021/jm5003952 24850589

[B16] Bhansali P. Hanigan C. L. Casero R. A. Jr. Tillekeratne L. M. V. (2011). Largazole and analogues with modified metal-binding motifs targeting histone deacetylases: synthesis and biological evaluation. J. Med. Chem. 54, 7453–7463. 10.1021/jm200432a 21936551 PMC3208063

[B17] Bhansali P. Hanigan C. L. Perera L. Casero R. A. Tillekeratne L. M. V. (2014). Synthesis and biological evaluation of largazole analogues with modified surface recognition cap groups. Eur. J. Med. Chem. 86, 528–541. 10.1016/j.ejmech.2014.09.009 25203782 PMC4188730

[B18] Bon L. Banas A. Dias I. Melo-Marques I. Cardoso S. Chaves S. (2024). New multitarget rivastigmine–indole hybrids as potential drug candidates for alzheimer’s disease. Pharmaceutics 16, 281. 10.3390/pharmaceutics16020281 38399339 PMC10892719

[B19] Borodovitsyna O. Flamini M. Chandler D. (2017). Noradrenergic modulation of cognition in health and disease. Neural Plast. 11, 6031478. 10.1155/2017/6031478 28596922 PMC5450174

[B20] Breijyeh Z. Karaman R. (2020). Comprehensive review on alzheimer’s disease: causes and treatment. Molecules 25, 5789. 10.3390/MOLECULES25245789 33302541 PMC7764106

[B21] Brown A. J. H. Bradley S. J. Marshall F. H. Brown G. A. Bennett K. A. Brown J. (2021). From structure to clinic: design of a muscarinic M1 receptor agonist with potential to treatment of Alzheimer’s disease. Cell 184, 5886–5901.e22. 10.1016/j.cell.2021.11.001 34822784 PMC7616177

[B22] Bsamen M. H. R. Bluhm A. Piechotta A. Linnert M. Rahfeld J. U. Demuth H. U. (2018). Immunohistochemical evidence from APP-transgenic mice for glutaminyl cyclase as drug target to diminish pE-abeta formation. Molecules 23. 10.3390/molecules23040924 PMC6017857 29673150

[B23] Camargo L. C. Schöneck M. Sangarapillai N. Honold D. Shah N. J. Langen K. J. (2021). Peaβ triggers cognitive decline and amyloid burden in a novel mouse model of alzheimer’s disease. Int. J. Mol. Sci. 22, 7062. 10.3390/ijms22137062 34209113 PMC8267711

[B24] Camps P. Formosa X. Galdeano C. Muñoz-Torrero D. Ramírez L. Gómez E. (2009). Pyrano[3,2-c]quinoline - 6-chlorotacrine hybrids as a novel family of acetylcholinesterase-and β-amyloid-directed anti-Alzheimer compounds. J. Med. Chem. 52, 5365–5379. 10.1021/jm900859q 19663388

[B25] Cen J. Guo H. Hong C. Lv J. Yang Y. Wang T. (2017). Development of tacrine-bifendate conjugates with improved cholinesterase inhibitory and pro-cognitive efficacy and reduced hepatotoxicity. Eur. J. Med. Chem. 144, 128–136. 10.1016/j.ejmech.2017.12.005 29268129

[B26] Çevik U. A. Osmaniye D. Sağlik B. N. Çavuşoğlu B. K. Levent S. Karaduman A. B. (2020). Multifunctional quinoxaline-hydrazone derivatives with acetylcholinesterase and monoamine oxidases inhibitory activities as potential agents against Alzheimer’s disease. Med. Chem. Res. 29, 1000–1011. 10.1007/s00044-020-02541-4

[B27] Chand K. Alsoghier H. M. Chaves S. Santos M. A. (2016). Tacrine-(hydroxybenzoyl-pyridone) hybrids as potential multifunctional anti-Alzheimer’s agents: AChE inhibition, antioxidant activity and metal chelating capacity. J. Inorg. Biochem. 163, 266–277. 10.1016/j.jinorgbio.2016.05.005 27235273

[B28] Chen J. Long Z. Li Y. Luo M. Luo S. He G. (2019). Alteration of the Wnt/GSK3β/β-catenin signalling pathway by rapamycin ameliorates pathology in an Alzheimer’s disease model. Int. J. Mol. Med. 44, 313–323. 10.3892/ijmm.2019.4198 31115485

[B29] Cho Y. Cavalli V. (2014). HDAC signaling in neuronal development and axon regeneration. Curr. Opin. Neurobiol. 27, 118–126. 10.1016/j.conb.2014.03.008 24727244 PMC4122610

[B30] Choubey P. Tripathi A. Tripathi M. Seth A. Shrivastava S. (2021). Design, synthesis, and evaluation of N-benzylpyrrolidine and 1,3,4-oxadiazole as multitargeted hybrids for the treatment of alzheimer’s disease. Bioorg Chem. 111, 104922. 10.1016/j.bioorg.2021.104922 33945941

[B31] Chung B. Y. T. Bignell W. Jacklin D. L. Winters B. D. Bailey C. D. C. (2016). Postsynaptic nicotinic acetylcholine receptors facilitate excitation of developing CA1 pyramidal neurons. J. Neurophysiol. 116, 2043–2055. 10.1152/jn.00370.2016 27489367 PMC5102319

[B32] Coimbra J. Moreira P. Santos A. Salvador J. (2023). Therapeutic potential of glutaminyl cyclases: current status and emerging trends. Drug Discov. Today 28, 103644. 10.1016/j.drudis.2023.103644 37244566

[B33] Cooper D. M. F. (2003). Regulation and organization of adenylyl cyclases and cAMP. Biochem. J. 375, 517–529. 10.1042/bj20031061 12940771 PMC1223734

[B34] Cuccioli V. Bueno C. Belvindrah R. Lledo P. M. Martinez S. (2015). Attractive action of FGF-signaling contributes to the postnatal developing hippocampus. Hippocampus 25, 486–499. 10.1002/hipo.22386 25348908

[B35] Cynis H. Scheel E. Saido T. C. Schilling S. Demuth H. U. (2008). Amyloidogenic processing of amyloid precursor protein: evidence of a pivotal role of glutaminyl cyclase in generation of pyroglutamate-modified amyloid-beta. Biochemistry 47, 7405–7413. 10.1021/bi800250p 18570439

[B36] Czarnota-Łydka K. Kucwaj-Brysz K. Pyka P. Haberek W. Podlewska S. Handzlik J. (2022). Multitargeting the action of 5-HT6 serotonin receptor ligands by additional modulation of kinases in the search for a new therapy for alzheimer’s disease: can it work from a molecular point of view? Int. J. Mol. Sci. 23, 8768. 10.3390/ijms23158768 35955902 PMC9368844

[B37] Das N. Raymick J. Sarkar S. (2021). Role of metals in Alzheimer’s disease. Metab. Brain Dis. 36, 1627–1639. 10.1007/s11011-021-00765-w 34313926

[B38] Davis S. M. Eckroat T. J. (2021). Isatin-linked 4,4-dimethyl-5-methylene-4,5-dihydrothiazole-2-thiols for inhibition of acetylcholinesterase. Med. Chem. Res. 30, 2289–2300. 10.1007/s00044-021-02800-y

[B39] De Simone A. La Pietra V. Betari N. Petragnani N. Conte M. Daniele S. (2019). Discovery of the first-in-class GSK-3β/HDAC dual inhibitor as disease-modifying agent to combat alzheimer’s disease. ACS Med. Chem. Lett. 10, 469–474. 10.1021/acsmedchemlett.8b00507 30996781 PMC6466523

[B40] Del Arco A. Mora F. (2005). Glutamate-dopamine *in vivo* interaction in the prefrontal cortex modulates the release of dopamine and acetylcholine in the nucleus accumbens of the awake rat. J. Neural Transm. 112, 97–109. 10.1007/s00702-004-0172-5 15599608

[B41] Dennis S. H. Pasqui F. Colvin E. M. Sanger H. Mogg A. J. Felder C. C. (2016). Activation of muscarinic M1 acetylcholine receptors induces long-term potentiation in the Hippocampus. Cereb. Cortex 26, 414–426. 10.1093/cercor/bhv227 26472558 PMC4677984

[B42] Derabli C. Boualia I. Abdelwahab A. B. Boulcina R. Bensouici C. Kirsch G. (2018). A cascade synthesis, *in vitro* cholinesterases inhibitory activity and docking studies of novel Tacrine-pyranopyrazole derivatives. Bioorg Med. Chem. Lett. 28, 2481–2484. 10.1016/j.bmcl.2018.05.063 29887354

[B43] Descalzi G. Ikegami D. Ushijima T. Nestler E. J. Zachariou V. Narita M. (2015). Epigenetic mechanisms of chronic pain. Trends Neurosci. 38, 237–246. 10.1016/j.tins.2015.02.001 25765319 PMC4459752

[B44] Dickinson B. A. Jo J. Seok H. Son G. H. Whitcomb D. J. Davies C. H. (2009). A novel mechanism of hippocampal LTD involving muscarinic receptor-triggered interactions between AMPARs, GRIP and liprin-. Mol. Brain 2, 1–12. 10.1186/1756-6606-2-18 19534762 PMC2701934

[B45] Doggrell S. A. (2019). Lessons that can be learnt from the failure of verubecestat in Alzheimer’s disease. Expert Opin. Pharmacother. 20, 2095–2099. 10.1080/14656566.2019.1654998 31423903

[B46] Donovan E. Avila C. Klausner S. Parikh V. Fenollar-Ferrer C. Blakely R. D. (2022). Disrupted choline clearance and sustained acetylcholine release *in vivo* by a common choline transporter coding variant associated with poor attentional control in humans. J. Neurosci. 42, 3426–3444. 10.1523/JNEUROSCI.1334-21.2022 35232764 PMC9034784

[B47] Ebrahimi S. E. S. Ghadirian P. Emtiazi H. Yahya-Meymandi A. Saeedi M. Mahdavi M. (2016). Hetero-annulated coumarins as new AChE/BuChE inhibitors: synthesis and biological evaluation. Med. Chem. Res. 25, 1831–1841. 10.1007/s00044-016-1626-7

[B48] Egan M. Kost J. Tariot P. Aisen P. Cummings J. Vellas B. (2018). Randomized trial of verubecestat for mild-to-moderate alzheimer’s disease. N. Engl. J. Med. 378, 1691–1703. 10.1056/NEJMoa1706441 29719179 PMC6776074

[B49] Eghtedari M. Sarrafi Y. Nadri H. Mahdavi M. Moradi A. Homayouni Moghadam F. (2017). New tacrine-derived AChE/BuChE inhibitors: synthesis and biological evaluation of 5-amino-2-phenyl-4H-pyrano[2,3-b]quinoline-3-carboxylates. Eur. J. Med. Chem. 128, 237–246. 10.1016/j.ejmech.2017.01.042 28189905

[B50] Elliott C. Rojo A. I. Ribe E. Broadstock M. Xia W. Morin P. (2018). A role for APP in Wnt signalling links synapse loss with β-amyloid production. Transl. Psychiatry 8, 179. 10.1038/s41398-018-0231-6 30232325 PMC6145937

[B51] Erdogan M. Kilic B. Sagkan R. I. Aksakal F. Ercetin T. Gulcan H. O. (2021). Design, synthesis and biological evaluation of new benzoxazolone/benzothiazolone derivatives as multi-target agents against Alzheimer’s disease. Eur. J. Med. Chem. 212, 113124. 10.1016/j.ejmech.2020.113124 33395623

[B52] Espargaró A. Ginex T. Vadell M. del M. Busquets M. A. Estelrich J. Muñoz-Torrero D. (2017). Combined *in vitro* cell-based/*in silico* screening of naturally occurring flavonoids and phenolic compounds as potential anti-alzheimer drugs. J. Nat. Prod. 80, 278–289. 10.1021/acs.jnatprod.6b00643 28128562

[B53] Estrada M. Herrera-Arozamena C. Pérez C. Viña D. Romero A. Morales-García J. A. (2016). New cinnamic - N-benzylpiperidine and cinnamic - N,N-dibenzyl(N-methyl)amine hybrids as Alzheimer-directed multitarget drugs with antioxidant, cholinergic, neuroprotective and neurogenic properties. Eur. J. Med. Chem. 121, 376–386. 10.1016/j.ejmech.2016.05.055 27267007

[B54] Exley R. Cragg S. J. (2008). Presynaptic nicotinic receptors: a dynamic and diverse cholinergic filter of striatal dopamine neurotransmission. Br. J. Pharm. 153, S283–S297. 10.1038/sj.bjp.0707510 18037926 PMC2268048

[B55] Falkenberg K. J. Johnstone R. W. (2014). Histone deacetylases and their inhibitors in cancer, neurological diseases and immune disorders. Nat. Rev. Drug Discov. 13, 673–691. 10.1038/nrd4360 25131830

[B56] Fang L. Appenroth D. Decker M. Kiehntopf M. Roegler C. Deufel T. (2008). Synthesis and biological evaluation of NO-donor-tacrine hybrids as hepatoprotective anti-Alzheimer drug candidates. J. Med. Chem. 51, 713–716. 10.1021/jm701491k 18232655

[B57] Faraji L. Nadri H. Moradi A. Bukhari S. N. A. Pakseresht B. Moghadam F. H. (2019). Aminoalkyl-substituted flavonoids: synthesis, cholinesterase inhibition, β-amyloid aggregation, and neuroprotective study. Med. Chem. Res. 28, 974–983. 10.1007/s00044-019-02350-4

[B58] Fernández De Sevilla D. Núñez A. Borde M. Malinow R. Buño W. (2008). Cholinergic-mediated IP3-receptor activation induces long-lasting synaptic enhancement in CA1 pyramidal neurons. J. Neurosci. 28, 1469–1478. 10.1523/JNEUROSCI.2723-07.2008 18256268 PMC6671582

[B59] Fischer W. H. Spiess J. (1987). Identification of a mammalian glutaminyl cyclase converting glutaminyl into pyroglutamyl peptides. Proc. Natl. Acad. Sci. U. S. A. 84, 3628–3632. 10.1073/pnas.84.11.3628 3473473 PMC304928

[B60] Frisoni G. B. Altomare D. Thal D. R. Ribaldi F. van der Kant R. Ossenkoppele R. (2022). The probabilistic model of Alzheimer disease: the amyloid hypothesis revised. Nat. Rev. Neurosci. 23, 53–66. 10.1038/s41583-021-00533-w 34815562 PMC8840505

[B61] Gabr M. T. Abdel-Raziq M. S. (2018). Design and synthesis of donepezil analogues as dual AChE and BACE-1 inhibitors. Bioorg Chem. 80, 245–252. 10.1016/j.bioorg.2018.06.031 29966870

[B62] Gannon M. Che P. Chen Y. Jiao K. Roberson E. D. Wang Q. (2015). Noradrenergic dysfunction in Alzheimer’s disease. Front. Neurosci. 9, 220. 10.3389/fnins.2015.00220 26136654 PMC4469831

[B63] Ge S. Dani J. A. (2005). Nicotinic acetylcholine receptors at glutamate synapses facilitate long-term depression or potentiation. J. Neurosci. 25, 6084–6091. 10.1523/JNEUROSCI.0542-05.2005 15987938 PMC6725070

[B64] Ghobadian R. Nadri H. Moradi A. Bukhari S. N. A. Mahdavi M. Asadi M. (2018). Design, synthesis, and biological evaluation of selective and potent Carbazole-based butyrylcholinesterase inhibitors. Bioorg Med. Chem. 26, 4952–4962. 10.1016/j.bmc.2018.08.035 30190181

[B65] Gök N. Akıncıoğlu A. Erümit Binici E. Akıncıoğlu H. Kılınç N. Göksu S. (2021). Synthesis of novel sulfonamides with anti-Alzheimer and antioxidant capacities. Arch. Pharm. Weinh. 354, 2000496. 10.1002/ardp.202000496 33749025

[B66] Guan J. S. Haggarty S. J. Giacometti E. Dannenberg J. H. Joseph N. Gao J. (2009). HDAC2 negatively regulates memory formation and synaptic plasticity. Nature 459, 55–60. 10.1038/nature07925 19424149 PMC3498958

[B67] Gunn A. P. Wong B. X. McLean C. Fowler C. Barnard P. J. Duce J. A. (2021). Increased glutaminyl cyclase activity in brains of Alzheimer’s disease individuals. J. Neurochem. 156, 979–987. 10.1111/jnc.15114 32614980

[B68] Gupta M. Ojha M. Yadav D. Pant S. Yadav R. (2020). Novel benzylated (Pyrrolidin-2-one)/(Imidazolidin-2-one) derivatives as potential anti-alzheimer’s agents: synthesis and pharmacological investigations. ACS Chem. Neurosci. 11, 2849–2860. 10.1021/acschemneuro.0c00403 32816447

[B69] Haam J. Yakel J. L. (2017). Cholinergic modulation of the hippocampal region and memory function. J. Neurochem. 142, 111–121. 10.1111/jnc.14052 28791706 PMC5645066

[B70] Halder N. Lal G. (2021). Cholinergic system and its therapeutic importance in inflammation and autoimmunity. Front. Immunol. 12. 10.3389/fimmu.2021.660342 33936095 PMC8082108

[B71] Hampel H. Mesulam M. M. Cuello A. C. Farlow M. R. Giacobini E. Grossberg G. T. (2018). The cholinergic system in the pathophysiology and treatment of Alzheimer’s disease. Brain 141, 1917–1933. 10.1093/brain/awy132 29850777 PMC6022632

[B72] Hamze A. (2020). How do we improve histone deacetylase inhibitor drug discovery? Expert Opin. Drug Discov. 15, 527–529. 10.1080/17460441.2020.1736032 32116055

[B73] Haque M. E. Kim I. S. Jakaria M. Akther M. Choi D. K. (2018). Importance of GPCR-mediated microglial activation in alzheimer’s disease. Front. Cell Neurosci. 12, 258. 10.3389/fncel.2018.00258 30186116 PMC6110855

[B74] Hasselmo M. E. (2006). The role of acetylcholine in learning and memory. Curr. Opin. Neurobiol. 16, 710–715. 10.1016/j.conb.2006.09.002 17011181 PMC2659740

[B75] He F. Ran Y. Li X. Wang D. Zhang Q. Lv J. (2020). Design, synthesis and biological evaluation of dual-function inhibitors targeting NMDAR and HDAC for Alzheimer’s disease. Bioorg Chem. 103, 104109. 10.1016/j.bioorg.2020.104109 32768741

[B76] He Y. Xiao G. Yu G. Song Q. Zhang H. Liu Z. (2021). 2-(3-Hydroxybenzyl)benzo[d]isothiazol-3(2H)-one Mannich base derivatives as potential multifunctional anti-Alzheimer’s agents. Med. Chem. Res. 30, 1249–1264. 10.1007/s00044-021-02725-6

[B77] Hedrick T. Waters J. (2015). Acetylcholine excites neocortical pyramidal neurons via nicotinic receptors. J. Neurophysiol. 113, 2195–2209. 10.1152/jn.00716.2014 25589590 PMC4416587

[B78] Hellström-Lindahl E. Viitanen M. Marutle A. (2009). Comparison of Abeta levels in the brain of familial and sporadic Alzheimer's disease. Neurochem. Int. 55, 243–252. 10.1016/j.neuint.2009.03.007 19524115 PMC2919843

[B79] Hepnarova V. Korabecny J. Matouskova L. Jost P. Muckova L. Hrabinova M. (2018). The concept of hybrid molecules of tacrine and benzyl quinolone carboxylic acid (BQCA) as multifunctional agents for Alzheimer’s disease. Eur. J. Med. Chem. 150, 292–306. 10.1016/j.ejmech.2018.02.083 29533874

[B80] Hira S. Saleem U. Anwar F. Raza Z. Rehman A. U. Ahmad B. (2020). *In silico* study and pharmacological evaluation of Eplerinone as an anti-alzheimer’s drug in STZ-induced alzheimer’s disease model. ACS Omega 5, 13973–13983. 10.1021/acsomega.0c01381 32566864 PMC7301577

[B81] Hiremathad A. Chand K. Tolayan L. Rajeshwari Keri R. S. Esteves A. R. (2018a). Hydroxypyridinone-benzofuran hybrids with potential protective roles for Alzheimer´s disease therapy. J. Inorg. Biochem. 179, 82–96. 10.1016/j.jinorgbio.2017.11.015 29182921

[B82] Hiremathad A. Keri R. S. Esteves A. R. Cardoso S. M. Chaves S. Santos M. A. (2018b). Novel Tacrine-Hydroxyphenylbenzimidazole hybrids as potential multitarget drug candidates for Alzheimer’s disease. Eur. J. Med. Chem. 148, 255–267. 10.1016/j.ejmech.2018.02.023 29466775

[B83] Hoang V. H. Tran P. T. Cui M. Ngo V. T. H. Ann J. Park J. (2017). Discovery of potent human glutaminyl cyclase inhibitors as anti-alzheimer’s agents based on rational design. J. Med. Chem. 60, 2573–2590. 10.1021/acs.jmedchem.7b00098 28234463

[B84] Hoang V. H. Ngo V. T. H. Cui M. Manh N. V. Tran P. T. Ann J. (2019). Discovery of conformationally restricted human glutaminyl cyclase inhibitors as potent anti-alzheimer’s agents by structure-based design. J. Med. Chem. 62, 8011–8027. 10.1021/acs.jmedchem.9b00751 31411468

[B85] Holsinger R. M. D. Goense N. Bohorquez J. Strappe P. (2013). Splice variants of the Alzheimer’s disease beta-secretase, BACE1. Neurogenetics 14, 1–9. 10.1007/s10048-012-0348-3 23142975

[B86] Hook G. Yu J. Toneff T. Kindy M. Hook V. (2014). Brain pyroglutamate amyloid-β is produced by cathepsin b and is reduced by the cysteine protease inhibitor E64d, representing a potential alzheimer’s disease therapeutic. J. Alzheimer’s Dis. 41, 129–149. 10.3233/JAD-131370 24595198 PMC4059604

[B87] Hosseini F. Mohammadi-Khanaposhtani M. Azizian H. Ramazani A. Barazandeh M. Nadri H. (2020). 4-Oxobenzo[d]1,2,3-triazin-pyridinium-phenylacetamide derivatives as new anti-Alzheimer agents: design, synthesis, *in vitro* evaluation, molecular modeling, and molecular dynamic study. Struct. Chem. 31, 999–1012. 10.1007/s11224-019-01472-0

[B88] Hu Y. H. Yang J. Zhang Y. Liu K. C. Liu T. Sun J. (2019). Synthesis and biological evaluation of 3–(4-aminophenyl)-coumarin derivatives as potential anti-Alzheimer’s disease agents. J. Enzyme Inhib. Med. Chem. 34, 1083–1092. 10.1080/14756366.2019.1615484 31117844 PMC6534212

[B89] Huang K. F. Liaw S. S. Huang W. L. Chia C. Y. Lo Y. C. Chen Y. L. (2011). Structures of human golgi-resident glutaminyl cyclase and its complexes with inhibitors reveal a large loop movement upon inhibitor binding. Biol. Chemi 286, 12439–12449. 10.1074/jbc.M110.208595 21288892 PMC3069447

[B90] Huang Y. Skwarek-Maruszewska A. Horré K. Vandewyer E. Wolfs L. Snellinx A. (2015). Loss of GPR3 reduces the amyloid plaque burden and improves memory in Alzheimer’s disease mouse models. Sci. Transl. Med. 7, 309ra164. 10.1126/scitranslmed.aab3492 26468326

[B91] Hunt C. E. Turner A. J. (2009). Cell biology, regulation and inhibition of β-secretase (BACE-1). FEBS J. 276, 1845–1859. 10.1111/j.1742-4658.2009.06929.x 19292866

[B92] Hur J. Y. (2022). γ-Secretase in Alzheimer’s disease. Exp. Mol. Med. 54, 433–446. 10.1038/s12276-022-00754-8 35396575 PMC9076685

[B93] Jagtap A. D. Kondekar N. B. Hung P. Y. Hsieh C. E. Yang C. R. Chen G. S. (2020). 4-Substituted 2-amino-3,4-dihydroquinazolines with a 3-hairpin turn side chain as novel inhibitors of BACE-1. Bioorg Chem. 95, 103135. 10.1016/j.bioorg.2019.103135 31923631

[B94] Jalili-Baleh L. Nadri H. Moradi A. Bukhari S. N. A. Shakibaie M. Jafari M. (2017). New racemic annulated pyrazolo[1,2-b]phthalazines as tacrine-like AChE inhibitors with potential use in Alzheimer’s disease. Eur. J. Med. Chem. 139, 280–289. 10.1016/j.ejmech.2017.07.072 28803044

[B95] Jameel E. Meena P. Maqbool M. Kumar J. Ahmed W. Mumtazuddin S. (2017). Rational design, synthesis and biological screening of triazine-triazolopyrimidine hybrids as multitarget anti-Alzheimer agents. Eur. J. Med. Chem. 136, 36–51. 10.1016/j.ejmech.2017.04.064 28478343

[B96] Jawhar S. Wirths O. Bayer T. A. (2011). Pyroglutamate amyloid-β (Aβ): a hatchet man in alzheimer disease. J. Biol. Chem. 286, 38825–38832. 10.1074/jbc.R111.288308 21965666 PMC3234707

[B97] Jeřábek J. Uliassi E. Guidotti L. Korábečný J. Soukup O. Sepsova V. (2017). Tacrine-resveratrol fused hybrids as multi-target-directed ligands against Alzheimer’s disease. Eur. J. Med. Chem. 127, 250–262. 10.1016/j.ejmech.2016.12.048 28064079

[B98] Jiang N. Huang Q. Liu J. Liang N. Li Q. Li Q. (2018a). Design, synthesis and biological evaluation of new coumarin-dithiocarbamate hybrids as multifunctional agents for the treatment of Alzheimer’s disease. Eur. J. Med. Chem. 146, 287–298. 10.1016/j.ejmech.2018.01.055 29407958

[B99] Jiang X. Y. Chen T. K. Zhou J. T. He S. Y. Yang H. Y. Chen Y. (2018b). Dual GSK-3β/AChE inhibitors as a new strategy for multitargeting anti-alzheimer’s disease drug discovery. ACS Med. Chem. Lett. 9, 171–176. 10.1021/acsmedchemlett.7b00463 29541355 PMC5846044

[B100] Jo J. Son G. H. Winters B. L. Kim M. J. Whitcomb D. J. Dickinson B. A. (2010). Muscarinic receptors induce LTD of NMDAR EPSCs via a mechanism involving hippocalcin, AP2 and PSD-95. Nat. Neurosci. 13, 1216–1224. 10.1038/nn.2636 20852624

[B101] Joubert J. Foka G. B. Repsold B. P. Oliver D. W. Kapp E. Malan S. F. (2017). Synthesis and evaluation of 7-substituted coumarin derivatives as multimodal monoamine oxidase-B and cholinesterase inhibitors for the treatment of Alzheimer’s disease. Eur. J. Med. Chem. 125, 853–864. 10.1016/j.ejmech.2016.09.041 27744252

[B102] Jouvenceau A. Hédou G. Potier B. Kollen M. Dutar P. Mansuy I. M. (2006). Partial inhibition of PP1 alters bidirectional synaptic plasticity in the hippocampus. J. Neurosci. 24, 564–572. 10.1111/j.1460-9568.2006.04938.x 16903858

[B103] Kamal A. Shaik A. B. Reddy G. N. Kumar C. G. Joseph J. Kumar G. B. (2014). Synthesis, biologicalevaluation, and molecular modeling of (E)-2-aryl-5-styryl-1,3,4-oxadiazolederivatives as acetylcholine esterase inhibitors. Med. Chem. Res. 23, 2080–2092. 10.1007/s00044-013-0786-y

[B104] Kandalepas P. C. Vassar R. (2012). Identification and biology of β-secretase. J. Neurochem. 120, 55–61. 10.1111/j.1471-4159.2011.07512.x 22122287

[B105] Kandel E. R. (2012). The molecular biology of memory: CAMP, PKA, CRE, CREB-1, CREB-2, and CPEB. Mol. Brain 5, 14–12. 10.1186/1756-6606-5-14 22583753 PMC3514210

[B106] Kandiah N. Pai M. C. Senanarong V. Looi I. Ampil E. Park K. W. (2017). Rivastigmine: the advantages of dual inhibition of acetylcholinesterase and butyrylcholinesterase and its role in subcortical vascular dementia and Parkinson’s disease dementia. Clin. Interv. Aging 12, 697–707. 10.2147/CIA.S129145 28458525 PMC5402908

[B107] Kanhed A. M. Patel D. V. Patel N. R. Sinha A. Thakor P. S. Patel K. B. (2022). Indoloquinoxaline derivatives as promising multi-functional anti-Alzheimer agents. J. Biomol. Struct. Dyn. 40, 1–18. 10.1080/07391102.2020.1840441 33111617

[B108] Kara J. Suwanhom P. Wattanapiromsakul C. Nualnoi T. Puripattanavong J. Khongkow P. (2019). Synthesis of 2-(2-oxo-2H-chromen-4-yl)acetamides as potent acetylcholinesterase inhibitors and molecular insights into binding interactions. Arch. Pharm. Weinh. 352, e1800310. 10.1002/ardp.201800310 31125474

[B109] Kassab R. (2023). Acetylcholine-sensitive control of long-term synaptic potentiation in hippocampal CA3 neurons. Hippocampus 33, 948–969. 10.1002/hipo.23533 37016759

[B110] Kempermann G. Song H. Gage F. H. (2015). Neurogenesis in the adult Hippocampus. Cold Spring Harb. Perspect. Biol. 7, a018812. 10.1101/cshperspect.a018812 26330519 PMC4563705

[B111] Kennedy M. Stamford A. Chen X. Cox K. Cumming J. Dockendorf M. (2016). The BACE1 inhibitor verubecestat (MK-8931) reduces CNS β-amyloid in animal models and in Alzheimer's disease patients. Sci. Transl. Med. 8, 363ra150. 10.1126/scitranslmed.aad9704 27807285

[B112] Knez D. Brus B. Coquelle N. Sosič I. Šink R. Brazzolotto X. (2015). Structure-based development of nitroxoline derivatives as potential multifunctional anti-Alzheimer agents. Bioorg Med. Chem. 23, 4442–4452. 10.1016/j.bmc.2015.06.010 26116179

[B113] Koca M. Yerdelen K. O. Anil B. Kasap Z. Sevindik H. Ozyurek I. (2016). Design, synthesis and biological activity of 1H-indene-2-carboxamides as multi-targeted anti-Alzheimer agents. J. Enzyme Inhib. Med. Chem. 31, 13–23. 10.1080/14756366.2016.1186019 27226239

[B114] Košak U. Strašek N. Knez D. Jukič M. Žakelj S. Zahirović A. (2020). N-alkylpiperidine carbamates as potential anti-Alzheimer’s agents. Eur. J. Med. Chem. 197, 112282. 10.1016/j.ejmech.2020.112282 32380361

[B115] Kulshreshtha A. Piplani P. (2018). Design, synthesis and pharmacological evaluation of carboxamide and carbothioamide derivatives of 1,3,4-thiadiazole as the inhibitors of acetylcholinesterase and oxipiperazine ative stress for the management of cognitive debility. Med. Chem. Res. 27, 1800–1821. 10.1007/s00044-018-2193-x

[B116] Kumar A. Dhull D. K. Mishra P. S. (2015). Therapeutic potential of mGluR5 targeting in Alzheimer’s disease. Front. Neurosci. 9, 215. 10.3389/fnins.2015.00215 26106290 PMC4460345

[B117] Lane R. M. Potkin S. G. Enz A. (2006). Targeting acetylcholinesterase and butyrylcholinesterase in dementia. Int. J. Neuropsychopharmacol. 9, 101–124. 10.1017/S1461145705005833 16083515

[B118] Lane C. A. Hardy J. Schott J. M. (2018). Alzheimer’s disease. Eur. J. Neurol. 25, 59–70. 10.1111/ene.13439 28872215

[B119] Lanoiselee H. Nicolas G. Wallon D. Rovelet-Lecrux A. Lacour M. Rousseau S. (2017). APP, PSEN1, and PSEN2 mutations in early-onset Alzheimer disease: a genetic screening study of familial and sporadic cases. PLoS Med. 14, e1002270. 10.1371/journal.pmed.1002270 28350801 PMC5370101

[B120] Latcheva N. K. Delaney T. L. Viveiros J. M. Smith R. A. Bernard K. M. Harsin B. (2019). The CHD protein, kismet, is important for the recycling of synaptic Vesicles during endocytosis. Sci. Rep. 9, 19368. 10.1038/s41598-019-55900-6 31852969 PMC6920434

[B121] Lee Y. H. Shin M. C. Yun Y. D. Shin S. Y. Kim J. M. Seo J. M. (2015). Synthesis of aminoalkyl-substituted aurone derivatives as acetylcholinesterase inhibitors. Bioorg Med. Chem. 23, 231–240. 10.1016/j.bmc.2014.11.004 25468034

[B122] Lei P. Ayton S. Bush A. I. (2021). The essential elements of Alzheimer’s disease. J. Biol. Chem. 296, 100105. 10.1074/jbc.REV120.008207 33219130 PMC7948403

[B123] Leng J. Qin H. L. Zhu K. Jantan I. Hussain M. A. Sher M. (2016). Evaluation of multifunctional synthetic tetralone derivatives for treatment of Alzheimer’s disease. Chem. Biol. Drug Des. 88, 889–898. 10.1111/cbdd.12822 27434226

[B124] Lester D. B. Rogers T. D. Blaha C. D. (2010). Acetylcholine-dopamine interactions in the pathophysiology and treatment of CNS disorders. CNS Neurosci. Ther. 16, 137–162. 10.1111/j.1755-5949.2010.00142.x 20370804 PMC6493877

[B125] Li Y. Zhang X. X. Jiang L. J. Yuan L. Cao T. T. Li X. (2015). Inhibition of acetylcholinesterase (AChE): a potential therapeutic target to treat alzheimer’s disease. Chem. Biol. Drug Des. 86, 776–782. 10.1111/cbdd.12550 25736722

[B126] Li J. C. Zhang J. Rodrigues M. C. Ding D. J. Longo J. P. F. Azevedo R. B. (2016a). Synthesis and evaluation of novel 1,2,3-triazole-based acetylcholinesterase inhibitors with neuroprotective activity. Bioorg Med. Chem. Lett. 26, 3881–3885. 10.1016/j.bmcl.2016.07.017 27426301

[B127] Li L. Chen M. Jiang F. C. (2016b). Design, synthesis, and evaluation of 2-piperidone derivatives for the inhibition of β-amyloid aggregation and inflammation mediated neurotoxicity. Bioorg Med. Chem. 24, 1853–1865. 10.1016/j.bmc.2016.03.010 26972922

[B128] Li X. Wang H. Lu Z. Zheng X. Ni W. Zhu J. (2016c). Development of multifunctional pyrimidinylthiourea derivatives as potential anti-alzheimer agents. J. Med. Chem. 59, 8326–8344. 10.1021/acs.jmedchem.6b00636 27552582

[B129] Li Y. Qiang X. Li Y. Yang X. Luo L. Xiao G. (2016d). Pterostilbene-O-acetamidoalkylbenzylamines derivatives as novel dual inhibitors of cholinesterase with anti-β-amyloid aggregation and antioxidant properties for the treatment of Alzheimer’s disease. Bioorg Med. Chem. Lett. 26, 2035–2039. 10.1016/j.bmcl.2016.02.079 26947607

[B130] Li Y. Qiang X. Luo L. Yang X. Xiao G. Liu Q. (2017). Aurone Mannich base derivatives as promising multifunctional agents with acetylcholinesterase inhibition, anti-β-amyloid aggragation and neuroprotective properties for the treatment of Alzheimer’s disease. Eur. J. Med. Chem. 126, 762–775. 10.1016/j.ejmech.2016.12.009 27951485

[B131] Liu P. Niu Y. Wang C. Sun Q. Zhai Y. Yu J. (2014). 4-Oxo-1,4-dihydro-quinoline-3-carboxamides as BACE-1 inhibitors: synthesis, biological evaluation and docking studies. Eur. J. Med. Chem. 79, 413–421. 10.1016/j.ejmech.2014.04.025 24763262

[B132] Liu Q. Qiang X. Li Y. Sang Z. Li Y. Tan Z. (2015). Design, synthesis and evaluation of chromone-2-carboxamido-alkylbenzylamines as multifunctional agents for the treatment of Alzheimer’s disease. Bioorg Med. Chem. 23, 911–923. 10.1016/j.bmc.2015.01.042 25678013

[B133] Liu Y. Nguyen M. Robert A. Meunier B. (2019). Metal ions in alzheimer’s disease: a key role or not? Acc. Chem. Res. 52, 2026–2035. 10.1021/acs.accounts.9b00248 31274278

[B134] Loh Z. H. Kwong H. C. Lam K. W. Teh S. S. Ee G. C. L. Quah C. K. (2021). New 3-O-substituted xanthone derivatives as promising acetylcholinesterase inhibitors. J. Enzyme Inhib. Med. Chem. 36, 627–639. 10.1080/14756366.2021.1882452 33557647 PMC8759733

[B135] Lu L. Yu X. Cai Y. Sun M. Yang H. (2021). Application of CRISPR/Cas9 in alzheimer’s disease. Front. Neurosci. 15. 10.3389/fnins.2021.803894 34992519 PMC8724030

[B136] Luo W. Lv J. W. Wang T. Zhang Z. Y. Guo H. Y. Song Z. Y. (2020). Synthesis, *in vitro* and *in vivo* biological evaluation of novel graveolinine derivatives as potential anti-Alzheimer agents. Bioorg Med. Chem. 28, 115190. 10.1016/j.bmc.2019.115190 31744779

[B137] Ma F. Du H. (2017). Novel deoxyvasicinone derivatives as potent multitarget-directed ligands for the treatment of Alzheimer’s disease: design, synthesis, and biological evaluation. Eur. J. Med. Chem. 140, 118–127. 10.1016/j.ejmech.2017.09.008 28923380

[B138] Machaa B. Kulkarni R. Bagul C. Garige A. Akkinepally R. R. Garlapati A. (2021). Molecular hybridization based design and synthesis of new benzo[5,6]chromeno[2,3-b]-quinolin-13(14H)-one analogs as cholinesterase inhibitors. Med. Chem. Res. 30, 685–701. 10.1007/s00044-020-02670-w

[B139] Mahdavi M. Hariri R. Mirfazli S. S. Lotfian H. Rastergari A. Firuzi O. (2019). Synthesis and biological activity of some benzochromenoquinolinones: tacrine analogs as potent anti-alzheimer’s agents. Chem. Biodivers. 16, e1800488. 10.1002/cbdv.201800488 30720917

[B140] Mann D. M. Yates P. O. Hawkes J. (1982). The noradrenergic system in Alzheimer and multi-infarct dementias. J. Neurol. Neurosurg. Psychiatry 45, 113–119. 10.1136/jnnp.45.2.113 7069423 PMC1083037

[B141] Manzoor S. Prajapati S. Majumdar S. Raza K. Gabr M. Kumar S. (2021). Discovery of new phenyl sulfonyl-pyrimidine carboxylate derivatives as the potential multi-target drugs with effective anti-Alzheimer’s action: design, Synthesis, Crystal structure and In-vitro biological evaluation. Eur. J. Med. Chem. 215, 113224. 10.1016/j.ejmech.2021.113224 33582578

[B142] Mao F. Huang L. Luo Z. Liu A. Lu C. Xie Z. (2012). O-Hydroxyl- or o-amino benzylamine-tacrine hybrids: multifunctional biometals chelators, antioxidants, and inhibitors of cholinesterase activity and amyloid-β aggregation. Bioorg Med. Chem. 20, 5884–5892. 10.1016/j.bmc.2012.07.045 22944335

[B143] Maqbool M. Manral A. Jameel E. Kumar J. Saini V. Shandilya A. (2016). Development of cyanopyridine-triazine hybrids as lead multitarget anti-Alzheimer agents. Bioorg Med. Chem. 24, 2777–2788. 10.1016/j.bmc.2016.04.041 27157006

[B144] Marambaud P. Wilk S. Checler F. (1996). Protein kinase A phosphorylation of the proteasome: a contribution to the α-secretase pathway in human cells. J. Neurochem. 67, 2616–2619. 10.1046/j.1471-4159.1996.67062616.x 8931498

[B145] Masters C. L. Simms G. Weinman N. A. Multhaup G. McDonald B. L. Beyreuther K. (1985). Amyloid plaque core protein in Alzheimer disease and Down syndrome. Proc. Natl. Acad. Sci. U. S. A. 82, 4245–4249. 10.1073/pnas.82.12.4245 3159021 PMC397973

[B146] Masuho I. Chavali S. Muntean B. S. Skamangas N. K. Simonyan K. Patil D. N. (2018). Molecular deconvolution platform to establish disease mechanisms by surveying GPCR signaling. Cell Rep. 24, 557–568. 10.1016/j.celrep.2018.06.080 30021154 PMC6077248

[B147] Mdawar B. Ghossoub E. Khoury R. (2020). Selective serotonin reuptake inhibitors and Alzheimer’s disease. Neural Regen. Res. 15, 41–46. 10.4103/1673-5374.264445 31535641 PMC6862425

[B148] Melesina J. Simoben C. V. Praetorius L. Bülbül E. F. Robaa D. Sippl W. (2021). Strategies to design selective histone deacetylase inhibitors. ChemMedChem 16, 1336–1359. 10.1002/cmdc.202000934 33428327

[B149] Messori L. Camarri M. Ferraro T. Gabbiani C. Franceschini D. (2013). Promising *in vitro* anti-alzheimer properties for a ruthenium(III) complex. ACS Med. Chem. Lett. 4, 329–332. 10.1021/ml3003567 24900669 PMC4027362

[B150] Mezeiova E. Janockova J. Andrys R. Soukup O. Kobrlova T. Muckova L. (2021). 2-Propargylamino-naphthoquinone derivatives as multipotent agents for the treatment of Alzheimer’s disease. Eur. J. Med. Chem. 211, 113112. 10.1016/j.ejmech.2020.113112 33360800

[B151] Miao S. X. Wan L. X. He Z. X. Zhou X. L. Li X. Gao F. (2021). Pd-catalyzed direct diversification of natural anti-alzheimer’s disease drug: synthesis and biological evaluation of N-aryl huperzine A analogues. J. Nat. Prod. 84, 2374–2379. 10.1021/acs.jnatprod.1c00600 34445873

[B152] Mo J. Chen T. Yang H. Guo Y. Li Q. Qiao Y. (2020). Design, synthesis, *in vitro* and *in vivo* evaluation of benzylpiperidine-linked 1,3-dimethylbenzimidazolinones as cholinesterase inhibitors against Alzheimer’s disease. J. Enzyme Inhib. Med. Chem. 35, 330–343. 10.1080/14756366.2019.1699553 31856607 PMC6968383

[B153] Moftah H. K. Mousa M. H. A. Elrazaz E. Z. Kamel A. S. Lasheen D. S. Georgey H. H. (2024). Novel quinazolinone Derivatives: design, synthesis and *in vivo* evaluation as potential agents targeting Alzheimer disease. Bioorg Chem. 143, 107065. 10.1016/j.bioorg.2023.107065 38150939

[B154] Mollazadeh M. Mohammadi-Khanaposhtani M. Azizian H. Zonouzi A. Abdolahi Z. Nadri H. (2020). Design and synthesis of 2,4-dioxochroman-pyridinium-phenylacetamide derivatives as new anti-Alzheimer agents: *in vitro* and *in silico* studies. J. Chin. Chem. Soc. 67, 1910–1928. 10.1002/jccs.202000013

[B155] Munj S. Patil P. (2022). Drug discovery to drug development of BACE1 inhibitor as antialzheimer’s: a review. Curr. Top. Med. Chem. 23. 10.2174/1568026623666221228140450 36579387

[B156] Muntean B. S. Zucca S. MacMullen C. M. Dao M. T. Johnston C. Iwamoto H. (2018). Interrogating the spatiotemporal landscape of neuromodulatory GPCR signaling by real-time imaging of cAMP in intact neurons and circuits. Cell Rep. 22, 255–268. 10.1016/j.celrep.2017.12.022 29298426 PMC5761078

[B157] Muntean B. S. Dao M. T. Martemyanov K. A. (2019). Allostatic changes in the cAMP system drive opioid-induced adaptation in striatal dopamine signaling. Cell Rep. 29, 946–960. 10.1016/j.celrep.2019.09.034 31644915 PMC6871051

[B158] Muntean B. S. Masuho I. Dao M. Sutton L. P. Zucca S. Iwamoto H. (2021). Gαo is a major determinant of cAMP signaling in the pathophysiology of movement disorders. Cell Rep. 34. 10.1016/j.celrep.2021.108718 33535037 PMC7903328

[B159] Murtaza S. Kausar N. Arshad U. Ahmed S. Tatheer A. Najeeb J. (2022). Novel 2-aminobenzohydrazide derivatives, design, synthesis, anti-Alzheimer evaluation, SAR studies and molecular docking analysis. J. Mol. Struct. 1264. 10.1016/j.molstruc.2022.133271

[B160] Najafi Z. Saeedi M. Mahdavi M. Sabourian R. Khanavi M. Tehrani M. B. (2016). Design and synthesis of novel anti-Alzheimer’s agents: acridine-chromenone and quinoline-chromenone hybrids. Bioorg Chem. 67, 84–94. 10.1016/j.bioorg.2016.06.001 27289559

[B161] Najafi Z. Mahdavi M. Saeedi M. Karimpour-Razkenari E. Asatouri R. Vafadarnejad F. (2017). Novel tacrine-1,2,3-triazole hybrids: *in vitro*, *in vivo* biological evaluation and docking study of cholinesterase inhibitors. Eur. J. Med. Chem. 125, 1200–1212. 10.1016/j.ejmech.2016.11.008 27863370

[B162] Naseri N. N. Wang H. Guo J. Sharma M. Luo W. (2019). The complexity of tau in Alzheimer’s disease. Neurosci. Lett. 705, 183–194. 10.1016/j.neulet.2019.04.022 31028844 PMC7060758

[B163] Neumann U. Ufer M. Jacobson L. H. Rouzade‐Dominguez M. Huledal G. Kolly C. (2018). The BACE ‐1 inhibitor CNP 520 for prevention trials in Alzheimer’s disease. EMBO Mol. Med. 10. 10.15252/emmm.201809316 30224383 PMC6220303

[B164] Nieto-Estevez V. Changarathil G. Adeyeye A. O. Coppin M. O. Kassim R. S. Zhu J. (2022). HDAC1 regulates neuronal differentiation. Front. Mol. Neurosci. 14. 10.3389/fnmol.2021.815808 35095417 PMC8789757

[B165] Ning C. Mo L. Chen X. Tu W. Wu J. Hou S. (2018). Triptolide derivatives as potential multifunctional anti-Alzheimer agents: synthesis and structure–activity relationship studies. Bioorg Med. Chem. Lett. 28, 689–693. 10.1016/j.bmcl.2018.01.019 29366650

[B166] Nowak K. Lange-Dohna C. Zeitschel U. Günther A. Lüscher B. Robitzki A. (2006). The transcription factor Yin Yang 1 is an activator of BACE1 expression. J. Neurochem. 96, 1696–1707. 10.1111/j.1471-4159.2006.03692.x 16539685

[B167] Nussbaum J. M. Schilling S. Cynis H. Silva A. Swanson E. Wangsanut T. (2012). Prion-like behaviour and tau-dependent cytotoxicity of pyroglutamylated amyloid-β. Nature 485, 651–655. 10.1038/nature11060 22660329 PMC3367389

[B168] Obermayer J. Verhoog M. B. Luchicchi A. Mansvelder H. D. (2017). Cholinergic modulation of cortical microcircuits is layer-specific: evidence from rodent, monkey and human brain. Front. Neural Circuits 11, 100. 10.3389/fncir.2017.00100 29276477 PMC5727016

[B169] Oset-Gasque M. J. González M. P. Pérez-Peña J. García-Font N. Romero A. Pino J. D. (2014). Toxicological and pharmacological evaluation, antioxidant, ADMET and molecular modeling of selected racemic chromenotacrines {11-amino-12-aryl-8,9, 10,12-tetrahydro-7H-chromeno[2,3-b]quinolin-3-ols} for the potential prevention and treatment of Alzheimer’s disease. Eur. J. Med. Chem. 74, 491–501. 10.1016/j.ejmech.2013.12.021 24502897

[B170] Özdemir Z. Yılmaz H. Sarı S. Karakurt A. Şenol F. S. Uysal M. (2017). Design, synthesis, and molecular modeling of new 3(2H)-pyridazinone derivatives as acetylcholinesterase/butyrylcholinesterase inhibitors. Med. Chem. Res. 26, 2293–2308. 10.1007/s00044-017-1930-x

[B171] Pagoni A. Marinelli L. Di Stefano A. Ciulla M. Turkez H. Mardinoglu A. (2020). Novel anti-Alzheimer phenol-lipoyl hybrids: synthesis, physico-chemical characterization, and biological evaluation. Eur. J. Med. Chem. 186, 111880. 10.1016/j.ejmech.2019.111880 31753513

[B172] Pal T. Bhimaneni S. Sharma A. Flora S. J. S. (2020). Design, synthesis, biological evaluation and molecular docking study of novel pyridoxine-triazoles as anti-Alzheimer’s agents. RSC Adv. 10, 26006–26021. 10.1039/d0ra04942e 35519785 PMC9055346

[B173] Palacios-Filardo J. Mellor J. R. (2019). Neuromodulation of hippocampal long-term synaptic plasticity. Curr. Opin. Neurobiol. 54, 37–43. 10.1016/j.conb.2018.08.009 30212713 PMC6367596

[B174] Pandolfi F. De Vita D. Bortolami M. Coluccia A. Di Santo R. Costi R. (2017). New pyridine derivatives as inhibitors of acetylcholinesterase and amyloid aggregation. Eur. J. Med. Chem. 141, 197–210. 10.1016/j.ejmech.2017.09.022 29031067

[B175] Panek D. Wiȩckowska A. Jończyk J. Godyń J. Bajda M. Wichur T. (2018). Design, synthesis, and biological evaluation of 1-Benzylamino-2-hydroxyalkyl derivatives as new potential disease-modifying multifunctional anti-alzheimer’s agents. ACS Chem. Neurosci. 9, 1074–1094. 10.1021/acschemneuro.7b00461 29345897

[B176] Pant S. Kumar K R. Rana P. Anthwal T. Ali S. M. Gupta M. (2024). Novel substituted pyrimidine derivatives as potential anti-alzheimer’s agents: synthesis, biological, and molecular docking studies. ACS Chem. Neurosci. 15, 783–797. 10.1021/acschemneuro.3c00662 38320262

[B177] Parlar S. Sayar G. Tarikogullari A. H. Karadagli S. S. Alptuzun V. Erciyas E. (2019). Synthesis, bioactivity and molecular modeling studies on potential anti-Alzheimer piperidinehydrazide-hydrazones. Bioorg Chem. 87, 888–900. 10.1016/j.bioorg.2018.11.051 30538051

[B178] Pasieka A. Panek D. Szałaj N. Espargaró A. Wiȩckowska A. Malawska B. (2021). Dual inhibitors of amyloid-β and tau aggregation with amyloid-β disaggregating properties: extended in cellulo, *in silico*, and kinetic studies of multifunctional anti-alzheimer’s agents. ACS Chem. Neurosci. 12, 2057–2068. 10.1021/acschemneuro.1c00235 34019757 PMC8291496

[B179] Patel D. V. Patel N. R. Kanhed A. M. Patel S. P. Sinha A. Kansara D. D. (2019). Novel multitarget directed triazinoindole derivatives as anti-alzheimer agents. ACS Chem. Neurosci. 10, 3635–3661. 10.1021/acschemneuro.9b00226 31310717

[B180] Patel D. V. Patel N. R. Kanhed A. M. Teli D. M. Patel K. B. Joshi P. D. (2020). Novel carbazole-stilbene hybrids as multifunctional anti-Alzheimer agents. Bioorg Chem. 101, 103977. 10.1016/j.bioorg.2020.103977 32485470

[B181] Pathomwat W. Jiaranaikulwanitch J. Vajragupta O. Jiranusornkul S. Saenjum C. Yooin W. (2021). Potential anti-alzheimer agents from guanidinyl tryptophan derivatives with activities of membrane adhesion and conformational transition inhibitions. Molecules 26, 4863. 10.3390/molecules26164863 34443456 PMC8398955

[B182] Paudel P. Seong S. H. Zhou Y. Park H. J. Jung H. A. Choi J. S. (2019). Anti-Alzheimer’s disease activity of bromophenols from a red alga, Symphyocladia latiuscula (harvey) Yamada. ACS Omega 4, 12259–12270. 10.1021/acsomega.9b01557 31460342 PMC6682041

[B183] Pérez-Areales F. J. Garrido M. Aso E. Bartolini M. De Simone A. Espargaró A. (2020). Centrally active multitarget anti-alzheimer agents derived from the antioxidant lead CR-6. J. Med. Chem. 63, 9360–9390. 10.1021/acs.jmedchem.0c00528 32706255

[B184] Peters M. Mizuno K. Ris L. Angelo M. Godaux E. Giese K. P. (2003). Loss of Ca2+/calmodulin kinase kinase beta affects the formation of some, but not all, types of hippocampus-dependent long-term memory. J. Neurosci. 23, 9752–9760. 10.1523/JNEUROSCI.23-30-09752.2003 14586002 PMC6740881

[B185] Picciotto M. R. Higley M. J. Mineur Y. S. (2012). Acetylcholine as a neuromodulator: cholinergic signaling shapes nervous system function and behavior. Neuron 76, 116–129. 10.1016/j.neuron.2012.08.036 23040810 PMC3466476

[B186] Qiang X. Sang Z. Yuan W. Li Y. Liu Q. Bai P. (2014). Design, synthesis and evaluation of genistein-O-alkylbenzylamines as potential multifunctional agents for the treatment of Alzheimer’s disease. Eur. J. Med. Chem. 76, 314–331. 10.1016/j.ejmech.2014.02.045 24589487

[B187] Qiu C. Kivipelto M. Von Strauss E. (2009). Epidemiology of Alzheimer’s disease: occurrence, determinants, and strategies toward intervention. Dialogues Clin. Neurosci. 11, 111–128. 10.31887/DCNS.2009.11.2/cqiu 19585947 PMC3181909

[B188] Ramanan M. Sinha S. Sudarshan K. Aidhen I. S. Doble M. (2016). Inhibition of the enzymes in the leukotriene and prostaglandin pathways in inflammation by 3-aryl isocoumarins. Eur. J. Med. Chem. 124, 428–434. 10.1016/j.ejmech.2016.08.066 27597418

[B189] Reis J. Cagide F. Valencia M. E. Teixeira J. Bagetta D. Pérez C. (2018). Multi-target-directed ligands for Alzheimer’s disease: discovery of chromone-based monoamine oxidase/cholinesterase inhibitors. Eur. J. Med. Chem. 158, 781–800. 10.1016/j.ejmech.2018.07.056 30245401

[B190] Ricardo S. Lehmann R. (2009). An ABC transporter controls export of a Drosophila germ cell attractant. Sci. (1979) 323, 943–946. 10.1126/science.1166239 19213920 PMC2729540

[B191] Roldán-Peña J. M. Alejandre-Ramos D. López Ó. Maya I. Lagunes I. Padrón J. M. (2017). New tacrine dimers with antioxidant linkers as dual drugs: anti-Alzheimer’s and antiproliferative agents. Eur. J. Med. Chem. 138, 761–773. 10.1016/j.ejmech.2017.06.048 28728108

[B192] Romeiro L. A. S. Da Costa Nunes J. L. De Oliveira Miranda C. Simoies Heyn Roth Cardoso G. De Oliveira A. S. Gandini A. (2019). Novel sustainable-by-design HDAC inhibitors for the treatment of alzheimer’s disease. ACS Med. Chem. Lett. 10, 671–676. 10.1021/acsmedchemlett.9b00071 30996816 PMC6466821

[B193] Rook Y. Schmidtke K. U. Gaube F. Schepmann D. Wünsch B. Heilmann J. (2010). Bivalent β-carbolines as potential multitarget anti-alzheimer agents. J. Med. Chem. 53, 3611–3617. 10.1021/jm1000024 20361801

[B194] Rosini M. Andrisano V. Bartolini M. Bolognesi M. L. Hrelia P. Minarini A. (2005). Rational approach to discover multipotent anti-Alzheimer drugs. J. Med. Chem. 48, 360–363. 10.1021/jm049112h 15658850

[B195] Rozzini L. Costardi D. Chilovi V. Franzoni S. Trabucchi M. Padovani A. (2007). Efficacy of cognitive rehabilitation in patients with mild cognitive impairment treated with cholinesterase inhibitors. Int. J. Geriatr. Psychiatry 22, 356–360. 10.1002/gps.1681 17117398

[B196] Sadeghian B. Sakhteman A. Faghih Z. Nadri H. Edraki N. Iraji A. (2020). Design, synthesis and biological activity evaluation of novel carbazole-benzylpiperidine hybrids as potential anti Alzheimer agents. J. Mol. Struct. 1221. 10.1016/j.molstruc.2020.128793

[B197] Sadleir K. R. Kandalepas P. C. Buggia-Prévot V. Nicholson D. A. Thinakaran G. Vassar R. (2016). Presynaptic dystrophic neurites surrounding amyloid plaques are sites of microtubule disruption, BACE1 elevation, and increased Aβ generation in Alzheimer’s disease. Acta Neuropathol. 132, 235–256. 10.1007/s00401-016-1558-9 26993139 PMC4947125

[B198] Saeedi M. Felegari P. Iraji A. Hariri R. Rastegari A. Mirfazli S. S. (2021). Novel N-benzylpiperidine derivatives of 5-arylisoxazole-3-carboxamides as anti-Alzheimer’s agents. Arch. Pharm. Weinh. 354, e2000258. 10.1002/ardp.202000258 33226157

[B199] Saha R. N. Pahan K. (2006). HATs and HDACs in neurodegeneration: a tale of disconcerted acetylation homeostasis. Cell Death Differ. 13, 539–550. 10.1038/sj.cdd.4401769 16167067 PMC1963416

[B200] Salloway S. Pain A. Lee E. Papka M. Ferguson M. Wang H. (2025). TRAILBLAZER‐ALZ 4: a phase 3 trial comparing donanemab with aducanumab on amyloid plaque clearance in early, symptomatic Alzheimer’s disease. Alzheimer’s and Dementia 21. 10.1002/alz.70293 40390253 PMC12089073

[B201] Sambamurti K. Kinsey R. Maloney B. Ge Y.-W. Lahiri D. K. (2004). Gene structure and organization of the human β‐secretase (BACE) promoter. FASEB J. 18, 1034–1036. 10.1096/fj.03-1378fje 15059975

[B202] Sanchez M. Blower S. (1997). Uncertainty and sensitivity analysis of the basic reproductive rate: tuberculosis as an example. Am. J. Epidemiol. 145, 1127–1137. 10.1093/oxfordjournals.aje.a009076 9199543

[B203] Sang Z. Pan W. Wang K. Ma Q. Yu L. Yang Y. (2017a). Design, synthesis and evaluation of novel ferulic acid-O-alkylamine derivatives as potential multifunctional agents for the treatment of Alzheimer’s disease. Eur. J. Med. Chem. 130, 379–392. 10.1016/j.ejmech.2017.02.039 28279845

[B204] Sang Z. Qiang X. Li Y. Xu R. Cao Z. Song Q. (2017b). Design, synthesis and evaluation of scutellarein-O-acetamidoalkylbenzylamines as potential multifunctional agents for the treatment of Alzheimer’s disease. Eur. J. Med. Chem. 135, 307–323. 10.1016/j.ejmech.2017.04.054 28458136

[B205] Sang Z. Wang K. Wang H. Wang H. Ma Q. Han X. (2017c). Design, synthesis and biological evaluation of 2-acetyl-5-O-(amino-alkyl)phenol derivatives as multifunctional agents for the treatment of Alzheimer’s disease. Bioorg Med. Chem. Lett. 27, 5046–5052. 10.1016/j.bmcl.2017.09.057 29033233

[B206] Sang Z. Wang K. Wang H. Yu L. Wang H. Ma Q. (2017d). Design, synthesis and biological evaluation of phthalimide-alkylamine derivatives as balanced multifunctional cholinesterase and monoamine oxidase-B inhibitors for the treatment of Alzheimer’s disease. Bioorg Med. Chem. Lett. 27, 5053–5059. 10.1016/j.bmcl.2017.09.055 29033232

[B207] Sang Z. Song Q. Cao Z. Deng Y. Tan Z. Zhang L. (2021). Design, synthesis and evaluation of novel dimethylamino chalcone-O-alkylamines derivatives as potential multifunctional agents against Alzheimer’s disease. Eur. J. Med. Chem. 216, 113310. 10.1016/j.ejmech.2021.113310 33667847

[B208] Sanjay Shin J. H. Park M. Lee H. J. (2022). Cyanidin-3-O-Glucoside regulates the M1/M2 polarization of microglia via PPARγ and Aβ42 phagocytosis through TREM2 in an alzheimer’s disease model. Mol. Neurobiol. 59, 5135–5148. 10.1007/s12035-022-02873-9 35670898 PMC9363298

[B209] Santana D. A. Smith M. de A. C. Chen E. S. (2023). Histone modifications in alzheimer’s disease. Genes (Basel) 14, 347. 10.3390/genes14020347 36833274 PMC9956192

[B210] Scheide M. R. Schneider A. R. Jardim G. A. M. Martins G. M. Durigon D. C. Saba S. (2020). Electrochemical synthesis of selenyl-dihydrofurans via anodic selenofunctionalization of allyl-naphthol/phenol derivatives and their anti-Alzheimer activity. Org. Biomol. Chem. 18, 4916–4921. 10.1039/d0ob00629g 32353091

[B211] Scheiderer C. L. McCutchen E. Thacker E. E. Kolasa K. Ward M. K. Parsons D. (2006). Sympathetic sprouting drives hippocampal cholinergic reinnervation that prevents loss of a muscarinic receptor-dependent long-term depression at CA3-CA1 synapses. J. Neurosci. 26, 3745–3756. 10.1523/JNEUROSCI.5507-05.2006 16597728 PMC6674126

[B212] Schilling S. Lindner C. Koch B. Wermann M. Rahfeld J. U. Von Bohlen A. (2007). Isolation and characterization of glutaminyl cyclases from Drosophila: evidence for enzyme forms with different subcellular localization. Biochemistry 46, 10921–10930. 10.1021/bi701043x 17722885

[B213] Scott J. D. Li S. W. Brunskill A. P. J. Chen X. Cox K. Cumming J. N. (2016). Discovery of the 3-Imino-1,2,4-thiadiazinane 1,1-dioxide derivative verubecestat (MK-8931)–A β-site amyloid precursor protein cleaving enzyme 1 inhibitor for the treatment of alzheimer’s disease. J. Med. Chem. 59, 10435–10450. 10.1021/acs.jmedchem.6b00307 27933948

[B214] Seto E. Yoshida M. (2014). Erasers of histone acetylation: the histone deacetylase enzymes. Cold Spring Harb. Perspect. Biol. 6, a018713. 10.1101/cshperspect.a018713 24691964 PMC3970420

[B215] Shahrivar-Gargari M. Hamzeh-Mivehroud M. Hemmati S. Mojarrad J. S. Tüylü Küçükkılınç T. Ayazgök B. (2021). Hybridization-based design of novel anticholinesterase indanone–carbamates for Alzheimer’s disease: synthesis, biological evaluation, and docking studies. Arch. Pharm. Weinh. 354, 2000453. 10.1002/ardp.202000453 33872422

[B216] Shaik J. B. Yeggoni D. P. Kandrakonda Y. R. Penumala M. Zinka R. B. Kotapati K. V. (2019). Synthesis and biological evaluation of flavone-8-acrylamide derivatives as potential multi-target-directed anti Alzheimer agents and investigation of binding mechanism with acetylcholinesterase. Bioorg Chem. 88, 102960. 10.1016/j.bioorg.2019.102960 31102808

[B217] Sharma P. Tripathi A. Tripathi P. N. Prajapati S. K. Seth A. Tripathi M. K. (2019). Design and development of multitarget-directed N-Benzylpiperidine analogs as potential candidates for the treatment of Alzheimer’s disease. Eur. J. Med. Chem. 167, 510–524. 10.1016/j.ejmech.2019.02.030 30784883

[B218] Shi M. Chu F. Zhu F. Zhu J. (2022). Impact of anti-amyloid-β monoclonal antibodies on the pathology and clinical profile of alzheimer’s disease: a focus on aducanumab and Lecanemab. Front. Aging Neurosci. 14. 10.3389/fnagi.2022.870517 35493943 PMC9039457

[B219] Shidore M. Machhi J. Shingala K. Murumkar P. Sharma M. K. Agrawal N. (2016). Benzylpiperidine-linked diarylthiazoles as potential anti-alzheimer’s agents: synthesis and biological evaluation. J. Med. Chem. 59, 5823–5846. 10.1021/acs.jmedchem.6b00426 27253679

[B220] Shrivastava S. K. Srivastava P. Upendra T. V. R. Tripathi P. N. Sinha S. K. (2017). Design, synthesis and evaluation of some N-methylenebenzenamine derivatives as selective acetylcholinesterase (AChE) inhibitor and antioxidant to enhance learning and memory. Bioorg. and Med. Chem. 25, 1471–1480. 10.1016/j.bmc.2017.01.010 28126439

[B221] Shukla S. Tekwani B. L. (2020). Histone deacetylases inhibitors in neurodegenerative diseases, neuroprotection and neuronal differentiation. Front. Pharmacol. 11, 537. 10.3389/fphar.2020.00537 32390854 PMC7194116

[B222] Singh M. Kaur M. Vyas B. Silakari O. (2018a). Design, synthesis and biological evaluation of 2-Phenyl-4H-chromen-4-one derivatives as polyfunctional compounds against Alzheimer’s disease. Med. Chem. Res. 27, 520–530. 10.1007/s00044-017-2078-4

[B223] Singh R. Thota S. Bansal R. (2018b). Studies on 16,17-pyrazoline substituted heterosteroids as anti-alzheimer and anti-parkinsonian agents using LPS induced neuroinflammation models of mice and rats. ACS Chem. Neurosci. 9, 272–283. 10.1021/acschemneuro.7b00303 29019394

[B224] Singh N. Benoit M. R. Zhou J. Das B. Davila-Velderrain J. Kellis M. (2022). BACE-1 inhibition facilitates the transition from homeostatic microglia to DAM-1. Sci. Adv. 8, eabo1286. 10.1126/sciadv.abo1286 35714196 PMC9205595

[B225] Singh A. Kaur K. Arora S. Sharma A. Singh K. Mohana P. (2025). Development of coumarin-inspired bifunctional hybrids as a new class of anti-Alzheimer’s agents with potent *in vivo* efficacy. RSC Med. Chem. 16. 10.1039/D4MD00782D 39790122 PMC11707525

[B226] Sirimangkalakitti N. Juliawaty L. D. Hakim E. H. Waliana I. Saito N. Koyama K. (2019). Naturally occurring biflavonoids with amyloid β aggregation inhibitory activity for development of anti-Alzheimer agents. Bioorg Med. Chem. Lett. 29, 1994–1997. 10.1016/j.bmcl.2019.05.020 31138471

[B227] Siwecka N. Saramowicz K. Galita G. Rozpędek-Kamińska W. Majsterek I. (2023). Inhibition of protein aggregation and endoplasmic reticulum stress as a targeted therapy for α-Synucleinopathy. Pharmaceutics 15. 10.3390/pharmaceutics15082051 37631265 PMC10459316

[B228] Spilovska Chalupova K. Korabecny J. Kral J. Horova A. Musilek K. Soukup O. (2013). 7-Methoxytacrine-Adamantylamine heterodimers as cholinesterase inhibitors in alzheimer’s disease treatment — synthesis, biological evaluation and molecular modeling studies. Molecules 18, 2397–2418. 10.3390/molecules18022397 23429378 PMC6270602

[B229] Spilovska K. Korabecny J. Horova A. Musilek K. Nepovimova E. Drtinova L. (2015). Design, synthesis and *in vitro* testing of 7-methoxytacrine-amantadine analogues: a novel cholinesterase inhibitors for the treatment of Alzheimer’s disease. Med. Chem. Res. 24, 2645–2655. 10.1007/s00044-015-1316-x

[B230] Sreenivasmurthy S. G. Iyaswamy A. Krishnamoorthi S. Senapati S. Malampati S. Zhu Z. (2022). Protopine promotes the proteasomal degradation of pathological tau in Alzheimer’s disease models via HDAC6 inhibition. Phytomedicine 96, 153887. 10.1016/j.phymed.2021.153887 34936968

[B231] Sudarshan K. Boda A. K. Dogra S. Bose I. Yadav P. N. Aidhen I. S. (2019). Discovery of an isocoumarin analogue that modulates neuronal functions via neurotrophin receptor TrkB. Bioorg Med. Chem. Lett. 29, 585–590. 10.1016/j.bmcl.2018.12.057 30600206

[B232] Sukumarapillai D. K. Kooi-Yeong K. Kia Y. Murugaiyah V. Iyer S. K. (2016). Design, synthesis and cholinesterase inhibitory evaluation study of fluorescent N-benzylpiperidine-4-one derivatives. Med. Chem. Res. 25, 1705–1715. 10.1007/s00044-016-1619-6

[B233] Sumi T. Harada K. (2023). Muscarinic acetylcholine receptor-dependent and NMDA receptor-dependent LTP and LTD share the common AMPAR trafficking pathway. iScience 26. 10.1016/j.isci.2023.106133 36866246 PMC9972575

[B234] Sutton L. P. Muntean B. S. Ostrovskaya O. Zucca S. Dao M. Orlandi C. (2019). NF1-cAMP signaling dissociates cell type–specific contributions of striatal medium spiny neurons to reward valuation and motor control. PLoS Biol. 17, e3000477. 10.1371/journal.pbio.3000477 31600280 PMC6805008

[B235] Suwanhom P. Nualnoi T. Khongkow P. Sanghiran Lee V. Lomlim L. (2020). Synthesis and evaluation of chromone-2-carboxamido-alkylamines as potent acetylcholinesterase inhibitors. Med. Chem. Res. 29, 564–574. 10.1007/s00044-020-02508-5

[B236] Suzuki T. Miura M. Nishimura K.-Y. Aosaki T. (2001). Dopamine-dependent synaptic plasticity in the striatal cholinergic interneurons. J. Neurosci. 21, 6492–6501. 10.1523/JNEUROSCI.21-17-06492.2001 11517238 PMC6763115

[B237] Świt P. Pollap A. Orzeł J. (2023). Spectroscopic determination of acetylcholine (ACh): a representative review. Top. Curr. Chem. 381. 10.1007/s41061-023-00426-9 37169979 PMC10175388

[B238] Taha M. Alshamrani F. J. Rahim F. Anouar E. H. Uddin N. Chigurupati S. (2021). Synthesis, characterization, biological evaluation, and kinetic study of indole base sulfonamide derivatives as acetylcholinesterase inhibitors in search of potent anti-Alzheimer agent. J. King Saud. Univ. Sci. 33, 101401. 10.1016/j.jksus.2021.101401

[B239] Tamagno E. Guglielmotto M. Monteleone D. Vercelli A. Tabaton M. (2012). Transcriptional and post-transcriptional regulation of β-secretase. IUBMB Life 64, 943–950. 10.1002/iub.1099 23180460

[B240] Tehrani M. B. Rezaei Z. Asadi M. Behnammanesh H. Nadri H. Afsharirad F. (2019). Design, synthesis, and cholinesterase inhibition assay of coumarin-3-carboxamide-N-morpholine hybrids as new anti-alzheimer agents. Chem. Biodivers. 16, e1900144. 10.1002/cbdv.201900144 31155827

[B241] Thathiah A. De Strooper B. (2011). The role of G protein-coupled receptors in the pathology of Alzheimer’s disease. Nat. Rev. Neurosci. 12, 73–87. 10.1038/nrn2977 21248787

[B242] Thijssen E. H. La Joie R. Strom A. Fonseca C. Iaccarino L. Wolf A. (2021). Plasma phosphorylated tau 217 and phosphorylated tau 181 as biomarkers in Alzheimer’s disease and frontotemporal lobar degeneration: a retrospective diagnostic performance study. Lancet Neurol. 20, 739–752. 10.1016/S1474-4422(21)00214-3 34418401 PMC8711249

[B243] Thomas E. A. Coppola G. Desplats P. A. Tang B. Soragni E. Burnett R. (2008). The HDAC inhibitor 4b ameliorates the disease phenotype and transcriptional abnormalities in Huntington’s disease transgenic mice. Proc. Natl. Acad. Sci. U. S. A. 105, 15564–15569. 10.1073/pnas.0804249105 18829438 PMC2563081

[B244] Ţînţaş M. L. Azzouz R. Peauger L. Gembus V. Petit E. Bailly L. (2018). Access to highly enantioenriched donepezil-like 1,4-dihydropyridines as promising anti-alzheimer Prodrug candidates via Enantioselective Tsuji Allylation and organocatalytic aza-ene-type domino reactions. J. Org. Chem. 83, 10231–10240. 10.1021/acs.joc.8b01442 30004228

[B245] Titov A. A. Kobzev M. S. Catto M. de Candia M. Gambacorta N. Denora N. (2021). Away from flatness: unprecedented nitrogen-bridged cyclopenta[ a]indene derivatives as novel anti-alzheimer multitarget agents. ACS Chem. Neurosci. 12, 340–353. 10.1021/acschemneuro.0c00706 33395258

[B246] Trinh P. N. H. Baltos J. A. Hellyer S. D. May L. T. Gregory K. J. (2022). Adenosine receptor signalling in Alzheimer’s disease. Purinergic Signal 18, 359–381. 10.1007/s11302-022-09883-1 35870032 PMC9391555

[B247] Tripathi R. K. P. Ayyannan S. R. (2018). Evaluation of 2-amino-6-nitrobenzothiazole derived hydrazones as acetylcholinesterase inhibitors: *in vitro* assays, molecular docking and theoretical ADMET prediction. Med. Chem. Res. 27, 709–725. 10.1007/s00044-017-2095-3

[B248] Tuylu Kucukkilinc T. Safari Yanghagh K. Ayazgok B. Ali Roknipour M. Homayouni Moghadam F. Moradi A. (2017). Synthesis and neuroprotective activity of novel 1,2,4-triazine derivatives with ethyl acetate moiety against H2O2 and Aβ-induced neurotoxicity. Med. Chem. Res. 26, 3057–3071. 10.1007/s00044-017-2003-x

[B249] Unsal-Tan O. Ozadali-Sari K. Ayazgok B. Küçükkılınç T. T. Balkan A. (2017). Novel 2-Arylbenzimidazole derivatives as multi-targeting agents to treat Alzheimer’s disease. Med. Chem. Res. 26, 1506–1515. 10.1007/s00044-017-1874-1

[B250] van Dyck C. H. Swanson C. J. Paul A. J B. R. Christopher C. Michelle G. (2023). Lecanemab in early alzheimer’s disease. N. Engl. J. Med. 388, 9–21. 10.1056/NEJMoa2212948 36449413

[B251] Vassar R. Bennett B. D. Babu-Khan S. Kahn S. Mendiaz E. A. Denis P. (1999). Beta-secretase cleavage of Alzheimer's amyloid precursor protein by the transmembrane aspartic protease BACE. Sci. (1979) 286 (286), 735–741. 10.1126/science.286.5440.735 10531052

[B252] Verheijen J. H. Huisman L. G. M. van Lent N. Neumann U. Paganetti P. Hack C. E. (2006). Detection of a soluble form of BACE-1 in human cerebrospinal fluid by a sensitive activity assay. Clin. Chem. 52, 1168–1174. 10.1373/clinchem.2006.066720 16614000

[B253] Viayna E. Sola I. Bartolini M. De Simone A. Tapia-Rojas C. Serrano F. G. (2014). Synthesis and multitarget biological profiling of a novel family of rhein derivatives as disease-modifying anti-Alzheimer agents. J. Med. Chem. 57, 2549–2567. 10.1021/jm401824w 24568372

[B254] Volk L. J. Pfeiffer B. E. Gibson J. R. Huber K. M. (2007). Multiple Gq-coupled receptors converge on a common protein synthesis-dependent long-term depression that is affected in fragile X syndrome mental retardation. J. Neurosci. 27, 11624–11634. 10.1523/JNEUROSCI.2266-07.2007 17959805 PMC6673232

[B255] Wang B. Wang Z. Chen H. Lu C. J. Li X. (2016). Synthesis and evaluation of 8-hydroxyquinolin derivatives substituted with (benzo[d] [1,2]selenazol-3(2H)-one) as effective inhibitor of metal-induced Aβ aggregation and antioxidant. Bioorg Med. Chem. 24, 4741–4749. 10.1016/j.bmc.2016.08.017 27567080

[B256] Wang J. Cai P. Yang X. L. Li F. Wu J. J. Kong L. Y. (2017). Novel cinnamamide-dibenzylamine hybrids: potent neurogenic agents with antioxidant, cholinergic, and neuroprotective properties as innovative drugs for Alzheimer’s disease. Eur. J. Med. Chem. 139, 68–83. 10.1016/j.ejmech.2017.07.077 28800459

[B257] Wang M. Qin H. L. Leng J. Ameeduzzafar Amjad M. W. Raja M. A. G. (2018). Synthesis and biological evaluation of new tetramethylpyrazine-based chalcone derivatives as potential anti-Alzheimer agents. Chem. Biol. Drug Des. 92, 1859–1866. 10.1111/cbdd.13355 29923315

[B258] Weller J. Budson A. (2018). Current understanding of Alzheimer’s disease diagnosis and treatment. F1000Res 7. 10.12688/f1000research.14506.1 30135715 PMC6073093

[B259] Wichur T. Więckowska A. Więckowski K. Godyń J. Jończyk J. Valdivieso Á. del R. (2020). 1-Benzylpyrrolidine-3-amine-based BuChE inhibitors with anti-aggregating, antioxidant and metal-chelating properties as multifunctional agents against Alzheimer’s disease. Eur. J. Med. Chem. 187, 111916. 10.1016/j.ejmech.2019.111916 31812794

[B260] Więckowska A. Więckowski K. Bajda M. Brus B. Sałat K. Czerwińska P. (2015). Synthesis of new N-benzylpiperidine derivatives as cholinesterase inhibitors with β-amyloid anti-aggregation properties and beneficial effects on memory *in vivo* . Bioorg Med. Chem. 23, 2445–2457. 10.1016/j.bmc.2015.03.051 25868744

[B261] Wijeratne T. Andrade-Guerrero J. Santiago-Balmaseda A. Jeronimo-Aguilar P. Vargas-Rodríguez I. Ruth Cadena-Suárez A. (2023). Citation: alzheimer’s disease: an updated overview of its genetics. Int. J. Mol. Sci., 3754. 10.3390/ijms 36835161 PMC9966419

[B262] Wittnam J. L. Portelius E. Zetterberg H. Gustavsson M. K. Schilling S. Koch B. (2012). Pyroglutamate amyloid β(aβ) aggravates behavioral deficits in transgenic amyloid mouse model for Alzheimer disease. J. Biol. Chem. 287, 8154–8162. 10.1074/jbc.M111.308601 22267726 PMC3318696

[B263] Wu M. Zhang M. Yin X. Chen K. Hu Z. Zhou Q. (2021). The role of pathological tau in synaptic dysfunction in Alzheimer’s diseases. Transl. Neurodegener. 10. 10.1186/s40035-021-00270-1 34753506 PMC8579533

[B264] Xiao C. Zhou C. Y. Jiang J. H. Yin C. (2020). Neural circuits and nicotinic acetylcholine receptors mediate the cholinergic regulation of midbrain dopaminergic neurons and nicotine dependence. Acta Pharmacol. Sin. 41, 1–9. 10.1038/s41401-019-0299-4 31554960 PMC7468330

[B265] Xu Q. X. Hu Y. Li G. Y. Xu W. Zhang Y. T. Yang X. W. (2018). Multi-target anti-Alzheimer activities of four prenylated compounds from Psoralea Fructus. Molecules 23, 614. 10.3390/molecules23030614 29518051 PMC6017461

[B266] Xu X. Lü P. Wang J. Xu F. Liang L. Wang C. (2019a). Design, synthesis, and biological evaluation of 4‐aminopyrimidine or 4,6‐diaminopyrimidine derivatives as beta amyloid cleaving enzyme‐1 inhibitors. Chem. Biol. Drug Des. 93, 926–933. 10.1111/cbdd.13489 30667164

[B267] Xu Y. Zhang J. Wang H. Mao F. Bao K. Liu W. (2019b). Rational design of novel selective dual-target inhibitors of acetylcholinesterase and monoamine oxidase B as potential anti-alzheimer’s disease agents. ACS Chem. Neurosci. 10, 482–496. 10.1021/acschemneuro.8b00357 30110536

[B268] Yang H. L. Cai P. Liu Q. H. Yang X. L. Li F. Wang J. (2017). Design, synthesis and evaluation of coumarin-pargyline hybrids as novel dual inhibitors of monoamine oxidases and amyloid-β aggregation for the treatment of Alzheimer’s disease. Eur. J. Med. Chem. 138, 715–728. 10.1016/j.ejmech.2017.07.008 28728104

[B269] Yang J. Zhang P. Hu Y. Liu T. Sun J. Wang X. (2019). Synthesis and biological evaluation of 3-arylcoumarins as potential anti-Alzheimer’s disease agents. J. Enzyme Inhib. Med. Chem. 34, 651–656. 10.1080/14756366.2019.1574297 30746966 PMC6374920

[B270] Yang J. Yun Y. Miao Y. Sun J. Wang X. (2020). Synthesis and biological evaluation of 3-arylbenzofuranone derivatives as potential anti-Alzheimer’s disease agents. J. Enzyme Inhib. Med. Chem. 35, 805–814. 10.1080/14756366.2020.1740694 32183602 PMC7155212

[B271] Yang Y. Bagyinszky E. An S. S. A. (2023). Presenilin-1 (PSEN1) mutations: clinical phenotypes beyond alzheimer’s disease. Int. J. Mol. Sci. 24, 8417. 10.3390/ijms24098417 37176125 PMC10179041

[B272] Yazdani M. Edraki N. Badri R. Khoshneviszadeh M. Iraji A. Firuzi O. (2018). Multi-target inhibitors against Alzheimer disease derived from 3-hydrazinyl 1,2,4-triazine scaffold containing pendant phenoxy methyl-1,2,3-triazole: design, synthesis and biological evaluation. Bioorg Chem. 84, 363–371. 10.1016/j.bioorg.2018.11.038 30530107

[B273] Yu X. Li Y. Zou Y. Zheng Y. He Z. Liu Z. (2019). Glutaminyl cyclase inhibitor contributes to the regulation of HSP70, HSP90, actin, and ribosome on gene and protein levels *in vitro* . J. Cell Biochem. 120, 9460–9471. 10.1002/jcb.28222 30582198

[B274] Yudi Utomo R. Asawa Y. Okada S. Ban H. S. Yoshimori A. Bajorath J. (2021). Development of curcumin-based amyloid β aggregation inhibitors for Alzheimer’s disease using the SAR matrix approach. Bioorg Med. Chem. 46. 10.1016/j.bmc.2021.116357 34391121

[B275] Yun Y. Miao Y. Sun X. Sun J. Wang X. (2021). Synthesis and biological evaluation of 2-arylbenzofuran derivatives as potential anti-Alzheimer’s disease agents. J. Enzyme Inhib. Med. Chem. 36, 1346–1356. 10.1080/14756366.2021.1940993 34134572 PMC8765280

[B276] Zang Y. Liu K. Wang W. Li C. Ma J. Yang J. (2021). Claulansine F–donepezil hybrids as anti-alzheimer’s disease agents with cholinergic, free-radical scavenging, and neuroprotective activities. Molecules 26. 10.3390/molecules26051303 33671020 PMC7957565

[B277] Zhang J. Jiang C. S. (2018). Synthesis and evaluation of coumarin/piperazine hybrids as acetylcholinesterase inhibitors. Med. Chem. Res. 27, 1717–1727. 10.1007/s00044-018-2185-x

[B278] Zhang Y. W. Thompson R. Zhang H. Xu H. (2011). APP processing in Alzheimer’s disease. Mol. Brain 4, 3. 10.1186/1756-6606-4-3 21214928 PMC3022812

[B279] Zhang X. Wang Y. Wang S. N. Chen Q. Tu Y. Yang X. (2018). Discovery of a novel multifunctional carbazole–aminoquinoline dimer for Alzheimer’s disease: copper selective chelation, anti-amyloid aggregation, and neuroprotection. Med. Chem. Res. 27, 777–784. 10.1007/s00044-017-2101-9

[B280] Zhang H. Song Q. Yu G. Cao Z. Qiang X. Liu X. (2021a). Phthalimide-(N-alkylbenzylamine) cysteamide hybrids as multifunctional agents against Alzheimer’s disease: design, synthesis, and biological evaluation. Chem. Biol. Drug Des. 98, 493–500. 10.1111/cbdd.13905 34143938

[B281] Zhang Z. Guo J. Cheng M. Zhou W. Wan Y. Wang R. (2021b). Design, synthesis, and biological evaluation of novel xanthone-alkylbenzylamine hybrids as multifunctional agents for the treatment of Alzheimer’s disease. Eur. J. Med. Chem. 213, 113154. 10.1016/j.ejmech.2021.113154 33476932

[B282] Zhao J. Fu Y. Yasvoina M. Shao P. Hitt B. O’Connor T. (2007). Beta-site amyloid precursor protein cleaving enzyme 1 levels become elevated in neurons around amyloid plaques: implications for Alzheimer's disease pathogenesis. J. Neurosci. 27, 3639–3649. 10.1523/JNEUROSCI.4396-06.2007 17409228 PMC6672403

[B283] Zhao J. Deng Y. Jiang Z. Qing H. (2016). G protein-coupled receptors (GPCRs) in Alzheimer’s disease: a focus on BACE1 related GPCRs. Front. Aging Neurosci. 8, 58. 10.3389/fnagi.2016.00058 27047374 PMC4805599

[B284] Zhao X. Min D. Jiang Y. Guo D. Zhu Y. Deng Y. (2017). Multipotent AChE and BACE-1 inhibitors for the treatment of Alzheimer’s disease: design, synthesis and bio-analysis of 7-amino-1,4-dihydro-2H-isoquilin-3-one derivates. Eur. J. Med. Chem. 138, 738–747. 10.1016/j.ejmech.2017.07.006 28728106

[B285] Zheng Y. Qiang X. Xu R. Song Q. Tian C. Liu H. (2018). Design, synthesis and evaluation of pterostilbene β-amino alcohol derivatives as multifunctional agents for Alzheimer’s disease treatment. Bioorg Chem. 78, 298–306. 10.1016/j.bioorg.2018.03.016 29625269

[B286] Zhong C. López-Hernández G. Y. Talmage D. A. Role L. W. (2014). “Presynaptic nicotinic acetylcholine receptors and the modulation of circuit excitability,” in Nicotinic Receptors (New York: Springer), 137–167. 10.1007/978-1-4939-1167-7_7

[B287] Zhu G. Li X. Yang J. He Y. Mi J. Tang L. (2021). Development of novel 2-acetylphenol-O-alkylhydroxyethylamine derivatives as multifunctional agents for Alzheimer’s disease treatment. Med. Chem. Res. 30, 2016–2029. 10.1007/s00044-021-02786-7

