# Supplementary material for: Comprehensive medicinal chemistry survey highlights a portfolio of lead molecules for Alzheimer’s disease therapy
Source: Front Chem. 2025 Oct 1;13:1642190. doi: 10.3389/fchem.2025.1642190 (PMC12521189; doi:10.3389/fchem.2025.1642190)
Supplement: Supplementary file 1 [file Table1.docx]

**Table S1:** Cholinesterase inhibitors

| **Sr. No.** | **Compound** | **Activity** | | **Assay** | |
| --- | --- | --- | --- | --- | --- |
| **1.** |  | *AChE*  IC_50_ = 0.253 ± 0.016 nM  *BuChE*  IC_50_ = 10.8 ± 2.5 nM | | -Ellman’s assay | |
| **2.**  **3.** |  | *AChE*  IC_50_ = 6.4 ± 0.5 nM  *BuChE*  IC_50_ = 5.5 ± 1.8 nM) | | -Ellman’s assay | |
| **4.** |  | *bAChE*  IC_50_ = 34.1 ± 1.0 nM  *hAChE*  IC_50_ = 14.0 ± 1.2 nM  *hBChE*  IC_50_ = 1076 ± 78 nM  *AChE* selectivity  (77%) | | -Ellman’s assay | |
| **5.** |  | IC_50_ = 0.041 ± 0.001 µM | | *in-vitro* *EeAChE* inhibition | |
| **6.**  **7.** |  | IC_50_ = 0.521 ± 0.025 µM  IC_50_ = 0.055 ± 0.012 µM | | *in-vitro* *AChE* inhibition  *in-vitro* *BuChE* inhibition | |
| **8.** |  | IC_50_ = 0.47 ± 0.09 µM | | *in-vitro* *AChE* inhibition | |
| **9.** |  | IC_50_ = 0.521 ± 0.025 µM | | *in-vitro* *AChE* inhibition | |
| **10.** |  | | IC_50_ = 0.0745 ± 0.0031 μM | |  |
| **11.** |  | | *AChE*  IC_50_ = 3.28 µM  *BuChE*  IC_50_ = 2.19 µM | |  |
| **12.** |  | | IC_50_ = 0.09 ± 0.02 μM | |  |
| **13.** |  | | IC_50_ = 8.0 ± 0.37 µM | |  |
| **14.** |  | | *AChE*  IC_50_ = 0.65 ± 0.06 μM  *BuChE*  IC_50_ = 1.32 ± 0.06 μM | |  |
| **15.** |  | | IC_50_ = 8.78 ± 0.22 μM | |  |
| **16.** |  | | IC_50_ = 0.091 ± 0.011 μM | |  |
| **17.** |  | | IC_50_ = 6.21 ± 0.03 μM | |  |
| **18.** |  | | *AChE*  IC_50_ = 0.04 ± 0.01 μM  *BuChE*  IC_50_ = 0.68 ± 0.07 μM | |  |
| **19.** |  | | *AChE*  IC_50_ =10.30 ± 1.05 μM  *BuChE*  IC_50_ = 3.66 ± 0.11 μM | |  |
| **20.** |  | | IC_50_ = 0.40 ± 0.03 µM | |  |
| **21.** |  | | *EeAChE*  IC_50_ = 0.064 ± 0.004 μM  *hAChE*  IC_50_ = 1.25 ± 0.26 μM  *BuChE*  IC_50_ = 42.05 ± 2.94 μM | |  |
| **22.** |  | | IC_50_ = 0.073 ± 0.003 µM | |  |
| **23.** |  | | IC_50_ = 4.34 ± 0.46 μM | |  |
| **24.**  **25.** |  | | *AChE*  IC_50_ = 16.5 μM  *BuChE*  IC_50_ = 0.59 μM    *AChE*  IC_50_ = 26.5 μM  *BuChE*  IC_50_ = 0.18 μM | |  |
| **26.** |  | | IC_50_ = 0.044 ± 0.002 μM | |  |
| **27.**  **28.** |    | | IC_50_ = 1.51 ± 0.25 μM  IC_50_ = 3.78 ± 0.63 μM | |  |
| **29.** |  | | *AChE* inhibition = 56.73% | |  |
| **30.** |  | | IC_50_ = 0.10 ± 0.01 μM | |  |
| **31.** |  | | *AChE*  IC_50_ = 7.23 ± 0.16 µM  *BuChE*  IC_50_ = 90.76 ± 0.21 µM | |  |
| **32.** |  | | IC_50_ = 13.72 ± 0.01 µM | |  |
| **33.** |  | | *AChE*  IC_50_ = 5.61 ± 0.22 µM  *BuChE*  IC_50_ = 0.87 ± 0.03 µM | |  |
| **34.** |  | | *AChE*  IC_50_ = 0.39 ± 0.15 μM  *BuChE*  IC_50_ = 0.16 ± 0.04 μM | |  |
| **35.** |  | | *AChE*  IC_50_ = 0.069 ± 0.005 µM  *BuChE*  IC_50_ = 1.35 ± 0.07 µM | |  |
| **36.** |  | | IC_50_ = 1.5609 ± 0.0237 mM | |  |
| **37.** |  | | IC_50_ = 0.88 ± 0.04 μM | |  |
| **38.** |  | | IC_50_ = 0.85 ± 0.043 μM | |  |
| **39.** |  | | *AChE*  75.52 ± 1.76%  *BuChE*  62.03 ± 1.82% | |  |
| **40.** |  | | *AChE*  IC_50_ = 0.82 ± 0.05 µM | |  |
| **41.** |  | | IC_50_ = 0.0035 ± 0.005 µM | |  |
| **42.** |  | | inhibition = 41.9 ± 7.3% | |  |
| **43.** |  | | IC_50_ = 18.2 ± 1.2 μM | |  |
| **44.**  **45.** |  | | *AChE*  IC_50_ = 0.204 μM | |  |
| **46.** |  | | *AChE*  IC_50_ = 0.30 ± 0.01 nM  *BuChE*  IC_50_ = 1.84±0.03 nM | |  |
| **47.** |  | | μ moles of *AChE/*min/mg protein (0.0027 ± 0.0006) | |  |
| **48.** |  | | *hAChE*  IC_50_ = 0.173 uM | |  |
| **49.**  **50.** |  | | IC_50_ = 5.68 μM,  IC_50_ = 0.81 μM | |  |
| **51.** |  | | *AChE*  IC_50_ = 0.56 ± 0.02 uM  *BuChE*  IC_50_ =1.17 ± 0.09 uM | |  |
| **52.** |  | | *AChE*  Ki = 0.6 μM  *BuChE*  Ki = 0.37μM  BACE  Ki = 1.24 μM | |  |
| **53.** |  | | *AChE*  IC_50_ = 5.80 ± 0.70 uM  *BuChE*  IC_50_ = 0.96 ± 0.31uM | |  |
| **54.**  **55.** |  | | **54**: 0.018 ± 0.001 μM  **55**: 0.022 ± 0.002 μM | |  |
| **56.** |  | | K_i_ =73 μM | |  |
| **57.**  **58.**  **59.** |  | | IC_50_ = 9.97±0.71 μM | |  |
| **60.** |  | | - | |  |
| **61.** |  | | *AChE*  IC_50_ = 298 ± 43 μM  *BuChE*.  IC_50_ = 321 ± 29 μM | |  |
| **62.** |  | | IC_50_ = 0.17±0.02 uM | |  |
| **63.** |  | | IC_50_ = 1.5±0.12 uM | |  |
| **64.** |  | | *AChE*  IC_50_ = 0.12 ± 0.03  *BuChE*  IC_50_ = 0.13 ± 1.75 | |  |
