# Supplementary material for: Comprehensive medicinal chemistry survey highlights a portfolio of lead molecules for Alzheimer’s disease therapy
Source: Front Chem. 2025 Oct 1;13:1642190. doi: 10.3389/fchem.2025.1642190 (PMC12521189; doi:10.3389/fchem.2025.1642190)
Supplement: Supplementary file 2 [file Table2.docx]

**Table S2:** Dual-target Inhibitors

| **Sr. No.** | **Compound** | **Activity** | **Assay type** |
| --- | --- | --- | --- |
| **81** |  | *AChE* inhibition IC_50_ = 0.55 ± 0.034 nM  Aβ aggregation inhibition = 39.4% | *- in-vitro* *AChE* inhibition  - Thioflavin-T assay |
| **82** |  | *hAChE*  IC_50_ =1.62 ± 0.10 nM  Aβ aggregates = 61.8% | *- in-vitro* *AChE* inhibition  - Thioflavin-T assay |
| **83** |  | *hAChE*  IC_50_ = 8.8 ± 0.4 µM  Aβ self-aggregation inhibition  IC_50_ = 31.2 ± 9.0 µM | *- in-vitro* *AChE* inhibition  - Thioflavin-T assay |
| **84** |  | *AChE*  IC_50_ = 27.32 ± 1.61 nM  Aβ aggregation inhibition = 82.5 ± 10.4% | *in-vitro* *AChE* inhibition  - Thioflavin-T assay |
| **85** |  | *AChE* inhibitory activity  IC_50_ = 6.3 nM  Cu-induced Aβ aggregation inhibition = 39.4% | *- in-vitro* *AChE* inhibition  - Thioflavin-T assay |
| **86** |  | *hAChE*  IC_50_ = 0.027 ± 0.002 μM  Aβ aggregation inhibition = 40.19 ± 2.39% | *- in-vitro* *AChE* inhibition  - Thioflavin-T assay |
| **87** |  | *- AChE* inhibition  IC_50_ = 9.10 µM  - MAO-B inhibition  IC_50_ = 0.30 µM | *- in-vitro* *AChE* inhibition  - MAO inhibition assay |
| **88** |  | *- AChE* inhibition  IC_50_ = 3.04 ± 0.32 μM  - MAO-B inhibition  IC_50_ = 27.03 ± 0.50 μM | *- in-vitro* *AChE* inhibition  - MAO inhibition assay |
| **89** |  | - MAO-A inhibition  IC_50_ = 3.275 ± 0.040 µM  - MAO-B inhibition  IC_50_ = 0.027 ± 0.004 µM  - anti-aggregation  = 54% | - Thioflavin-T assay  - MAO inhibition assay |
| **90** |  | *hAChE* inhibition potency IC_50_= 0.35 ± 0.03 µM  self-induced Aβ aggregation inhibition = 35.0% | *- in-vitro* *AChE* inhibition  - Thioflavin-T assay |
| **91** |  | *AChE*  IC_50_ = 0.01 ± 0.001 μM  self-induced Aβ aggregation = 49.2% | *- in-vitro* *AChE* inhibition  - Thioflavin-T assay |
| **92** |  | *AChE*  IC_50_ = 0.07 ± 0.01 µM  self-induced Aβ aggregation inhibition = 59.2 ± 1.6% | *in-vitro* *AChE* inhibition assay  ThT fluorescence assay |
| **93** |  | *- AChE* inhibition  IC_50_ = 16.17 ± 0.02 µM  - BACE-1 inhibition  IC_50_ = 7.99 ± 0.916 µM | *- in-vitro* *AChE* inhibition  - FRET assay |
| **94** |  | *- hAChE* inhibition  IC_50_ = 3.69 ± 0.24 μM  - MAO-B inhibition  IC_50_ = 0.63 ± 0.01 μM | *- in-vitro* *AChE* inhibition  - MAO inhibition assay |
| **95** |  | *- AChE* inhibition  IC_50_ = 0.86 ± 0.04 μM  *- BuChE* inhibition  IC_50_ = 6.03 ± 0.34 μM  - BACE-1 inhibition  IC_50_ = 19.60 ± 0.9 μM | *- in-vitro* *AChE* inhibition  - FRET assay |
| **96** |  | *hBuChE* inhibition  IC_50_ = 0.215 µM | *- in-vitro* *AChE* inhibition |
| **97** |  | *- AChE* inhibition  IC_50_ =18.93 ± 1.02 pM  - BACE-1 inhibition  = 97.68% | *- in-vitro* *AChE* inhibition  - FRET assay |
| **98** |  | *- AChE* inhibition  IC_50_ = 0.028 ± 0.001 μM  - MAO-B inhibition  IC_50_ = 0.046 ± 0.002 μM | *- in-vitro* *AChE* inhibition  - MAO inhibition assay |
| **99** |  | *AChE*  IC_50_ = 0.06 ± 0.03 µM  *BuChE*  IC_50_ = 28.04 ± 1.71 µM  self-induced Aβ aggregation = 32.4% | *- in-vitro* *AChE* inhibition  - Thioflavin-T assay |
| **100** |  | *hAChE* inhibition IC_50_ = 0.039 ± 0.002 µM  self-induced Aβ aggregation inhibition = 57.1% | *- in-vitro* *AChE* inhibition  - Thioflavin-T assay |
| **101** |  | *EeAChE* IC_50_ = 1.55 ± 0.17 μM  *hAChE*  IC_50_ = 2.23 ± 0.04 μM  self-induced Aβ aggregation inhibition = 36.08% | *- in-vitro* *AChE* inhibition  - Thioflavin-T assay |
| **102** |  | *BuChE*  IC_50_ = 0.72 ± 0.038 µM  Aβ aggregation inhibition = 72.5% | *- in-vitro* *AChE* inhibition  - Thioflavin-T assay |
| **103** |  | *- hAChE* inhibition  IC_50_ = 1.2 ± 0.07 µM  - MAO-B inhibition  IC_50_ = 2.6 ± 0.05 µM | *- in-vitro* *AChE* inhibition  - MAO inhibition assay |
| **104** |  | *AChE*  IC_50_ = 2.33 ± 0.021 µM    *BuChE*  IC_50_ = 1.08 ± 0.011 µM  Aβ aggregation inhibition = 50.3 ± 2.4% | *- in-vitro* *AChE* inhibition  - Thioflavin-T assay |
| **105** |  | *EeAChE* IC_50_ = 3.04 ± 0.94 μM  Aβ aggregation inhibition = 77.5% | *- in-vitro* *AChE* inhibition  - Thioflavin-T assay |
| **106** |  | *BuChE*  IC_50_ = 0.45 ± 0.05 µM  *AChE*  IC_50_ = 2.1 ± 0.1 µM  self-induced Aβ aggregation inhibition = 46.4% | *- in-vitro* *AChE* inhibition  - Thioflavin-T assay |
| **107** |  | *AChE* inhibitory activity  IC_50_ = 76 µM  self-induced Aβ aggregation inhibition = 36.1% | *- in-vitro* *AChE* inhibition  - Thioflavin-T assay |
| **108** |  | *- AChE* inhibition  IC_50_ = 0.086 ± 0.01 µmolL^-1^  *- BuChE* inhibition  IC_50_ = 16.450 ± 2.12 µmolL^-1^  - BACE-1 inhibition  IC_50_ = 0.043 ± 0.01 µmolL^-1^ | *- in-vitro* *AChE* inhibition  - FRET assay |
| **109** |  | *- AChE* inhibition  IC_50_ = 0.089 ± 0.01 μM  - MAO-B inhibition  IC_50_ = 149.21 ± 3.39 μM | *- in-vitro* *AChE* inhibition  - MAO inhibition assay |
| **110** |  | *AChE*  IC_50_ = 0.059 ± 0.003 µM  self-induced Aβ aggregation inhibition = 83.7% | *- in-vitro* *AChE* inhibition  - Thioflavin-T assay |
| **111** |  | *eqBuChE* IC_50_ = 2.13 ± 0.01 µM  *rat BuChE* IC_50_ = 1.8 ± 0.02 µM  *hBuChE*  IC_50_ = 3.82 ± 0.05 µM  Aβ aggregation inhibition = 50.8% | *- in-vitro* *AChE* inhibition  - Thioflavin-T assay |
| **112** |  | *BuChE*  IC_50_ = 39.56 μM  Aβ anti-aggregation = 67.78% | *- in-vitro* *AChE* inhibition  - Thioflavin-T assay |
| **113** |  | *AChE*  IC_50_ = 0.049 µM  self-induced Aβ aggregation = 25.5% | *- in-vitro* *AChE* inhibition  - Thioflavin-T assay |
| **114** |  | *EeAChE*  IC_50_ = 4.64 ± 0.23 µM  *hAChE*  IC_50_ = 5.42 ± 0.25 µM  self-induced Aβ aggregation = 56.2% | *- in-vitro* *AChE* inhibition  - Thioflavin-T assay |
| **115** |  | *hAChE*  IC_50_ = 7.61 ± 0.53 µM  self-induced Aβ aggregation inhibition = 63.9% | *- in-vitro* *AChE* inhibition  - Thioflavin-T assay |
| **116** |  | *hAChE*  IC_50_ = 0.153 ± 0.016 µM  self-induced Aβ aggregation inhibition = 26.5% | *- in-vitro* *AChE* inhibition  - Thioflavin-T assay |
| **117** |  | *AChE*  IC_50_ = 0.065 ± 0.002 µM  self-induced Aβ aggregation = 75.32% | *- in-vitro* *AChE* inhibition  - Thioflavin-T assay |
| **118** |  | *EeAChE*  IC_50_ = 24.04 ± 1.48 μM  self-induced Aβ aggregation inhibition = 40.23% | *- in-vitro* *AChE* inhibition  - Thioflavin-T assay |
| **119** |  | *EeAChE*  IC_50_ = 0.34 ± 0.16 μM  self-induced Aβ aggregation = 57.5% | *- in-vitro* *AChE* inhibition  - Thioflavin-T assay |
| **120** |  | *AChE*  IC_50_ = 47.33 ± 0.02 nM  *BuChE* IC_50_ = 159.43 ± 0.72 nM  Aβ aggregation inhibition = 51.3% | *- in-vitro* *AChE* inhibition  - Thioflavin-T assay |
| **121** |  | *EeAChE*  IC_50_ = 1.09 ± 0.02 μM  self-induced Aβ aggregation inhibition = 25.0% | *- in-vitro* *AChE* inhibition  - Thioflavin-T assay |
| **122** |  | *- hAChE* inhibition  IC_50_ = 4.11 ± 0.12 nM  - BACE-1 inhibition  IC_50_ = 18.3 ± 0.17 nM | *- in-vitro* *AChE* inhibition  - FRET assay |
| **123** |  | *- hAChE* inhibition  IC_50_ = 0.064 ± 0.006 μM  *- hBuChE* inhibition  IC_50_ = 0.074 ± 0.016 μM  - BACE-1 inhibition  IC_50_ = 0.143 ± 0.024 μM | *- in-vitro* *AChE* inhibition  - FRET assay |
| **124** |  | *- hAChE* inhibition  IC_50_ = 0.11 ± 0.02 μM  - BACE-1 inhibition  IC_50_ = 0.22 ± 0.02 μM | *- in-vitro* *AChE* inhibition  - FRET assay |
| **125** |  | *- AChE* inhibition  IC_50_ = 16.07 ± 0.07 μM  *- BuChE* inhibition  IC_50_ = 15.16 ± 0.22 μM  - BACE-1 inhibition  = 24.3% | *- in-vitro* *AChE* inhibition  - FRET assay |
| **126** |  | *- AChE* inhibition  IC_50_ = 1.75 ± 0.12 µM  *- BuChE* inhibition  IC_50_ = 0.69 ± 0.12 µM  - MAO-A inhibition  IC_50_ = 3.5 ± 0.2 µM  - MAO-B inhibition  IC_50_ = 6.0 ± 0.4 µM | *- in-vitro* *AChE* inhibition  - MAO inhibition assay |
| **127** |  | *- eeAChE* inhibition  IC_50_ = 0.96 ± 0.01 µM  - MAO-B inhibition  IC_50_ = 6.8 ± 0.31 µM | *- in-vitro* *AChE* inhibition  - MAO inhibition assay |
| **128** |  | - MAO-B inhibition  IC_50_ =1.73 ± 0.39 µM  - anti-aggregation  = 91.3% | - Thioflavin-T assay  - MAO inhibition assay |
| **129** |  | - MAO-A inhibition  IC_50_ = 6.64 ± 0.41 μM  - anti-aggregation  = 67.4% | - Thioflavin-T assay  - MAO inhibition assay |
